# Trends and Disparities in Diet Quality and Nutrient Intake among US Adults by Bodyweight Status

**DOI:** 10.3390/nu16162793

**Published:** 2024-08-21

**Authors:** Wenbo Gu, Yi Yang, Liuying Wang, Yuhua Song, Xuemin Yan, Zhen Tian, Changhao Sun

**Affiliations:** 1 Department of Nutrition and Food Hygiene, School of Public Health, Harbin Medical University, 157 Baojian Road, Harbin 150081, China; wenbogu@hrbmu.edu.cn (W.G.); 2023020154@hrbmu.edu.cn (Y.Y.); 202301106@hrbmu.edu.cn (Y.S.); glacier@hrbmu.edu.cn (X.Y.); 202101055@hrbmu.edu.cn (Z.T.); 2 School of Health Management, Harbin Medical University, 157 Baojian Road, Harbin 150081, China; wangliuying@hrbmu.edu.cn

**Keywords:** dietary quality, nutrient intake, trend, overweight, obesity

## Abstract

The prevalence of obesity has been increasing in the US. Among the multifactorial contributors to obesity, dietary factors stand out as primary drivers. Using data from NHANES, we investigated the trends and disparities in diet quality and nutrient intake among US adults with different bodyweight statuses. Participants were divided into normal weight, overweight, and obese groups based on BMI. Diet quality was examined using HEI-2020. Nutrient intake was estimated based on the USDA Food and Nutrient Database for Dietary Studies. From 1999 to 2020, Despite an overall improvement in diet quality among overweight and obese US adults, disparities persisted for most HEI-2020 components, and worsened for whole grains, seafood and plant proteins, and fatty acids between normal weight and obese participants. Overweight and obese participants tended to consume less energy from total carbohydrates and more from total fat. The estimated total energy intake increased among obese participants over the past two decades, while no significant changes were observed among normal weight and overweight participants. In the 2017–2020 period, obese participants had lower HEI-2020 scores than both normal weight and overweight participants. However, no significant differences in total energy intake were observed among normal weight, overweight, and obese populations.

## 1. Introduction

Since the 1980s, obesity prevalence has been increasing among adults, serving itself as a fertile ground for various metabolic diseases such as hypertension, dyslipidemia, type 2 diabetes mellitus, cardiovascular diseases, and several cancers [1,2]. Among the multifactorial contributors to obesity, dietary factors stand out as primary drivers [3,4]. In recent years, attention has increasingly turned towards healthy dietary patterns, both in terms of food and nutrient intake, for their potential in facilitating weight loss [5,6,7]. Notably, from 1999 to 2016, there has been an increasing trend in overall diet quality across the total population, encompassing both youths and adults in the United States [8,9]. This period has also witnessed a significant surge in the proportion of individuals reporting consuming a healthy diet as their preferred weight loss strategy [10]. However, among adults with overweight or obesity, little emphasis has been placed on demonstrating their dietary characteristics. Previous research has revealed the disparities that exist in diet quality between obese participants and their normal weight counterparts [11], while potential trends in these dietary disparities over time remain less clear. Utilizing nationally representative data from ten consecutive 2-year cycles of the National Health and Nutrition Examination Survey (NHANES) from 1999 to 2000 to 2017 to 2020, our aim is to assess whether disparities in overall diet quality and nutrient intake have persisted, improved, or worsened over the past two decades among adults categorized as normal weight, overweight, and obese.

## 2. Materials and Methods

### 2.1. Study Design and Population

The NHANES comprises a series of multistage and stratified surveys that are representative of the national population of the United States. Using NHANES data from 10 consecutive cycles spanning the period from 1999 to 2000 to 2017 to 2020, we selected adult participants (age ≥ 20) with a body mass index (BMI) ≥ 18.5. The response rates of the NHANES ranged from 46.9% in 1999–2000 to 80% in 2017–2020. During the study period, 88.46% (47,467 of 53,659) of NHANES respondents provided a single valid diet recall, of whom 77.00% (36,549 of 47,467) also provided a second valid recall. Normal weight is defined as a body mass index (BMI) ranging from 18.5 to 24.9, overweight is defined as a BMI ranging from 25 to 29.9, and obesity is defined as a BMI ≥ 30. The protocol was approved by the National Center for Health Statistics (NCHS) ethics review board, and written informed consent was provided by all participants. The detailed methodology and protocols are available on the NHANES website (https://www.cdc.gov/nchs/nhanes/about_nhanes.htm (accessed on 19 July 2024).

### 2.2. Assessment of Dietary Quality

Dietary data were assessed using 24 h recalls. From 1999 to 2002, a 24 h dietary recall was conducted at the Mobile Examination Center. From 2003 to 2020, a second 24 h recall was administered via a telephone interview approximately 3 to 10 days after the initial recall [12,13]. The Healthy Eating Index (HEI) 2020 is a commonly used composite measure of diet quality and is assessed for alignment with the US Department of Agriculture (USDA) 2020–2025 Dietary Guidelines for Americans. Intakes of individual food components were calculated on a density basis as amount per 1000 kcal. The HEI-2020 was composed of a total of 13 dietary components, with 9 adequacy components, including total fruits, whole fruits, total vegetables, greens and beans, whole grains, dairy, total protein foods, seafood and plant proteins, and fatty acids, along with 4 moderation components, including refined grains, sodium, added sugar, and saturated fats. The HEI-2020 score ranges from 0 to 100, with the details of calculation methods documented in Table S1.

### 2.3. Nutrients and Energy Intake

In addition to the 13 HEI-2020 dietary components, we evaluated individual nutrients associated with major health outcomes for the general public. Nutrient intake was estimated based on cycle-specific versions of the USDA Food and Nutrient Database for Dietary Studies. Total energy intake was estimated as the sum of energy from carbohydrates, protein, and fat. Energy percentages of macronutrients were computed as a proportion of energy from each macronutrient over total energy intake. For total carbohydrate, the percentage of energy = (total carbohydrate [g] × 4 [kcal/g])/total energy intake (kcal) × 100; for protein, the percentage of energy = (protein [g] × 4 [kcal/g])/total energy intake (kcal) × 100; and for total fat, the percentage of energy = (total fat [g] × 9 [kcal/g])/total energy intake (kcal) × 100.

### 2.4. Statistical Analysis

All analyses incorporated sample weights, stratification, and clustering to account for the complex survey design, following the NHANES analytic guidelines. Weighted means and 95% confidence intervals were estimated for diet quality and nutrient intake among total, normal weight, overweight, and obese participants by cycle. General linear regression was used to estimate trends by treating each 2-year survey cycle as a continuous variable. Absolute differences in estimated means were calculated between the 1999–2000 and 2017–2020 cycles. A Weighted Wald test was used to test for an interaction between 2-year survey cycle and bodyweight status, as well as for an interaction between the change from 1999 to 2000 and bodyweight status. To determine the potential role of demographic shifts, sensitivity analyses adjusting for demographic variables (age, sex, race/ethnicity, education, and income) were performed, and subgroup analyses were performed on each demographic subpopulation. All tests were two-tailed, with *p* < 0.05 considered to be statistically significant. For multiple comparisons, Holm-Bonferroni method was used to correct *p* values. R software (version 4.1.2) was used for all analyses.

## 3. Results

### 3.1. Participants Characteristics

The survey included 34,629 respondents. The mean age of the participants was 47.5 [0.2] years, with 53.2% being female and 69.7% being non-Hispanic White. Survey-weighted characteristics of total population, normal weight, overweight, and obese participants were presented in Table 1. Obese participants were older (mean age 48.3 [0.3]) than normal weight participants (mean age 44.5 [0.3]), they were more likely to be male (45.0% in obese participants vs. 40.3% in normal weight participants), and were less likely to be non-Hispanic White (66.2% in obese participants vs. 72.3% in normal weight participants). Trends in sociodemographic characteristics shifts of American adults by bodyweight status were shown in Table S2.

### 3.2. Trends in HEI-2020 Score by Bodyweight Status

From 1999 to 2020, there was a modest yet significant improvement in the mean HEI-2020 scores among total participants (increasing from 45.4 [95% CI, 43.6–47.1] to 50.1 [95% CI, 49.2–51.0], *p* for trend < 0.001), normal weight participants (increasing from 46.6 [95% CI, 44.4–48.9] to 51.6 [95% CI, 49.7–53.5], *p* for trend = 0.010), overweight participants (increasing from 45.6 [95% CI, 43.5–47.6] to 51.1 [95% CI, 50.2–52.1], *p* for trend < 0.001), and obese participants (increasing from 43.8 [95% CI, 42.0–45.7] to 48.4 [95% CI, 47.5–49.3], *p* for trend < 0.001) (Table 2). Notably, normal weight and obese participants exhibited less improvement in HEI-2020 scores compared to overweight participants (change in mean score = 4.97 [95% CI, 2.02–7.93] and 4.55 [95% CI, 2.47–6.63] vs. 5.58 [95% CI, 3.31–7.85, respectively; *p* < 0.001 for interaction]).

### 3.3. Trends in Dietary Components of HEI-2020 Score by Bodyweight Status

Five out of thirteen dietary components of HEI-2020 showed increasing trends among overweight and obese participants, including greens and beans, whole grains, seafood and plant proteins, refined grains, and added sugars. Particularly, the whole grain score significantly improved in overweight (from 1.7 [95% CI, 1.3–2.0] to 2.4 [95% CI, 2.2–2.7]; *p* for trend = 0.007), and obese participants (from 1.3 [95% CI, 1.1–1.4] to 2.3 [95% CI, 2.1–2.4]; *p* for trend < 0.001). Similarly, the added sugars score significantly increased in overweight (from 4.5 [95% CI, 4.1–4.8] to 7.2 [95% CI, 7.0–7.4]; *p* for trend < 0.001), and obese participants (from 3.9 [95% CI, 3.4–4.4] to 6.9 [95% CI, 6.6–7.1]; *p* for trend < 0.001). Conversely, other dietary components such as total vegetables, dairy, total protein foods, sodium, and saturated fats exhibited decreased trends among overweight and obese participants. Specifically, the dairy score significantly decreased in overweight (from 5.4 [95% CI, 5.2–5.7] to 4.8 [95% CI, 4.6–5.0]; *p* for trend < 0.001), and obese participants (from 5.9 [95% CI, 5.2–6.6] to 4.8 [95% CI, 4.6–5.1]; *p* for trend = 0.017). From 1999 to 2000, disparities persisted for most HEI-2020 components, and worsened for whole grains, seafood and plant proteins, and fatty acids between normal weight and obese participants. Further details are provided in Table 2. After adjusting for differences in age, sex, race/ethnicity, education, and income, the scores remained largely unchanged for most dietary components (Tables S3 and S4).

### 3.4. Trends in Mean Intake of HEI-2020 Dietary Components and Key Nutrients among US Adults by Bodyweight Status

Mean intakes of total protein foods, seafood and plant proteins, added sugars, and polyunsaturated fat among obese individuals were comparable or even more favorable than those of normal weight individuals in 1999–2000. However, by 2017–2020, these advantages have declined. For example, the mean intake of seafood and plant proteins were 1.78 [95% CI, 1.31–2.25] among normal weight participants and 1.94 [95% CI, 1.51–2.37] among obese participants in 1999–2000, but in 2017–2020, were 1.00 [95% CI, 0.85–1.15] among normal weight participants and 0.79 [95% CI, 0.73–0.85] among obese participants. Furthermore, disparities in dietary intake worsened for other food and nutrient groups such as greens and beans, whole grains, and dietary fiber among obese individuals compared to those of normal weight. For instance, the mean intake of dietary fiber increased from 12.68 [95% CI, 11.70–13.66] in 1999–2000 to 16.21 [95% CI, 15.32–17.10] in 2017–2020 among normal weight participants (*p* for trend < 0.001), and increased from 11.62 [95% CI, 10.47–12.77] in 1999–2000 to 14.84 [95% CI, 14.30–15.39] in 2017–2020 among obese participants (*p* for trend < 0.001). More details are provided in Table S5. Further adjustment for age, sex, and race/ethnicity did not alter the results (Table S6). Mean change in key food groups intake from 1999 to 2020 remained similar among subgroups of obese participants by age, sex, race/ethnicity, and education (Tables S7–S10). In 2017–2020 cycle, obese participants exhibited a lower HEI-2020 score than both normal weight and overweight participants. Compared to normal weight participants, obese participants showed 31.3% lower intakes of greens and beans, 23.5% lower intakes of whole grains, 21.0% lower intakes of seafood and plant proteins, 9.3% lower intakes of total vegetables, 8.5% lower intakes of dietary fiber, and 8.2% higher intakes of cholesterol. No significant differences in total energy intake were observed among participants with different bodyweight status. Other details are documented in Table 3.

### 3.5. Trends in Energy Intake from Total Carbohydrate, Protein, and Total Fat by Obesity Status

From 1999 to 2000, there were significant increases in total energy intake among the total population (from 1856.6 [95%CI, 1769.6–1943.5] to 2017.4 [95%CI, 1985.8–2049.0], *p* for trend = 0.007) and obese participants (from 1807.2 [95%CI, 1674.1–1940.3] to 2036.4 [95%CI, 1984.2–2088.7], *p* for trend = 0.017). However, no significant change was observed in total energy intake among normal weight and overweight participants (*p* for trend = 0.053, *p* for trend = 0.406, individually) (Table 4). The estimated percentage of energy intake from total carbohydrate declined from 52.4 [95%CI, 51.5–53.3] to 46.8 [95%CI, 45.9–47.7] (*p* for trend < 0.001) among normal weight participants, from 50.4 [95%CI, 49.0–51.7] to 46.3 [95%CI, 45.4–47.2] (*p* for trend < 0.001) among overweight participants, and from 50.3 [95%CI, 49.1–51.5] to 45.8 [95%CI, 45.2–46.4] (*p* for trend < 0.001) among obese participants. Conversely, the estimated percentage of energy intake from total fat increased from 31.1 [95%CI, 30.2–32.0] to 35.7 [95%CI, 35.1–36.4] (*p* for trend < 0.001) among normal weight participants, from 31.8 [95%CI, 31.1–32.6] to 36.4 [95%CI, 35.7–37.1] (*p* for trend < 0.001) among overweight participants, and from 33.4 [95%CI, 32.1–34.7] to 36.6 [95%CI, 36.2–37.1] (*p* for trend < 0.001) among obese participants. Additionally, small yet significant increases in the estimated percentage of energy intake from protein were observed among normal weight and obese participants.

## 4. Discussion

This study investigated recent trends in diet quality and nutrient intake among normal weight, overweight, and obese US adults. Overall, despite improvements in dietary quality among overweight and obese participants over the past two decades, disparities still existed between obese and normal weight individuals. Moreover, a significant increase in total energy intake was observed among obese participants, but not among normal weight or overweight participants. In 2017–2020 cycle, obese participants exhibited a lower HEI-2020 score compared to both normal weight and overweight participants. No significant differences in total energy intake were observed among normal weight, overweight, and obese participants.

Over the past two decades, overweight and obese populations have shown a significant decrease in the consumption of refined grains and added sugar, along with an increase in dietary fiber intake, trends that parallel with those observed in the normal weight population. However, there has been a concerning decline in the consumption of fruits, vegetables, and dairy across all bodyweight groups. Overall, diet quality and nutrient intake have improved among overweight and obese populations. This improvement may be attributed to the influence of dietary guidelines in shaping long-term dietary behavior in the US [14]. Additionally, the growing proportion of individuals with overweight and obesity who perveived themselves as overweight may also contribute to these positive changes [10]. These self-perceived overweight individuals tend to lose weight by reducing energy consumption, exercising, drinking a large volume of water, and improving their diet quality [15,16]. Nevertheless, despite these efforts, the rates of overweight and obesity continue to rise, suggesting that some may struggle with adherence or fail to apply sufficient effort, leading to unsatisfactory results [10,17]. Additionally, in 2017–2020 cycle, disparities in the consumption of certain foods and nutrients persisted between normal weight and obese populations, indicating that greater efforts are needed to achieve weight loss in the obese population.

Interestingly, we observed increasing trends in both overall diet quality and total energy intake among obese individuals. While obese individuals in 2017–2020 cycle tend to choose healthier foods compared to their counterparts from 1999–2020 cycle, they may overlook the importance of controlling calorie intake. Evidence from cohort studies indicates that maintaining a high-quality diet over the long term can markedly decrease the risk of adiposity [18,19,20,21], but excessive energy intake may counteract these benefits. Additionally, in 2017–2020 cycle, no significant differences in total energy intake were observed across different bodyweight groups. However, previous research suggests that, despite consuming equivalent amounts of energy, obese individuals may be more prone to fat accumulation due to robust energy compensation and inefficient energy expenditure, rendering weight loss more challenging compared to normal weight individuals [22].

Taken together, our findings suggest that for overweight and obese individuals striving to lose weight through dietary strategies, it is essential not only to prioritize diet quality, but also to reduce overall calorie intake. They are recommended to consume more low-energy-dense foods, such as whole grains and dietary fiber, which help enhance satiety and reduce energy intake [23,24]. Moreover, when developing weight loss strategies, particularly those centered around dietary changes, it is crucial to consider individual’s preferences and capabilities to ensure sustained adherence over the long term [25]. 

This study has several limitations. First, measurement error cannot be avoided due to the characteristics of self-reported dietary 24 h recall data. To address this issue, the recall was assisted by a professional interviewer and the NCI method was used to ensure the collection of accurate information and improve the estimates of food intake. Second, although small yet significant differences in temporal trends were observed in some HEI-2020 components, the clinical significance remains to be verified. Third, BMI levels in individuals can fluctuate over a short period, increasing the probability of misclassification of bodyweight status. Fourth, in our study, dietary trends were evaluated based on the population rather than a direct evaluation of dietary changes among individuals. Fifth, temporal relationships and causality cannot be analyzed due to the cross-sectional nature of the data. Sixth, although we conducted our analysis using the most recent data available, it remains uncertain whether the findings observed are applicable to the diet quality and nutrient intake among adults with different bodyweight statuses in 2024. Finally, while dietary factors stand out as primary drivers of obesity, other obesity-related factors, such as behavioral (physical activity, sedentary lifestyle, and sleep), environmental (access to food and food choices), physiological (medication use), genetic, social, and economic factors were not included in the current study, and further investigation is needed.

## 5. Conclusions

Despite an overall improvement in diet quality among overweight and obese US adults over the past two decades, disparities existed or worsened between obese and normal weight populations. Significant increases in total energy intake were observed among the obese population, but not among those with normal weight or overweight. Our findings can help individuals with overweight or obesity better understand their dietary characteristics and the disparities compared to those of normal weight individuals. We highlight the need for them to make greater efforts to improve diet quality and manage calorie intake. Furthermore, this study suggests that BMI should be taken into account when developing dietary guidelines to provide more effective recommendations for different populations.

## Figures and Tables

**Table 1:** Sociodemographic characteristics of US adults by bodyweight status, 1999–2020 ^a^.

Sociodemographic Characteristics	Survey-Weighted % (95% CI)			
	Normal Weight (*n* = 9428)	Overweight (*n* = 11,723)	Obesity (*n* = 13,478)	
Age, Mean (95% CI), y	44.5 (43.9–45.2)	49.3 (48.7–49.8)	48.3 (47.8–48.8)	
Age group, y				
20–34	35.8 (34.0–37.6)	23.0 (21.7–24.4)	23.4 (22.1–24.8)	
35–49	26.8 (25.3–28.5)	29.3 (27.9–30.7)	29.3 (27.9–30.7)	
50–64	21.3 (19.7–23.0)	26.5 (25.0–28.0)	29.1 (27.7–30.4)	
≥65	16.1 (15.0–17.2)	21.2 (19.9–22.6)	18.3 (17.2–19.4)	
Sex				
Male	40.3 (38.8–41.8)	54.6 (53.3–55.9)	45.0 (43.6–46.4)	
Female	59.7 (58.2–61.2)	45.4 (44.1–46.7)	55.0 (53.6–56.4)	
Race/ethnicity				
Non-Hispanic white	72.3 (70.1–74.4)	71.2 (68.8–73.6)	66.2 (63.6–68.6)	
Non-Hispanic black	8.5 (7.5–9.6)	9.3 (8.3–10.4)	14.8 (13.2–16.6)	
Mexican American	5.5 (4.8–6.3)	8.4 (7.2–9.7)	9.1 (7.9–10.5)	
Other Hispanic	4.2 (3.5–5.0)	5.6 (4.7–6.6)	5.3 (4.5–6.3)	
Other or mixed race	9.6 (8.5–10.8)	5.5 (4.8–6.4)	4.5 (4.0–5.2)	
Education level				
<High school	13.8 (12.4–15.4)	15.6 (14.4–16.9)	16.6 (15.6–17.7)	
High school graduate or GED	21.0 (19.7–22.5)	24.3 (22.9–25.7)	26.0 (24.8–27.2)	
Some college	29.6 (28.0–31.3)	29.5 (28.1–30.9)	34.9 (33.6–36.2)	
≥College	35.5 (33.3–37.8)	30.7 (28.7–32.7)	22.5 (21.0–24.1)	
FIPR ^b^				
<1.30	20.1 (18.7–21.6)	18.7 (17.5–20.0)	22.6 (21.2–24.1)	
1.30–1.84	10.0 (9.1–10.9)	9.8 (9.0–10.6)	11.7 (10.9–12.5)	
1.85–2.99	17.2 (16.1–18.4)	18.2 (17.1–19.4)	18.9 (17.8–20.1)	
≥3.00	52.7 (50.5–55.0)	53.3 (51.4–55.2)	46.7 (44.8–48.7)	

Abbreviations: GED, general equivalency diploma. FIPR: Family income to poverty ratio. ^a^ Normal weight is defined as a body mass index (BMI) ranging from 18.5 to 24.9, overweight is defined as a BMI ranging from 25 to 29.9, and obesity is defined as a BMI ≥ 30. ^b^ Family income to poverty ratio represents the ratio of family income to the federal poverty threshold, adjusting for household size.

**Table 2:** Trends in components of Healthy Eating Index (HEI)-2020 diet score among US adults by bodyweight status, 1999–2020 ^a^.

Dietary Score	HEI-2020 Scoring Standards (Range)	Survey-Weighted Mean Score (95% CI)										*p* Value for Trend	Change from 1999 to 2020, Mean (95% CI)
		1999–2000 (*n* = 1289)	2001–2002 (*n* = 2774)	2003–2004 (*n* = 3053)	2005–2006 (*n* = 3204)	2007–2008 (*n* = 3731)	2009–2010 (*n* = 4012)	2011–2012 (*n* = 3382)	2013–2014 (*n* = 3876)	2015–2016 (*n* = 3786)	2017–2020 (*n* = 5522)		
HEI-2020 score (0–100 points)													
Total population		45.4 (43.6–47.1)	48.9 (48.2–49.7)	49.2 (48.2–50.2)	50.1 (49.2–51.0)	50.4 (49.2–51.5)	51.2 (50.6–51.8)	52.0 (51.2–52.8)	51.2 (50.5–51.8)	50.6 (49.3–51.9)	50.1 (49.2–51.0)	<0.001	4.67 (2.70 to 6.64)
Normal weight		46.6 (44.4–48.9)	50.2 (48.9–51.6)	49.6 (47.9–51.4)	50.8 (49.5–52.2)	51.1 (49.4–52.8)	52.6 (51.7–53.4)	54.1 (52.7–55.4)	53.1 (51.6–54.6)	52.5 (50.8–54.2)	51.6 (49.7–53.5)	0.010	4.97 (2.02 to 7.93)
Overweight		45.6 (43.5–47.6)	49.1 (48.0–50.3)	50.0 (48.8–51.2)	51.1 (50.2–52.0)	50.4 (48.9–52.0)	51.9 (51.1–52.8)	52.7 (51.7–53.7)	51.9 (50.4–53.5)	50.7 (48.9–52.5)	51.1 (50.2–52.1)	<0.001	5.58 (3.31 to 7.85)
Obesity		43.8 (42.0–45.7)	47.3 (46.1–48.4)	47.9 (47.2–48.6)	48.5 (47.6–49.3)	49.7 (48.6–50.8)	49.6 (48.7–50.5)	49.5 (48.7–50.4)	49.1 (48.6–49.6)	49.4 (48.2–50.6)	48.4 (47.5–49.3)	<0.001	4.55 (2.47 to 6.63)
*p* for interaction												<0.001 ^b^	<0.001 ^c^
Adequacy:													
Total fruits (5 of 100 points)	0 to ≥0.8 cup equivalent per 1000 kcal												
Total population		2.0 (1.7–2.3)	2.1 (2.0–2.2)	2.1 (1.9–2.3)	2.2 (2.1–2.3)	2.2 (2.0–2.3)	2.3 (2.2–2.3)	2.2 (2.1–2.3)	2.1 (2.0–2.2)	2.0 (1.8–2.2)	1.9 (1.8–2.1)	<0.001	−0.05 (−0.38 to 0.29)
Normal weight		2.2 (1.7–2.6)	2.4 (2.1–2.7)	2.1 (1.9–2.4)	2.3 (2.1–2.4)	2.2 (2.0–2.4)	2.4 (2.3–2.6)	2.5 (2.3–2.6)	2.2 (2.1–2.4)	2.2 (2.0–2.4)	2.0 (1.8–2.2)	0.009	−0.16 (−0.67 to 0.34)
Overweight		1.9 (1.7–2.1)	2.1 (2.0–2.3)	2.3 (2.1–2.4)	2.3 (2.1–2.4)	2.2 (2.1–2.4)	2.4 (2.2–2.5)	2.3 (2.1–2.5)	2.2 (2.0–2.3)	2.0 (1.8–2.3)	2.1 (1.9–2.2)	0.009	0.18 (−0.08 to 0.43)
Obesity		1.9 (1.5–2.3)	1.9 (1.7–2.1)	2.0 (1.7–2.2)	2.1 (1.9–2.2)	2.0 (1.8–2.2)	2.1 (2.0–2.2)	1.9 (1.8–2.0)	1.9 (1.8–2.0)	1.9 (1.7–2.1)	1.8 (1.7–2.0)	0.043	−0.08 (−0.50 to 0.34)
*p* for interaction												<0.001 ^b^	0.018 ^c^
Whole fruits (5 of 100 points)	0 to ≥0.4 cup equivalent per 1000 kcal												
Total population		1.7 (1.5–1.9)	1.9 (1.8–2.0)	1.9 (1.8–2.1)	2.1 (1.9–2.2)	2.1 (2.0–2.2)	2.2 (2.2–2.3)	2.2 (2.1–2.4)	2.2 (2.1–2.2)	2.1 (1.9–2.3)	2.0 (1.9–2.2)	0.041	0.33 (0.05 to 0.61)
Normal weight		1.8 (1.5–2.1)	2.1 (1.9–2.3)	2.0 (1.7–2.2)	2.1 (1.9–2.3)	2.2 (2.0–2.4)	2.4 (2.2–2.6)	2.4 (2.1–2.6)	2.3 (2.1–2.5)	2.3 (2.0–2.5)	2.1 (1.9–2.3)	0.416	0.28 (−0.12 to 0.68)
Overweight		1.5 (1.3–1.8)	1.9 (1.8–2.1)	2.1 (1.9–2.3)	2.2 (2.0–2.4)	2.2 (2.0–2.4)	2.4 (2.2–2.5)	2.3 (2.1–2.6)	2.3 (2.1–2.5)	2.1 (1.9–2.3)	2.2 (2.1–2.3)	<0.001	0.68 (0.41 to 0.94)
Obesity		1.7 (1.3–2.1)	1.7 (1.5–1.9)	1.7 (1.5–1.9)	2.0 (1.8–2.2)	2.0 (1.8–2.1)	2.0 (1.9–2.2)	1.9 (1.7–2.1)	1.9 (1.8–2.1)	1.9 (1.7–2.1)	1.9 (1.7–2.1)	1.000	0.12 (−0.33 to 0.57)
*p* for interaction												<0.001 ^b^	0.013 ^c^
Total vegetables (5 of 100 points)	0 to ≥1.1 cup equivalent per 1000 kcal												
Total population		3.6 (3.4–3.7)	3.1 (3.0–3.1)	3.2 (3.1–3.3)	3.2 (3.1–3.3)	3.2 (3.1–3.2)	3.1 (3.1–3.2)	3.1 (3.1–3.2)	3.1 (3.0–3.2)	3.1 (3.0–3.2)	3.0 (2.9–3.0)	<0.001	−0.60 (−0.77 to −0.43)
Normal weight		3.5 (3.3–3.6)	3.1 (3.0–3.3)	3.1 (3.0–3.3)	3.2 (3.1–3.3)	3.2 (3.1–3.4)	3.2 (3.0–3.3)	3.2 (3.0–3.3)	3.0 (2.8–3.2)	3.2 (3.1–3.3)	3.0 (2.8–3.2)	<0.001	−0.49 (−0.72 to −0.27)
Overweight		3.5 (3.3–3.8)	3.0 (2.9–3.1)	3.3 (3.1–3.4)	3.1 (3.0–3.2)	3.1 (3.0–3.2)	3.1 (3.0–3.2)	3.2 (3.1–3.3)	3.1 (3.0–3.3)	3.0 (2.8–3.2)	3.0 (2.9–3.2)	0.014	−0.51 (−0.83 to −0.20)
Obesity		3.6 (3.4–3.9)	3.1 (2.9–3.2)	3.1 (3.0–3.2)	3.2 (3.1–3.3)	3.1 (3.0–3.3)	3.1 (3.0–3.1)	3.0 (2.9–3.2)	3.0 (2.9–3.1)	3.0 (2.9–3.2)	2.9 (2.8–3.0)	<0.001	−0.76 (−1.01 to −0.51)
*p* for interaction												<0.001 ^b^	<0.001 ^c^
Greens and Beans (5 of 100 points)	0 to ≥0.2 cup equivalent per 1000 kcal												
Total population		1.0 (0.9–1.2)	1.3 (1.1–1.4)	1.3 (1.2–1.4)	1.4 (1.2–1.5)	1.4 (1.2–1.5)	1.4 (1.3–1.5)	1.5 (1.4–1.6)	1.6 (1.5–1.7)	1.6 (1.5–1.8)	1.5 (1.4–1.6)	<0.001	0.44 (0.24 to 0.64)
Normal weight		1.2 (0.9–1.6)	1.3 (1.1–1.5)	1.4 (1.2–1.5)	1.5 (1.3–1.7)	1.5 (1.3–1.6)	1.6 (1.4–1.7)	1.7 (1.6–1.8)	1.7 (1.6–1.9)	1.9 (1.6–2.1)	1.6 (1.4–1.8)	0.283	0.38 (0.00 to 0.77)
Overweight		1.1 (0.9–1.3)	1.3 (1.1–1.4)	1.4 (1.2–1.6)	1.3 (1.2–1.5)	1.3 (1.1–1.5)	1.4 (1.2–1.5)	1.6 (1.4–1.8)	1.6 (1.4–1.8)	1.6 (1.4–1.8)	1.6 (1.4–1.8)	0.008	0.49 (0.21 to 0.77)
Obesity		0.8 (0.6–0.9)	1.2 (1.0–1.4)	1.2 (1.1–1.3)	1.3 (1.2–1.5)	1.3 (1.2–1.4)	1.3 (1.2–1.4)	1.3 (1.2–1.4)	1.5 (1.3–1.6)	1.5 (1.4–1.7)	1.3 (1.2–1.4)	<0.001	0.55 (0.37 to 0.74)
*p* for interaction												<0.001 ^b^	<0.001 ^c^
Whole grains (10 of 100 points)	0 to ≥1.5 oz equivalent per 1000 kcal												
Total population		1.5 (1.3–1.7)	2.1 (2.0–2.3)	2.0 (1.8–2.2)	2.3 (2.1–2.5)	2.3 (2.1–2.5)	2.6 (2.5–2.7)	2.8 (2.6–3.0)	2.7 (2.6–2.9)	2.7 (2.5–3.0)	2.5 (2.3–2.6)	<0.001	0.99 (0.71 to 1.27)
Normal weight		1.5 (1.2–1.8)	2.2 (1.9–2.4)	2.1 (1.8–2.3)	2.3 (2.0–2.5)	2.3 (2.1–2.5)	2.8 (2.6–3.1)	3.2 (2.9–3.6)	3.1 (2.8–3.3)	2.8 (2.5–3.2)	2.8 (2.5–3.2)	<0.001	1.34 (0.82 to 1.86)
Overweight		1.7 (1.3–2.0)	2.1 (1.9–2.4)	2.0 (1.8–2.2)	2.5 (2.3–2.8)	2.3 (2.1–2.6)	2.6 (2.4–2.8)	2.9 (2.6–3.1)	2.8 (2.5–3.1)	2.7 (2.4–3.1)	2.4 (2.2–2.7)	0.007	0.79 (0.33 to 1.24)
Obesity		1.3 (1.1–1.4)	2.0 (1.7–2.4)	1.9 (1.7–2.1)	2.2 (2.0–2.3)	2.2 (2.0–2.4)	2.4 (2.2–2.6)	2.5 (2.2–2.7)	2.4 (2.2–2.6)	2.7 (2.5–2.9)	2.3 (2.1–2.4)	<0.001	1.01 (0.78 to 1.23)
*p* for interaction												<0.001 ^b^	<0.001 ^c^
Dairy (10 of 100 points)	0 to ≥1.3 cup equivalent per 1000 kcal												
Total population		5.7 (5.4–6.0)	4.8 (4.6–5.0)	4.8 (4.5–5.0)	5.1 (5.0–5.3)	5.1 (4.8–5.3)	5.4 (5.3–5.5)	5.3 (5.1–5.4)	5.2 (5.0–5.3)	5.0 (4.8–5.3)	4.7 (4.6–4.9)	<0.001	−0.98 (−1.32 to −0.64)
Normal weight		5.9 (5.4–6.3)	5.2 (4.7–5.7)	5.1 (4.7–5.4)	5.2 (4.9–5.4)	5.2 (4.9–5.6)	5.4 (5.2–5.6)	5.4 (5.1–5.6)	5.3 (5.0–5.6)	5.2 (5.0–5.4)	4.5 (4.3–4.7)	<0.001	−1.32 (−1.81 to −0.83)
Overweight		5.4 (5.2–5.7)	4.8 (4.5–5.1)	4.6 (4.3–4.9)	5.3 (5.0–5.6)	5.1 (4.8–5.3)	5.4 (5.2–5.6)	5.2 (5.0–5.5)	5.2 (5.0–5.4)	5.1 (4.7–5.6)	4.8 (4.6–5.0)	<0.001	−0.64 (−0.99 to −0.29)
Obesity		5.9 (5.2–6.6)	4.4 (4.2–4.7)	4.6 (4.4–4.9)	5.0 (4.8–5.2)	5.0 (4.6–5.3)	5.3 (5.1–5.4)	5.2 (4.8–5.5)	5.0 (4.7–5.3)	4.8 (4.6–5.0)	4.8 (4.6–5.1)	0.017	−1.05 (−1.77 to −0.32)
*p* for interaction												<0.001 ^b^	0.001 ^c^
Total protein foods (5 of 100 points)	0 to ≥2.5 oz equivalent per 1000 kcal												
Total population		4.6 (4.5–4.6)	4.2 (4.2–4.2)	4.2 (4.1–4.2)	4.3 (4.2–4.3)	4.3 (4.2–4.3)	4.3 (4.2–4.3)	4.2 (4.1–4.3)	4.3 (4.2–4.3)	4.3 (4.2–4.3)	4.3 (4.2–4.3)	<0.001	−0.30 (−0.37 to −0.23)
Normal weight		4.4 (4.3–4.6)	4.2 (4.1–4.3)	4.1 (4.0–4.2)	4.2 (4.1–4.3)	4.2 (4.1–4.3)	4.2 (4.1–4.3)	4.1 (4.0–4.2)	4.1 (4.0–4.2)	4.2 (4.1–4.3)	4.3 (4.2–4.4)	0.051	−0.16 (−0.34 to 0.01)
Overweight		4.7 (4.6–4.7)	4.2 (4.1–4.3)	4.2 (4.1–4.3)	4.3 (4.2–4.3)	4.3 (4.2–4.4)	4.3 (4.2–4.3)	4.3 (4.2–4.4)	4.3 (4.2–4.4)	4.3 (4.2–4.3)	4.3 (4.2–4.4)	<0.001	−0.40 (−0.52 to −0.27)
Obesity		4.6 (4.5–4.7)	4.2 (4.1–4.3)	4.3 (4.2–4.4)	4.3 (4.3–4.4)	4.4 (4.3–4.5)	4.4 (4.3–4.4)	4.3 (4.2–4.4)	4.3 (4.3–4.4)	4.3 (4.3–4.4)	4.3 (4.2–4.3)	<0.001	−0.36 (−0.47 to −0.25)
*p* for interaction												<0.001 ^b^	<0.001 ^c^
Seafood and plant proteins (5 of 100 points)	0 to ≥0.8 oz equivalent per 1000 kcal												
Total population		1.8 (1.7–2.0)	1.9 (1.8–2.0)	2.1 (2.0–2.2)	2.1 (2.0–2.3)	2.2 (2.0–2.3)	2.2 (2.1–2.3)	2.3 (2.2–2.4)	2.3 (2.2–2.4)	2.4 (2.2–2.6)	2.3 (2.2–2.4)	<0.001	0.50 (0.28 to 0.71)
Normal weight		2.1 (1.8–2.4)	1.9 (1.7–2.1)	2.1 (2.0–2.2)	2.3 (2.1–2.4)	2.4 (2.1–2.6)	2.4 (2.2–2.5)	2.5 (2.3–2.6)	2.6 (2.4–2.8)	2.7 (2.5–2.9)	2.5 (2.3–2.7)	0.001	0.44 (0.04 to 0.83)
Overweight		1.5 (1.4–1.7)	2.0 (1.8–2.1)	2.1 (2.0–2.3)	2.2 (2.1–2.3)	2.2 (1.9–2.4)	2.3 (2.1–2.4)	2.4 (2.3–2.5)	2.3 (2.1–2.5)	2.4 (2.2–2.6)	2.5 (2.3–2.7)	<0.001	0.94 (0.65 to 1.22)
Obesity		1.9 (1.6–2.2)	1.8 (1.7–2.0)	2.0 (1.8–2.2)	2.0 (1.8–2.1)	2.0 (1.8–2.2)	2.1 (1.9–2.2)	2.0 (1.9–2.2)	2.1 (2.0–2.2)	2.2 (2.1–2.4)	2.1 (2.0–2.2)	0.018	0.25 (−0.06 to 0.57)
*p* for interaction												<0.001 ^b^	<0.001 ^c^
Fatty acids (10 of 100 points)	≤1.2 to ≥2.5												
Total population		4.3 (4.1–4.5)	4.8 (4.6–4.9)	5.0 (4.9–5.1)	4.6 (4.5–4.8)	4.8 (4.6–5.0)	4.9 (4.8–5.0)	5.1 (5.0–5.3)	4.9 (4.8–5.1)	4.8 (4.6–5.0)	4.9 (4.7–5.0)	<0.001	0.58 (0.31 to 0.84)
Normal weight		4.3 (3.7–4.8)	4.6 (4.3–4.9)	5.1 (4.8–5.4)	4.6 (4.5–4.8)	4.6 (4.3–4.9)	4.9 (4.6–5.1)	5.3 (5.0–5.6)	5.0 (4.8–5.2)	4.8 (4.5–5.1)	5.1 (4.7–5.4)	0.159	0.80 (0.14 to 1.46)
Overweight		4.4 (3.9–4.9)	4.7 (4.5–4.9)	5.1 (4.9–5.4)	4.7 (4.4–5.0)	4.8 (4.6–5.1)	5.0 (4.8–5.2)	5.0 (4.8–5.2)	4.9 (4.6–5.1)	4.7 (4.3–5.1)	5.0 (4.7–5.2)	0.271	0.62 (0.06 to 1.17)
Obesity		4.2 (3.8–4.7)	5.0 (4.8–5.2)	4.9 (4.6–5.1)	4.6 (4.4–4.8)	4.9 (4.6–5.1)	4.8 (4.6–5.0)	5.1 (4.9–5.4)	4.9 (4.8–5.1)	4.9 (4.7–5.2)	4.7 (4.5–4.9)	0.043	0.43 (−0.10 to 0.95)
*p* for interaction												<0.001 ^b^	0.322 ^c^
Moderation:													
Refined grains (10 of 100 points)	≤1.8 to ≥4.3 oz equivalent per 1000 kcal												
Total population		3.6 (3.2–4.0)	5.4 (4.9–5.9)	5.8 (5.6–6.1)	5.8 (5.5–6.0)	6.1 (5.8–6.5)	5.8 (5.6–6.1)	5.7 (5.5–5.9)	5.8 (5.6–6.1)	6.1 (5.8–6.5)	6.1 (5.9–6.3)	<0.001	2.51 (2.04 to 2.97)
Normal weight		3.8 (3.1–4.5)	6.0 (5.7–6.3)	5.6 (5.3–5.9)	6.2 (6.0–6.5)	6.1 (5.8–6.4)	6.2 (5.9–6.5)	6.2 (5.8–6.5)	6.2 (6.0–6.4)	6.5 (6.1–6.9)	6.2 (5.9–6.5)	<0.001	2.41 (1.66 to 3.16)
Overweight		3.9 (3.4–4.4)	5.7 (5.5–6.0)	5.7 (5.4–5.9)	6.2 (6.0–6.4)	6.0 (5.7–6.3)	6.1 (5.9–6.4)	6.2 (5.9–6.5)	6.3 (6.0–6.6)	6.1 (5.8–6.5)	6.2 (5.9–6.4)	<0.001	2.27 (1.68 to 2.86)
Obesity		3.6 (3.2–4.0)	5.4 (4.9–5.9)	5.8 (5.6–6.1)	5.8 (5.5–6.0)	6.1 (5.8–6.5)	5.8 (5.6–6.1)	5.7 (5.5–5.9)	5.8 (5.6–6.1)	6.1 (5.8–6.5)	6.1 (5.9–6.3)	<0.001	2.51 (2.04 to 2.97)
*p* for interaction												<0.001 ^b^	<0.001 ^c^
Sodium (10 of 100 points)	≤1.1 to ≥2.0 g per 1000 kcal												
Total population		5.3 (4.8–5.7)	5.3 (5.1–5.6)	4.8 (4.6–5.1)	4.4 (4.3–4.6)	4.4 (4.4–4.5)	3.9 (3.7–4.1)	4.2 (4.1–4.2)	4.1 (3.9–4.3)	4.0 (3.8–4.2)	4.5 (4.3–4.7)	<0.001	−0.76 (−1.22 to −0.30)
Normal weight		5.6 (5.0–6.3)	5.5 (5.1–5.8)	4.9 (4.6–5.1)	4.7 (4.4–5.0)	4.6 (4.3–4.9)	4.2 (3.9–4.6)	4.6 (4.3–4.9)	4.5 (4.1–4.9)	4.1 (3.7–4.5)	4.8 (4.5–5.1)	0.039	−0.83 (−1.57 to −0.10)
Overweight		5.2 (4.7–5.8)	5.4 (5.1–5.7)	4.9 (4.6–5.2)	4.7 (4.5–4.9)	4.5 (4.4–4.7)	4.0 (3.8–4.2)	4.0 (3.8–4.3)	4.0 (3.8–4.2)	4.1 (3.9–4.3)	4.6 (4.3–4.9)	0.001	−0.65 (−1.27 to −0.02)
Obesity		4.9 (4.4–5.3)	5.0 (4.7–5.4)	4.8 (4.5–5.1)	3.9 (3.7–4.1)	4.2 (4.0–4.4)	3.6 (3.5–3.7)	3.9 (3.7–4.1)	3.9 (3.6–4.2)	3.8 (3.6–4.1)	4.3 (4.0–4.5)	<0.001	−0.60 (−1.12 to −0.07)
*p* for interaction												<0.001 ^b^	0.002 ^c^
Added sugars (10 of 100 points)	≤6.5% to ≥26% of energy												
Total population		4.2 (3.8–4.5)	5.4 (5.1–5.7)	6.1 (5.7–6.4)	6.5 (6.3–6.8)	6.4 (6.1–6.7)	6.6 (6.5–6.8)	6.7 (6.5–6.9)	6.7 (6.5–6.9)	6.9 (6.7–7.1)	7.0 (6.8–7.2)	<0.001	2.86 (2.49 to 3.22)
Normal weight		4.1 (3.5–4.7)	5.4 (4.9–6.0)	5.9 (5.5–6.3)	6.4 (6.0–6.8)	6.4 (6.0–6.7)	6.4 (6.1–6.7)	6.5 (6.2–6.8)	6.5 (6.0–6.9)	7.0 (6.6–7.3)	7.1 (6.8–7.4)	<0.001	2.98 (2.31 to 3.64)
Overweight		4.5 (4.1–4.8)	5.5 (5.2–5.9)	6.2 (5.9–6.6)	6.5 (6.2–6.7)	6.2 (5.8–6.6)	6.7 (6.6–6.9)	7.0 (6.6–7.3)	7.1 (6.8–7.5)	7.0 (6.6–7.4)	7.2 (7.0–7.4)	<0.001	2.69 (2.28 to 3.11)
Obesity		3.9 (3.4–4.4)	5.3 (5.0–5.6)	6.1 (5.6–6.5)	6.6 (6.4–6.9)	6.6 (6.3–6.9)	6.7 (6.5–6.9)	6.6 (6.3–6.9)	6.4 (6.2–6.7)	6.8 (6.6–7.1)	6.9 (6.6–7.1)	<0.001	2.95 (2.38 to 3.51)
*p* for interaction												<0.001 ^b^	<0.001 ^c^
Saturated fats (10 of 100 points)	≤8% to ≥16% of energy												
Total population		6.0 (5.7–6.4)	6.3 (6.1–6.4)	6.0 (5.8–6.2)	5.8 (5.6–6.0)	6.0 (5.8–6.2)	6.2 (6.1–6.4)	6.3 (6.1–6.5)	6.0 (5.8–6.2)	5.5 (5.3–5.7)	5.3 (5.1–5.5)	<0.001	−0.71 (−1.09 to −0.34)
Normal weight		6.3 (5.8–6.7)	6.3 (5.9–6.7)	6.3 (6.0–6.6)	5.9 (5.7–6.1)	6.2 (6.0–6.4)	6.5 (6.3–6.6)	6.6 (6.4–6.8)	6.5 (6.3–6.8)	5.6 (5.4–5.9)	5.6 (5.4–5.9)	<0.001	−0.68 (−1.19 to −0.17)
Overweight		6.2 (5.8–6.7)	6.3 (6.2–6.5)	6.1 (5.9–6.3)	5.9 (5.7–6.2)	6.1 (5.9–6.4)	6.2 (6.0–6.4)	6.3 (6.1–6.5)	5.8 (5.5–6.1)	5.5 (5.3–5.8)	5.4 (5.0–5.7)	<0.001	−0.87 (−1.42 to −0.31)
Obesity		5.5 (5.0–6.0)	6.2 (5.9–6.5)	5.6 (5.3–5.8)	5.6 (5.3–5.8)	5.7 (5.4–6.0)	6.0 (5.7–6.3)	6.1 (5.7–6.4)	5.8 (5.6–6.1)	5.3 (5.1–5.5)	5.1 (4.9–5.3)	<0.001	−0.41 (−0.96 to 0.14)
*p* for interaction												<0.001 ^b^	<0.001 ^c^

Abbreviations: HEI, Healthy Eating Index. ^a^ Normal weight is defined as a body mass index (BMI) ranging from 18.5 to 24.9, overweight is defined as a BMI ranging from 25 to 29.9, and obesity is defined as a BMI ≥ 30. ^b^ *p* for interaction assessing potential heterogeneous trends in components of HEI-2020 diet score by bodyweight status. ^c^ *p* for interaction assessing potential heterogeneous changes in components of HEI-2020 diet score from 1999 to 2000 to 2017 to 2020 by bodyweight status.

**Table 3:** Mean intake of key food groups and nutrients among US adults by bodyweight status, 2017–2020 ^a,b^.

Dietary Intake	Survey-Weighted Mean Intake (95% CI)			*p* Value
	Normal Weight (*n* = 1316)	Overweight (*n* = 1752)	Obesity (*n* = 2454)	
Key Food Groups				
HEI-2020 score	51.61 (49.74–53.48)	51.14 (50.20–52.08)	48.38 (47.48–49.27)	<0.001
Total fruits, servings/d	0.46 (0.40–0.51)	0.47 (0.43–0.50)	0.44 (0.40–0.47)	0.232
Whole fruits, servings/d	0.37 (0.32–0.41)	0.37 (0.34–0.40)	0.33 (0.30–0.36)	0.105
Total vegetables, servings/d	0.86 (0.79–0.93)	0.85 (0.80–0.89)	0.78 (0.74–0.82)	0.039
Greens and Beans, servings/d	0.16 (0.14–0.19)	0.15 (0.13–0.17)	0.11 (0.10–0.12)	0.002
Whole grains, servings/d	0.51 (0.43–0.58)	0.43 (0.38–0.48)	0.39 (0.36–0.43)	0.005
Dairy, servings/d	0.64 (0.60–0.69)	0.68 (0.64–0.72)	0.70 (0.65–0.75)	0.267
Total protein foods, servings/d	3.26 (3.07–3.44)	3.33 (3.17–3.48)	3.25 (3.14–3.36)	0.737
Seafood and plant proteins, servings/d	1.00 (0.85–1.15)	0.98 (0.88–1.08)	0.79 (0.73–0.85)	0.001
Fatty acids	1.95 (1.89–2.02)	1.95 (1.89–2.00)	1.88 (1.84–1.92)	0.164
Refined grains, servings/d	2.64 (2.50–2.77)	2.66 (2.56–2.75)	2.69 (2.62–2.77)	0.864
Sodium, g/d	1.63 (1.58–1.68)	1.64 (1.60–1.68)	1.70 (1.67–1.73)	0.005
Added sugars, tsp equivalents/d, servings/d	11.79 (11.04–12.54)	11.71 (11.03–12.40)	12.33 (11.59–13.07)	0.312
Saturated fats, servings/d	11.40 (11.14–11.66)	11.68 (11.31–12.04)	11.98 (11.76–12.20)	0.005
Nutrients				
Energy, kcal	1982.36 (1926.20–2038.53)	2018.42 (1960.60–2076.24)	2036.44 (1984.15–2088.73)	0.353
Total carbohydrate, % of energy	46.78 (45.89–47.67)	46.28 (45.39–47.18)	45.82 (45.23–46.42)	0.124
Protein, % of energy	15.41 (15.00–15.83)	15.69 (15.37–16.02)	15.76 (15.46–16.05)	0.331
Total fat, % of energy	35.75 (35.05–36.44)	36.42 (35.72–37.12)	36.64 (36.20–37.07)	0.093
Saturated fats, % of energy	11.54 (11.25–11.83)	11.76 (11.42–12.11)	12.06 (11.87–12.26)	0.014
Monounsaturated fat, % of energy	12.23 (11.90–12.56)	12.57 (12.30–12.85)	12.43 (12.24–12.62)	0.586
Polyunsaturated fat, % of energy	8.42 (8.17–8.67)	8.44 (8.29–8.59)	8.42 (8.25–8.60)	1.000
Polyunsaturated: saturated fat ratio	0.77 (0.74–0.80)	0.77 (0.74–0.79)	0.74 (0.72–0.76)	0.147
Cholesterol, mg/d	279.86 (267.45–292.26)	297.74 (279.89–315.59)	302.82 (292.89–312.76)	0.005
Dietary fiber, g/d	16.21 (15.32–17.10)	16.26 (15.56–16.97)	14.84 (14.30–15.39)	<0.001
Potassium, mg/d	2478.38 (2380.12–2576.65)	2529.00 (2446.71–2611.28)	2416.19 (2332.41–2499.98)	0.053
Magnesium, mg/d	287.62 (276.39–298.85)	291.56 (281.42–301.71)	277.09 (268.81–285.37)	0.020
Calcium, mg/d	876.19 (848.16–904.22)	896.31 (873.96–918.66)	916.56 (873.36–959.76)	0.289

^a^ Normal weight is defined as a body mass index (BMI) ranging from 18.5 to 24.9, overweight is defined as a BMI ranging from 25 to 29.9, and obesity is defined as a BMI ≥ 30. ^b^ Means for total fat, saturated fat, monounsaturated fat, polyunsaturated fat, protein, and carbohydrate were adjusted as a percentage of total energy.

**Table 4:** Trends in estimated percentage of energy intake from total carbohydrate, protein, and total fat among US adults by bodyweight status, 1999–2020 ^a^.

Energy Intake	Survey-Weighted Mean Score (95% CI)										*p* for Trend	Change from 1999 to 2020, Mean (95% CI)
	1999–2000 (*n* = 1289)	2001–2002 (*n* = 2774)	2003–2004 (*n* = 3053)	2005–2006 (*n* = 3204)	2007–2008 (*n* = 3731)	2009–2010 (*n* = 4012)	2011–2012 (*n* = 3382)	2013–2014 (*n* = 3876)	2015–2016 (*n* = 3786)	2017–2020 (*n* = 5522)		
Total energy intake, kcal												
Total population	1856.6 (1769.6–1943.5)	2012.4 (1970.2–2054.7)	2019.0 (1972.3–2065.7)	2059.7 (2009.4–2110.1)	2012.8 (1960.3–2065.4)	2031.9 (2000.9–2063.0)	2075.8 (2037.4–2114.2)	2035.5 (2006.7–2064.2)	2038.7 (2004.5–2072.8)	2017.4 (1985.8–2049.0)	0.007	160.88 (68.35 to 253.40)
Normal weight	1911.6 (1793.9–2029.4)	2022.4 (1924.1–2120.7)	1987.9 (1920.5–2055.3)	2063.9 (1991.0–2136.8)	2030.7 (1954.1–2107.2)	1978.1 (1928.2–2028.1)	2085.6 (2039.0–2132.2)	2030.5 (1944.4–2116.6)	2006.2 (1962.4–2050.1)	1982.4 (1926.2–2038.5)	0.053	70.73 (−59.74 to 201.21)
Overweight	1844.9 (1685.0–2004.9)	2077.6 (2012.3–2143.0)	2007.6 (1964.2–2051.1)	2107.1 (2042.5–2171.6)	1999.1 (1949.8–2048.3)	2111.3 (2039.5–2183.2)	2068.1 (1998.0–2138.1)	2064.9 (2008.0–2121.8)	2064.2 (2002.6–2125.9)	2018.4 (1960.6–2076.2)	0.406	173.49 (3.41 to 343.58)
Obesity	1807.2 (1674.1–1940.3)	1924.4 (1852.9–1995.9)	2062.6 (1981.1–2144.1)	2010.8 (1950.5–2071.2)	2011.3 (1933.4–2089.1)	2003.4 (1953.5–2053.3)	2074.6 (2004.3–2144.9)	2013.4 (1965.6–2061.1)	2039.0 (1978.2–2099.8)	2036.4 (1984.2–2088.7)	0.017	229.25 (86.24 to 372.26)
*p* for interaction											0.008 ^b^	0.015 ^c^
Total Carbohydrate, % of Energy												
Total population	51.1 (50.2–51.9)	50.8 (50.1–51.5)	49.3 (48.5–50.1)	48.7 (48.1–49.3)	49.1 (48.6–49.7)	49.1 (48.7–49.6)	49.4 (48.8–50.0)	48.0 (47.4–48.6)	46.9 (46.4–47.4)	46.2 (45.7–46.7)	<0.001	−4.86 (−5.86 to −3.86)
Normal weight	52.4 (51.5–53.3)	51.0 (49.8–52.2)	50.5 (49.5–51.5)	49.0 (48.2–49.7)	49.8 (49.0–50.6)	50.3 (49.7–51.0)	50.7 (49.9–51.6)	49.8 (48.9–50.6)	47.1 (46.4–47.8)	46.8 (45.9–47.7)	<0.001	−5.62 (−6.86 to −4.38)
Overweight	50.4 (49.0–51.7)	50.3 (49.5–51.1)	49.3 (48.4–50.2)	48.8 (48.0–49.6)	49.6 (48.9–50.2)	48.7 (48.0–49.3)	48.7 (48.1–49.3)	46.1 (45.2–47.0)	47.2 (46.0–48.3)	46.3 (45.4–47.2)	<0.001	−4.07 (−5.69 to −2.45)
Obesity	50.3 (49.1–51.5)	51.1 (50.1–52.1)	48.0 (46.8–49.2)	48.4 (47.5–49.4)	48.1 (47.2–49.1)	48.7 (47.7–49.6)	48.8 (47.8–49.7)	48.3 (47.6–49.0)	46.6 (45.9–47.3)	45.8 (45.2–46.4)	<0.001	−4.48 (−5.80 to −3.16)
*p* for interaction											<0.001 ^b^	<0.001 ^c^
Protein, % of Energy												
Total population	15.0 (14.8–15.3)	15.1 (14.9–15.3)	15.5 (15.2–15.8)	16.0 (15.8–16.2)	15.9 (15.7–16.1)	16.1 (15.7–16.4)	15.7 (15.4–15.9)	16.0 (15.8–16.3)	15.8 (15.5–16.1)	15.7 (15.4–15.9)	0.001	0.64 (0.32 to 0.96)
Normal weight	14.3 (13.9–14.8)	15.2 (14.9–15.5)	15.0 (14.6–15.3)	15.7 (15.4–16.0)	15.6 (15.1–16.0)	15.7 (15.3–16.1)	15.1 (14.8–15.5)	15.6 (15.1–16.1)	15.8 (15.5–16.2)	15.4 (15.0–15.8)	0.004	1.10 (0.49 to 1.70)
Overweight	15.5 (14.9–16.2)	15.1 (14.8–15.4)	15.7 (15.3–16.1)	15.9 (15.7–16.1)	15.8 (15.4–16.2)	16.1 (15.6–16.5)	15.9 (15.6–16.2)	16.4 (16.0–16.7)	15.8 (15.4–16.2)	15.7 (15.4–16.0)	0.054	0.15 (−0.60 to 0.90)
Obesity	15.2 (14.7–15.8)	14.9 (14.6–15.2)	15.8 (15.4–16.1)	16.4 (16.0–16.8)	16.3 (16.0–16.6)	16.4 (16.0–16.8)	15.9 (15.5–16.3)	16.0 (15.7–16.4)	15.8 (15.5–16.2)	15.8 (15.5–16.1)	0.001	0.51 (−0.08 to 1.09)
*p* for interaction											<0.001 ^b^	<0.001 ^c^
Total fat, % of Energy												
Total population	32.1 (31.3–32.8)	33.5 (33.0–33.9)	33.9 (33.2–34.5)	33.8 (33.4–34.3)	33.7 (33.4–34.1)	33.2 (32.7–33.7)	33.2 (32.7–33.6)	34.1 (33.7–34.4)	35.5 (35.1–35.9)	36.3 (36.0–36.7)	<0.001	4.27 (3.41 to 5.12)
Normal weight	31.1 (30.2–32.0)	32.8 (32.0–33.5)	33.0 (32.2–33.7)	33.3 (32.7–33.9)	33.0 (32.4–33.5)	32.5 (32.0–33.1)	32.2 (31.7–32.7)	32.5 (31.9–33.0)	35.0 (34.2–35.8)	35.7 (35.1–36.4)	<0.001	4.66 (3.52 to 5.80)
Overweight	31.8 (31.1–32.6)	33.5 (32.9–34.0)	33.7 (32.8–34.7)	33.7 (33.1–34.3)	33.4 (32.9–33.8)	33.2 (32.5–33.8)	32.9 (32.4–33.5)	34.7 (34.2–35.2)	34.9 (34.4–35.3)	36.4 (35.7–37.1)	<0.001	4.60 (3.58 to 5.62)
Obesity	33.4 (32.1–34.7)	34.2 (33.3–35.1)	35.0 (34.1–35.9)	34.4 (33.9–35.0)	34.8 (34.0–35.6)	33.7 (32.9–34.5)	34.2 (33.4–34.9)	34.7 (34.1–35.3)	36.3 (35.8–36.9)	36.6 (36.2–37.1)	<0.001	3.19 (1.82 to 4.56)
*p* for interaction											<0.001 ^b^	<0.001 ^c^

^a^ Normal weight is defined as a body mass index (BMI) ranging from 18.5 to 24.9, overweight is defined as a BMI ranging from 25 to 29.9, and obesity is defined as a BMI ≥ 30. ^b^ *p* stands for the interaction assessing potential heterogeneous trends in energy intake from total carbohydrate, protein, and total fat by bodyweight status. ^c^ *p* stands for the interaction assessing potential heterogeneous changes in energy intake from total carbohydrate, protein, and total fat from 1999 to 2000 to 2017 to 2020 by bodyweight status.

## Data Availability

The datasets of the study are available from the corresponding author on reasonable request.

## Supplementary Materials

The following supporting information can be downloaded at: https://www.mdpi.com/article/10.3390/nu16162793/s1, Table S1: Dietary Components of Healthy Eating Index (HEI)-2020 and Scoring Standards; Table S2: Trends in Sociodemographic Characteristics of US Adults by Bodyweight Status, 1999–2020; Table S3: Adjusted Trends in Healthy Eating Index (HEI)-2020 Diet Score among US adults by Bodyweight Status, 1999–2020; Table S4: Adjusted Trends in components of Healthy Eating Index (HEI)-2020 Diet Score among US adults by Bodyweight Status, 1999–2020; Table S5: Trends in Dietary Intake of Key Food Groups and Nutrients Among US Adults by Bodyweight Status, 1999–2020; Table S6: Adjusted Trends in Dietary Intake of Key Food Groups and Nutrients Among US Adults by Bodyweight Status, 1999–2020; Table S7: Mean Change in Key Food Groups Intake among Obese Participants from 1999 to 2000 to 2017 to 2020, by Age; Table S8: Mean Change in Key Food Groups Intake among Obese Participants from 1999 to 2000 to 2017 to 2020, by Gender; Table S9: Mean Change in Key Food Groups Intake among Obese Participants from 1999 to 2000 to 2017 to 2020, by Race/Ethnicity; Table S10: Mean Change in Key Food Groups Intake among Obese Participants from 1999 to 2000 to 2017 to 2020, by Education.
