# Supplementary material for: Trends and Disparities in Diet Quality and Nutrient Intake among US Adults by Bodyweight Status
Source: Nutrients. 2024 Aug 21;16(16):2793. doi: 10.3390/nu16162793 (PMC11357175; doi:10.3390/nu16162793)
Supplement: Supplementary file 1 [file nutrients-16-02793-s001.zip › nutrients-3140066-supplementary.pdf]

Supplementary Tables

Title: Trends and disparities in diet quality and nutrient intake among US adults by bodyweight status

Table S1. Dietary Components of Healthy Eating Index (HEI)-2020 and Scoring Standards

Table S2. Trends in Sociodemographic Characteristics of US Adults by Body Weight Status, 1999-2020

Table S3. Adjusted Trends in Healthy Eating Index (HEI)-2020 Diet Score among US adults by Body Weight Status, 1999-2020

Table S4. Adjusted Trends in components of Healthy Eating Index (HEI)-2020 Diet Score among US adults by Body Weight Status, 1999-2020

Table S5. Trends in Dietary Intake of Key Food Groups and Nutrients Among US Adults by Body Weight Status, 1999-2020

Table S6. Adjusted Trends in Dietary Intake of Key Food Groups and Nutrients Among US Adults by Body Weight Status, 1999-2020

Table S7. Mean Change in Key Food Groups Intake among Obese Participants from 1999-2000 to 2017-2020, by Age

Table S8. Mean Change in Key Food Groups Intake among Obese Participants from 1999-2000 to 2017-2020, by Gender

Table S9. Mean Change in Key Food Groups Intake among Obese Participants from 1999-2000 to 2017-2020, by Race/Ethnicity

Table S10. Mean Change in Key Food Groups Intake among Obese Participants from 1999-2000 to 2017-2020, by Education

Table S1. Dietary Components of Healthy Eating Index (HEI)-2020 and Scoring Standards

Component	Points Range	Scoring Standard[a]	
		Max	Min
HEI-2020 Adequacy			
Total Fruits[b]	0-5	≥0.8 cup equiv. per 1,000 kcal	0
Whole Fruits[c]	0-5	≥0.4 cup equiv. per 1,000 kcal	0
Total Vegetables[d]	0-5	≥1.1 cup equiv. per 1,000 kcal	0
Greens and Beans[d]	0-5	≥0.2 cup equiv. per 1,000 kcal	0
Whole Grains	0-10	≥1.5 oz equiv. per 1,000 kcal	0
Dairy[e]	0-10	≥1.3 cup equiv. per 1,000 kcal	0
Total Protein Foods[f]	0-5	≥2.5 oz equiv. per 1,000 kcal	0
Seafood and Plant Proteins[e, g]	0-5	≥0.8 oz equiv. per 1,000 kcal	0
Fatty Acids[h]	0-10	(PUFAs + MUFAs)/SFAs ≥2.5	(PUFAs + MUFAs)/SFAs ≤1.2
HEI-2020 Moderation			
Refined Grains	0-10	≤1.8 oz equiv. per 1,000 kcal	≥4.3 oz equiv. per 1,000 kcal
Sodium	0-10	≤1.1 grams per 1,000 kcal	≥2.0 grams per 1,000 kcal
Added Sugars	0-10	≤6.5% of energy	≥26% of energy
Saturated Fats	0-10	≤8% of energy	≥16% of energy

[a] Intakes between the minimum and maximum standards are scored proportionately. [b] Includes 100% fruit juice. [c] Includes all forms except juice. [d] Includes legumes (beans and peas). [e] Includes all milk products, such as fluid milk, yogurt, and cheese, and fortified soy beverages. [f] Includes legumes (beans and peas). [g] Includes seafood, nuts, seeds, soy products (other than beverages), and legumes (beans and peas). [h] Ratios of poly-and monosaturated fatty acids (PUFAs and MUFAs) to saturated fatty acids (SFAs).

Table S2. Trends in Sociodemographic Characteristics of US Adults by Body Weight Status, 1999-2020[a]

Characteristics	1999-2000	2001-2002	2003-2004	2005-2006	2007-2008	2009-2010	2011-2012	2013-2014	2015-2016	2017-2020
	Survey-Weighted % (95% CI)									
Age, y, Mean (95% CI)										
Total population	44.3 (43.1-45.5)	46.1 (45.0-47.3)	47.4 (46.2-48.5)	47.4 (45.9-48.9)	46.8 (45.8-47.8)	47.6 (46.6-48.6)	47.6 (45.8-49.4)	48.1 (47.1-49.0)	48.4 (46.9-49.9)	48.6 (47.4-49.9)
Normal weight	41.8 (40.1-43.4)	43.0 (41.9-44.1)	45.2 (43.6-46.7)	44.1 (42.1-46.1)	44.3 (42.5-46.1)	44.1 (42.2-46.0)	45.2 (42.3-48.2)	45.3 (43.4-47.2)	45.5 (43.5-47.5)	44.9 (42.9-46.9)
Overweight	46.5 (44.6-48.4)	48.4 (47.1-49.7)	49.6 (48.1-51.1)	49.3 (47.3-51.3)	48.4 (46.4-50.3)	48.7 (47.6-49.9)	49.2 (47.8-50.7)	49.4 (48.1-50.7)	50.0 (47.7-52.2)	51.1 (49.4-52.8)
Obesity	45.0 (42.5-47.5)	46.9 (44.9-49.0)	47.2 (45.3-49.1)	48.6 (47.0-50.2)	47.4 (45.8-49.0)	49.2 (48.2-50.2)	48.1 (46.4-49.8)	49.0 (47.9-50.1)	49.0 (47.4-50.6)	48.9 (47.4-50.4)
% Male										
Total population	44.1 (39.2-49.2)	45.6 (43.6-47.6)	45.9 (42.4-49.4)	47.3 (45.8-48.9)	45.2 (43.5-46.9)	46.9 (45.6-48.2)	47.7 (45.8-49.6)	47.7 (45.5-50.0)	47.8 (46.1-49.4)	47.6 (45.4-49.8)
Normal weight	39.0 (31.7-46.7)	40.2 (35.1-45.5)	38.5 (33.3-43.9)	37.8 (33.6-42.1)	38.8 (35.7-42.0)	37.6 (32.7-42.7)	43.3 (39.3-47.3)	41.4 (37.3-45.6)	43.3 (40.0-46.6)	42.6 (36.7-48.7)
Overweight	54.1 (46.3-61.6)	56.2 (51.8-60.5)	51.8 (47.3-56.2)	59.3 (56.0-62.5)	52.7 (48.3-57.1)	54.3 (51.7-56.9)	53.6 (49.6-57.6)	57.4 (54.8-60.0)	54.9 (49.9-59.8)	51.5 (46.6-56.3)
Obesity	39.9 (33.7-46.4)	38.9 (35.0-43.1)	47.3 (40.7-54.0)	44.4 (40.0-49.0)	43.3 (40.2-46.5)	47.3 (43.9-50.8)	46.0 (42.0-50.0)	43.9 (40.3-47.6)	45.0 (39.9-50.2)	47.5 (43.3-51.8)
% Non-Hispanic White										
Total population	72.8 (68.8-76.5)	72.2 (67.5-76.4)	73.1 (65.5-79.6)	73.5 (66.9-79.2)	71.8 (64.7-78.0)	69.8 (62.4-76.2)	68.7 (60.9-75.6)	67.4 (60.7-73.4)	66.3 (58.6-73.2)	65.1 (59.5-70.4)
Normal weight	75.8 (68.6-81.8)	75.1 (68.9-80.5)	77.5 (70.2-83.4)	75.9 (69.1-81.7)	74.1 (67.1-80.0)	72.8 (66.1-78.6)	70.6 (62.0-78.0)	69.1 (62.9-74.6)	66.3 (58.7-73.1)	67.2 (61.4-72.5)
Overweight	74.3 (68.5-79.5)	71.2 (65.8-76.2)	73.7 (64.6-81.2)	74.2 (66.4-80.8)	72.8 (65.5-79.1)	71.6 (62.0-79.6)	73.1 (65.5-79.6)	69.8 (62.1-76.5)	68.9 (61.1-75.8)	65.2 (58.3-71.6)
Obesity	68.0 (62.4-	70.0 (65.0-	67.9 (59.7-	70.7 (62.9-	68.9 (59.3-	65.9 (58.5-	63.0 (53.4-	64.0 (57.3-	64.2 (55.6-	63.9 (57.6-

	73.1)	74.6)	75.2)	77.5)	77.1)	72.7)	71.6)	70.2)	72.0)	69.7)
% Non-Hispanic Black										
Total population	10.7 (8.1-14.1)	11.1 (8.3-14.7)	11.6 (8.2-16.1)	11.8 (8.2-16.8)	10.7 (7.3-15.5)	11.5 (9.7-13.5)	11.0 (7.3-16.4)	11.4 (8.5-15.1)	10.4 (6.9-15.4)	10.6 (8.1-13.8)
Normal weight	8.4 (5.8-11.9)	8.4 (5.9-11.9)	7.8 (4.9-12.2)	8.6 (5.5-13.2)	7.9 (5.6-11.1)	7.6 (5.8-9.9)	8.3 (5.0-13.7)	8.9 (6.2-12.6)	9.3 (6.1-14.0)	9.5 (7.3-12.3)
Overweight	8.2 (5.7-11.7)	10.3 (7.1-14.6)	9.9 (7.0-13.8)	10.7 (7.3-15.4)	9.6 (6.6-13.7)	9.4 (7.3-12.0)	8.6 (5.6-12.9)	8.7 (6.2-12.2)	8.4 (5.7-12.1)	8.7 (6.4-11.8)
Obesity	16.0 (10.9-22.7)	15.0 (11.2-19.8)	17.2 (12.5-23.2)	15.8 (11.0-22.1)	14.2 (9.0-21.7)	16.1 (12.5-20.5)	15.7 (10.3-23.1)	15.4 (11.5-20.5)	12.6 (7.9-19.6)	12.6 (9.4-16.7)
% Hispanic										
Total population	6.2 (3.4-11.0)	5.4 (2.7-10.5)	3.6 (2.2-5.7)	2.5 (1.6-3.9)	4.6 (2.8-7.3)	4.9 (2.8-8.4)	6.1 (3.6-10.1)	5.2 (3.6-7.4)	5.6 (3.4-9.1)	7.1 (5.7-8.8)
Normal weight	5.6 (2.5-12.0)	4.4 (2.3-8.5)	3.4 (1.9-6.1)	2.4 (1.4-4.2)	3.3 (1.8-6.1)	4.9 (2.9-8.4)	4.1 (2.2-7.5)	4.7 (3.2-6.8)	4.3 (2.2-8.2)	5.7 (3.6-8.7)
Overweight	9.0 (5.0-15.7)	6.1 (2.9-12.2)	4.2 (2.5-7.2)	2.1 (1.1-3.9)	5.9 (3.5-9.9)	5.2 (3.0-9.1)	6.1 (3.5-10.3)	5.7 (3.6-9.1)	5.6 (3.3-9.4)	7.9 (6.0-10.5)
Obesity	4.0 (1.9-8.2)	5.8 (2.7-11.8)	3.0 (1.8-5.0)	2.9 (1.8-4.7)	4.3 (2.7-6.7)	4.7 (2.4-8.8)	7.8 (4.7-12.6)	5.1 (3.4-7.4)	6.5 (4.0-10.2)	7.3 (5.7-9.3)
% High school or less than high school education										
Total population	48.2 (43.2-53.3)	45.4 (42.3-48.6)	43.8 (39.5-48.1)	42.0 (37.5-46.6)	44.1 (39.0-49.3)	39.9 (37.0-42.9)	35.1 (29.1-41.7)	35.1 (31.2-39.2)	34.0 (29.5-38.9)	35.3 (32.2-38.4)
Normal weight	41.0 (30.3-52.6)	41.1 (37.6-44.6)	37.3 (29.5-45.9)	37.8 (30.9-45.3)	40.2 (34.6-46.1)	33.5 (28.3-39.1)	29.9 (21.4-40.0)	32.3 (27.7-37.4)	26.9 (21.7-32.8)	32.6 (28.1-37.5)
Overweight	48.6 (41.3-56.0)	48.3 (42.9-53.7)	44.4 (39.5-49.4)	40.9 (35.8-46.2)	43.3 (36.3-50.6)	42.3 (38.1-46.6)	33.0 (28.1-38.3)	34.8 (29.4-40.6)	37.4 (30.1-45.5)	34.4 (30.4-38.6)
Obesity	55.8 (49.9-61.5)	46.9 (41.4-52.4)	49.6 (45.8-53.4)	46.8 (43.1-50.4)	48.1 (42.8-53.4)	42.7 (38.4-47.1)	41.6 (34.8-48.8)	37.3 (33.3-41.6)	35.9 (32.0-40.0)	37.4 (34.3-40.6)
% Some college, college or above education										

Total population	51.8 (46.7-56.8)	54.6 (51.4-57.7)	56.2 (51.9-60.5)	58.0 (53.4-62.5)	55.9 (50.7-61.0)	60.1 (57.1-63.0)	64.9 (58.3-70.9)	64.9 (60.8-68.8)	66.0 (61.1-70.5)	64.7 (61.6-67.8)
Normal weight	59.0 (47.4-69.7)	58.9 (55.4-62.4)	62.7 (54.1-70.5)	62.2 (54.7-69.1)	59.8 (53.9-65.4)	66.5 (60.9-71.7)	70.1 (60.0-78.6)	67.7 (62.6-72.3)	73.1 (67.2-78.3)	67.4 (62.5-71.9)
Overweight	51.4 (44.0-58.7)	51.7 (46.3-57.1)	55.6 (50.6-60.5)	59.1 (53.8-64.2)	56.7 (49.4-63.7)	57.7 (53.4-61.9)	67.0 (61.7-71.9)	65.2 (59.4-70.6)	62.6 (54.5-69.9)	65.6 (61.4-69.6)
Obesity	44.2 (38.5-50.1)	53.1 (47.6-58.6)	50.4 (46.6-54.2)	53.2 (49.6-56.9)	51.9 (46.6-57.2)	57.3 (52.9-61.6)	58.4 (51.2-65.2)	62.7 (58.4-66.7)	64.1 (60.0-68.0)	62.6 (59.4-65.7)
FIPR, mean (95% CI)[b]										
Total population	2.86 (2.64-3.07)	3.03 (2.88-3.17)	3.00 (2.84-3.17)	3.15 (3.00-3.30)	3.04 (2.83-3.24)	3.00 (2.89-3.12)	2.96 (2.74-3.18)	2.97 (2.75-3.20)	3.03 (2.84-3.23)	3.17 (3.05-3.29)
Normal weight	2.89 (2.62-3.16)	3.08 (2.87-3.29)	3.03 (2.79-3.26)	3.26 (3.03-3.49)	3.12 (2.90-3.33)	3.09 (2.92-3.25)	3.04 (2.75-3.34)	3.09 (2.74-3.44)	3.10 (2.84-3.36)	3.14 (2.96-3.31)
Overweight	2.98 (2.70-3.26)	3.16 (2.98-3.34)	3.14 (3.01-3.27)	3.23 (3.07-3.39)	3.07 (2.83-3.31)	3.12 (2.93-3.31)	3.12 (2.88-3.35)	3.09 (2.80-3.38)	3.12 (2.94-3.29)	3.34 (3.18-3.51)
Obesity	2.69 (2.45-2.93)	2.81 (2.63-2.99)	2.83 (2.61-3.05)	2.97 (2.82-3.11)	2.93 (2.67-3.20)	2.84 (2.69-2.98)	2.75 (2.49-3.01)	2.78 (2.63-2.94)	2.93 (2.67-3.19)	3.07 (2.94-3.20)
% Lower-income (FIPR < 1.30)										
Total population	25.1 (21.3-29.3)	21.0 (18.7-23.6)	19.9 (16.3-24.2)	15.7 (13.6-18.0)	20.0 (16.3-24.2)	22.0 (18.9-25.3)	23.7 (20.2-27.7)	23.7 (19.2-28.8)	19.3 (15.9-23.3)	18.3 (16.2-20.5)
Normal weight	26.9 (19.7-35.6)	21.3 (18.2-24.8)	21.6 (16.8-27.3)	13.6 (11.1-16.5)	19.2 (16.1-22.7)	19.2 (15.9-23.0)	22.7 (17.9-28.2)	23.6 (17.9-30.6)	17.2 (13.4-21.8)	19.9 (17.1-23.1)
Overweight	22.1 (17.5-27.5)	17.1 (14.2-20.3)	16.9 (13.4-21.0)	15.4 (13.3-17.6)	18.0 (14.5-22.1)	21.4 (16.9-26.6)	22.0 (18.1-26.4)	22.8 (17.4-29.2)	17.2 (13.9-20.9)	16.0 (13.3-19.1)
Obesity	26.2 (21.5-31.5)	25.5 (21.5-29.9)	21.5 (16.9-27.0)	17.9 (14.5-21.9)	22.6 (16.8-29.7)	24.5 (21.2-28.2)	26.2 (21.4-31.6)	24.5 (20.2-29.4)	22.3 (18.0-27.2)	19.0 (16.7-21.6)
% Higher-income (FIPR ⩾ 1.30)										
Total population	74.9 (70.7-78.7)	79.0 (76.4-81.3)	80.1 (75.8-83.7)	84.3 (82.0-86.4)	80.0 (75.8-83.7)	78.0 (74.7-81.1)	76.3 (72.3-79.8)	76.3 (71.2-80.8)	80.7 (76.7-84.1)	81.7 (79.5-83.8)

Normal weight	73.1 (64.4-80.3)	78.7 (75.2-81.8)	78.4 (72.7-83.2)	86.4 (83.5-88.9)	80.8 (77.3-83.9)	80.8 (77.0-84.1)	77.3 (71.8-82.1)	76.4 (69.4-82.1)	82.8 (78.2-86.6)	80.1 (76.9-82.9)
Overweight	77.9 (72.5-82.5)	82.9 (79.7-85.8)	83.1 (79.0-86.6)	84.6 (82.4-86.7)	82.0 (77.9-85.5)	78.6 (73.4-83.1)	78.0 (73.6-81.9)	77.2 (70.8-82.6)	82.8 (79.1-86.1)	84.0 (80.9-86.7)
Obesity	73.8 (68.5-78.5)	74.5 (70.1-78.5)	78.5 (73.0-83.1)	82.1 (78.1-85.5)	77.4 (70.3-83.2)	75.5 (71.8-78.8)	73.8 (68.4-78.6)	75.5 (70.6-79.8)	77.7 (72.8-82.0)	81.0 (78.4-83.3)

Abbreviations: FIPR: Family income to poverty ratio. [a] Normal weight is defined as a body mass index (BMI) ranging from 18.5 to 24.9, overweight is defined as a BMI ranging from 25 to 29.9, and obesity is defined as a BMI ≥ 30. [b] Family income to poverty ratio represents the ratio of family income to the federal poverty threshold, adjusting for household size. A lower ratio indicates a lower level of income.

Table S3. Adjusted Trends in Healthy Eating Index (HEI)-2020 Diet Score among US adults by Body Weight Status, 1999-2020[a].

Dietary Score	Survey-Weighted Mean Score (95% CI)										*p* for Trend	Change From 1999-2020, Mean (95% CI)
	**1999-2000 (*n* =1289)**	**2001-2002 (*n* =2774)**	**2003-2004 (*n* = 3053)**	**2005-2006 (*n* = 3204)**	**2007-2008 (*n* = 3731)**	**2009-2010 (*n* = 4012)**	**2011-2012 (*n* = 3382)**	**2013-2014 (*n* = 3876)**	**2015-2016 (*n* = 3786)**	**2017-2020 (*n* = 5522)**		
HEI-2020 diet score (unadjusted)												
Total population	45.4 (43.6-47.1)	48.9 (48.2-49.7)	49.2 (48.2-50.2)	50.1 (49.2-51.0)	50.4 (49.2-51.5)	51.2 (50.6-51.8)	52.0 (51.2-52.8)	51.2 (50.5-51.8)	50.6 (49.3-51.9)	50.1 (49.2-51.0)	<0.001	4.67 (2.70 to 6.64)
Normal weight	46.6 (44.4-48.9)	50.2 (48.9-51.6)	49.6 (47.9-51.4)	50.8 (49.5-52.2)	51.1 (49.4-52.8)	52.6 (51.7-53.4)	54.1 (52.7-55.4)	53.1 (51.6-54.6)	52.5 (50.8-54.2)	51.6 (49.7-53.5)	0.010	4.97 (2.02 to 7.93)
Overweight	45.6 (43.5-47.6)	49.1 (48.0-50.3)	50.0 (48.8-51.2)	51.1 (50.2-52.0)	50.4 (48.9-52.0)	51.9 (51.1-52.8)	52.7 (51.7-53.7)	51.9 (50.4-53.5)	50.7 (48.9-52.5)	51.1 (50.2-52.1)	<0.001	5.58 (3.31 to 7.85)
Obesity	43.8 (42.0-45.7)	47.3 (46.1-48.4)	47.9 (47.2-48.6)	48.5 (47.6-49.3)	49.7 (48.6-50.8)	49.6 (48.7-50.5)	49.5 (48.7-50.4)	49.1 (48.6-49.6)	49.4 (48.2-50.6)	48.4 (47.5-49.3)	<0.001	4.55 (2.47 to 6.63)
p for interaction											<0.001[b]	<0.001[c]
HEI-2020 diet score (adjusted for differences in age, sex, and race/ethnicity over time)												
Total population	45.8 (44.1-47.6)	49.1 (48.3-49.9)	49.2 (48.2-50.2)	50.1 (49.3-50.9)	50.4 (49.3-51.6)	51.2 (50.6-51.8)	52.0 (51.2-52.8)	51.1 (50.4-51.8)	50.5 (49.3-51.7)	49.9 (49.0-50.8)	<0.001	4.08 (2.13 to 6.04)
Normal weight	47.0 (44.8-49.3)	50.5 (49.1-51.9)	49.5 (47.8-51.2)	50.8 (49.7-52.0)	51.1 (49.5-52.7)	52.6 (51.8-53.3)	54.0 (52.8-55.2)	53.0 (51.6-54.4)	52.4 (50.9-54.0)	51.6 (49.8-53.4)	<0.001	4.56 (1.67 to 7.45)
Overweight	46.0 (43.8-48.1)	49.3 (48.2-50.4)	49.9 (48.5-51.2)	51.2 (50.2-52.1)	50.5 (48.9-52.1)	52.0 (51.1-52.9)	52.7 (51.7-53.8)	52.0 (50.5-53.4)	50.6 (48.7-52.5)	50.8 (49.9-51.8)	<0.001	4.86 (2.48 to 7.24)
Obesity	44.3 (42.6-46.1)	47.4 (46.2-48.6)	48.1 (47.6-48.7)	48.4 (47.7-49.1)	49.8 (48.7-50.8)	49.5 (48.6-50.4)	49.5 (48.6-50.4)	49.0 (48.4-49.5)	49.3 (48.2-50.3)	48.3 (47.4-49.2)	<0.001	3.97 (1.98 to 5.97)
p for interaction											<0.001[b]	<0.001[c]
HEI-2020 diet score (adjusted for differences in age, sex, race/ethnicity, and education over time)												
Total population	46.4 (44.9-47.9)	49.5 (48.8-50.1)	49.5 (48.7-50.3)	50.3 (49.6-50.9)	50.7 (49.8-51.6)	51.3 (50.6-51.9)	51.8 (51.1-52.4)	50.9 (50.3-51.4)	50.2 (49.2-51.2)	49.5 (48.8-50.3)	<0.001	3.11 (1.43 to 4.78)

Normal weight	47.7 (46.1-49.3)	50.9 (49.7-52.2)	49.7 (48.5-51.0)	51.1 (50.1-52.1)	51.6 (50.3-52.8)	52.5 (51.7-53.3)	53.7 (52.4-55.0)	52.7 (51.6-53.9)	51.9 (50.4-53.3)	51.2 (49.7-52.8)	<0.001	3.52 (1.29 to 5.76)
Overweight	46.4 (44.4-48.5)	49.7 (48.8-50.7)	50.1 (48.9-51.3)	51.3 (50.4-52.2)	50.7 (49.3-52.1)	52.1 (51.2-53.1)	52.4 (51.5-53.4)	51.8 (50.4-53.2)	50.4 (48.7-52.1)	50.4 (49.5-51.3)	<0.001	3.98 (1.71 to 6.24)
Obesity	44.9 (43.1-46.7)	47.6 (46.5-48.7)	48.4 (47.8-49.0)	48.6 (47.8-49.3)	50.0 (49.0-51.0)	49.6 (48.6-50.5)	49.4 (48.6-50.3)	48.8 (48.3-49.3)	49.0 (48.1-50.0)	48.0 (47.2-48.8)	<0.001	3.09 (1.10 to 5.08)
p for interaction											<0.001[b]	<0.001[c]
HEI-2020 diet score (adjusted for differences in age, sex, race/ethnicity, and income over time)												
Total population	46.0 (44.4-47.5)	49.1 (48.4-49.8)	49.2 (48.3-50.1)	50.0 (49.3-50.7)	50.4 (49.3-51.5)	51.2 (50.6-51.9)	52.1 (51.3-52.8)	51.2 (50.6-51.8)	50.5 (49.5-51.6)	49.8 (48.9-50.6)	<0.001	3.78 (2.00 to 5.55)
Normal weight	47.3 (45.3-49.3)	50.5 (49.2-51.8)	49.6 (48.1-51.1)	50.6 (49.5-51.7)	51.1 (49.6-52.6)	52.6 (51.9-53.3)	54.1 (53.0-55.2)	53.0 (51.8-54.2)	52.4 (51.0-53.9)	51.5 (49.7-53.4)	<0.001	4.25 (1.53 to 6.96)
Overweight	46.1 (44.1-48.1)	49.3 (48.3-50.3)	49.8 (48.6-51.1)	51.1 (50.3-52.0)	50.6 (49.1-52.1)	52.0 (51.0-53.0)	52.8 (51.7-53.8)	52.0 (50.7-53.4)	50.7 (48.9-52.4)	50.6 (49.7-51.6)	<0.001	4.54 (2.33 to 6.76)
Obesity	44.5 (42.8-46.2)	47.4 (46.3-48.6)	48.2 (47.6-48.7)	48.3 (47.7-49.0)	49.7 (48.6-50.7)	49.6 (48.7-50.5)	49.7 (48.7-50.7)	49.0 (48.6-49.5)	49.2 (48.3-50.2)	48.2 (47.3-49.0)	<0.001	3.71 (1.81 to 5.61)
p for interaction											<0.001[b]	<0.001[c]

Abbreviations: HEI, Healthy Eating Index. [a] Normal weight is defined as a body mass index (BMI) ranging from 18.5 to 24.9, overweight is defined as a BMI ranging from 25 to 29.9, and obesity is defined as a BMI ≥ 30. [b] *p* for interaction assessing potential heterogeneous trends in HEI-2020 diet score by body weight status. [c] *p* for interaction assessing potential heterogeneous changes in HEI-2020 diet score from 1999-2000 to 2017-2020 by body weight status.

Table S4. Adjusted Trends in components of Healthy Eating Index (HEI)-2020 Diet Score among US adults by Body Weight Status, 1999-2020[a,b].

Dietary Score	Survey-Weighted Mean Score (95% CI)										*p* for Trend	Change From 1999-2020, Mean (95% CI)
	1999-2000 (*n* =1289)	2001-2002 (*n* =2774)	2003-2004 (*n* = 3053)	2005-2006 (*n* = 3204)	2007-2008 (*n* = 3731)	2009-2010 (*n* = 4012)	2011-2012 (*n* = 3382)	2013-2014 (*n* = 3876)	2015-2016 (*n* = 3786)	2017-2020 (*n* = 5522)		
HEI-2020 diet score												
Total population	45.8 (44.1-47.6)	49.1 (48.3-49.9)	49.2 (48.2-50.2)	50.1 (49.3-50.9)	50.4 (49.3-51.6)	51.2 (50.6-51.8)	52.0 (51.2-52.8)	51.1 (50.4-51.8)	50.5 (49.3-51.7)	49.9 (49.0-50.8)	<0.001	4.08 (2.13 to 6.04)
Normal weight	47.0 (44.8-49.3)	50.5 (49.1-51.9)	49.5 (47.8-51.2)	50.8 (49.7-52.0)	51.1 (49.5-52.7)	52.6 (51.8-53.3)	54.0 (52.8-55.2)	53.0 (51.6-54.4)	52.4 (50.9-54.0)	51.6 (49.8-53.4)	<0.001	4.56 (1.67 to 7.45)
Overweight	46.0 (43.8-48.1)	49.3 (48.2-50.4)	49.9 (48.5-51.2)	51.2 (50.2-52.1)	50.5 (48.9-52.1)	52.0 (51.1-52.9)	52.7 (51.7-53.8)	52.0 (50.5-53.4)	50.6 (48.7-52.5)	50.8 (49.9-51.8)	<0.001	4.86 (2.48 to 7.24)
Obesity	44.3 (42.6-46.1)	47.4 (46.2-48.6)	48.1 (47.6-48.7)	48.4 (47.7-49.1)	49.8 (48.7-50.8)	49.5 (48.6-50.4)	49.5 (48.6-50.4)	49.0 (48.4-49.5)	49.3 (48.2-50.3)	48.3 (47.4-49.2)	<0.001	3.97 (1.98 to 5.97)
p for interaction											<0.001[c]	<0.001[d]
Total fruits												
Total population	2.1 (1.7-2.4)	2.2 (2.1-2.3)	2.1 (1.9-2.3)	2.2 (2.1-2.3)	2.2 (2.0-2.3)	2.3 (2.2-2.3)	2.2 (2.1-2.3)	2.1 (2.0-2.2)	2.0 (1.8-2.1)	1.9 (1.8-2.0)	<0.001	-0.13 (-0.46 to 0.20)
Normal weight	2.2 (1.8-2.7)	2.4 (2.2-2.7)	2.1 (1.9-2.3)	2.3 (2.1-2.4)	2.2 (2.0-2.4)	2.4 (2.3-2.6)	2.5 (2.3-2.6)	2.2 (2.1-2.3)	2.2 (2.0-2.3)	2.0 (1.8-2.2)	<0.001	-0.22 (-0.71 to 0.26)
Overweight	1.9 (1.7-2.2)	2.1 (2.0-2.3)	2.2 (2.1-2.4)	2.3 (2.1-2.5)	2.2 (2.1-2.4)	2.4 (2.2-2.5)	2.3 (2.1-2.5)	2.2 (2.0-2.3)	2.0 (1.8-2.3)	2.0 (1.9-2.1)	<0.001	0.07 (-0.19 to 0.34)
Obesity	2.0 (1.6-2.3)	1.9 (1.7-2.1)	2.0 (1.8-2.2)	2.1 (1.9-2.2)	2.0 (1.9-2.2)	2.1 (2.0-2.2)	1.9 (1.8-2.0)	1.9 (1.8-2.0)	1.9 (1.7-2.0)	1.8 (1.7-2.0)	<0.001	-0.16 (-0.54 to 0.22)
p for interaction											<0.001[c]	<0.001[d]
Whole fruits												
Total population	1.8 (1.5-2.0)	2.0 (1.8-2.1)	1.9 (1.8-2.1)	2.1 (1.9-2.2)	2.1 (2.0-2.3)	2.2 (2.2-2.3)	2.2 (2.1-2.4)	2.1 (2.1-2.2)	2.0 (1.9-2.2)	2.0 (1.9-2.1)	<0.001	0.23 (-0.05 to 0.50)
Normal weight	1.9 (1.6-2.2)	2.2 (2.0-2.4)	1.9 (1.7-2.2)	2.1 (1.9-2.2)	2.2 (2.0-2.4)	2.4 (2.2-2.6)	2.4 (2.1-2.6)	2.3 (2.1-2.5)	2.3 (2.0-2.5)	2.1 (1.9-2.3)	<0.001	0.21 (-0.17 to 0.60)
Overweight	1.6 (1.4-1.8)	2.0 (1.8-2.1)	2.1 (1.9-2.3)	2.2 (2.0-2.4)	2.2 (2.0-2.5)	2.4 (2.2-2.5)	2.3 (2.1-2.6)	2.3 (2.1-2.5)	2.1 (1.9-2.3)	2.2 (2.0-2.3)	<0.001	0.55 (0.30 to 0.80)
Obesity	1.8 (1.5-2.2)	1.7 (1.5-1.9)	1.8 (1.6-2.0)	2.0 (1.8-2.1)	2.0 (1.8-2.2)	2.0 (1.9-2.1)	1.9 (1.8-2.1)	1.9 (1.8-2.0)	1.9 (1.7-2.1)	1.8 (1.7-2.0)	<0.001	0.02 (-0.39 to 0.42)

p for interaction											<0.001[c]	<0.001[d]
Total vegetables												
Total population	3.6 (3.4-3.7)	3.1 (3.0-3.1)	3.2 (3.1-3.3)	3.2 (3.1-3.2)	3.2 (3.1-3.2)	3.1 (3.1-3.2)	3.1 (3.1-3.2)	3.1 (3.0-3.1)	3.1 (3.0-3.1)	2.9 (2.9-3.0)	<0.001	-0.63 (-0.80 to -0.46)
Normal weight	3.5 (3.3-3.7)	3.2 (3.0-3.3)	3.1 (3.0-3.3)	3.2 (3.1-3.3)	3.2 (3.1-3.4)	3.2 (3.0-3.3)	3.2 (3.0-3.3)	3.0 (2.8-3.2)	3.2 (3.1-3.3)	3.0 (2.8-3.1)	<0.001	-0.52 (-0.74 to -0.29)
Overweight	3.6 (3.3-3.8)	3.0 (2.9-3.1)	3.2 (3.1-3.4)	3.1 (3.0-3.2)	3.1 (3.0-3.2)	3.1 (3.0-3.2)	3.2 (3.1-3.3)	3.2 (3.0-3.3)	3.0 (2.8-3.2)	3.0 (2.9-3.2)	<0.001	-0.56 (-0.88 to -0.23)
Obesity	3.7 (3.5-3.9)	3.1 (2.9-3.2)	3.1 (3.0-3.2)	3.2 (3.1-3.3)	3.2 (3.0-3.3)	3.1 (3.0-3.1)	3.0 (2.9-3.2)	3.0 (2.9-3.1)	3.0 (2.9-3.1)	2.9 (2.8-3.0)	<0.001	-0.80 (-1.03 to -0.57)
P for interaction											<0.001[c]	<0.001[d]
Greens and beans												
Total population	1.0 (0.9-1.2)	1.3 (1.1-1.4)	1.3 (1.2-1.4)	1.4 (1.2-1.5)	1.4 (1.2-1.5)	1.4 (1.3-1.5)	1.5 (1.4-1.6)	1.6 (1.5-1.7)	1.6 (1.5-1.8)	1.5 (1.4-1.6)	<0.001	0.44 (0.24 to 0.64)
Normal weight	1.3 (0.9-1.6)	1.3 (1.1-1.5)	1.4 (1.2-1.5)	1.5 (1.3-1.7)	1.5 (1.3-1.6)	1.6 (1.4-1.7)	1.7 (1.6-1.8)	1.7 (1.5-1.9)	1.9 (1.6-2.1)	1.6 (1.4-1.8)	<0.001	0.38 (0.00 to 0.76)
Overweight	1.1 (0.9-1.3)	1.3 (1.1-1.4)	1.4 (1.2-1.5)	1.3 (1.2-1.5)	1.3 (1.1-1.5)	1.4 (1.2-1.5)	1.6 (1.4-1.8)	1.6 (1.4-1.8)	1.6 (1.4-1.8)	1.6 (1.4-1.8)	<0.001	0.51 (0.23 to 0.79)
Obesity	0.8 (0.6-0.9)	1.2 (1.0-1.4)	1.2 (1.1-1.3)	1.3 (1.2-1.5)	1.3 (1.2-1.4)	1.3 (1.2-1.4)	1.3 (1.2-1.4)	1.4 (1.3-1.6)	1.5 (1.4-1.6)	1.3 (1.2-1.4)	<0.001	0.54 (0.35 to 0.72)
p for interaction											<0.001[c]	<0.001[d]
Whole grains												
Total population	1.6 (1.3-1.8)	2.2 (2.0-2.3)	2.0 (1.8-2.2)	2.3 (2.2-2.5)	2.3 (2.1-2.5)	2.6 (2.5-2.7)	2.8 (2.6-3.0)	2.7 (2.6-2.8)	2.7 (2.5-2.9)	2.4 (2.3-2.6)	<0.001	0.88 (0.60 to 1.15)
Normal weight	1.6 (1.2-1.9)	2.2 (1.9-2.5)	2.0 (1.8-2.3)	2.3 (2.0-2.5)	2.3 (2.1-2.5)	2.8 (2.6-3.1)	3.2 (2.9-3.5)	3.1 (2.8-3.3)	2.8 (2.5-3.1)	2.8 (2.5-3.2)	<0.001	1.28 (0.77 to 1.79)
Overweight	1.8 (1.3-2.2)	2.2 (1.9-2.4)	2.0 (1.8-2.2)	2.5 (2.3-2.8)	2.4 (2.1-2.6)	2.6 (2.4-2.8)	2.8 (2.6-3.1)	2.8 (2.5-3.1)	2.7 (2.4-3.1)	2.4 (2.1-2.6)	<0.001	0.62 (0.15 to 1.09)
Obesity	1.4 (1.2-1.5)	2.1 (1.8-2.4)	1.9 (1.8-2.1)	2.1 (2.0-2.3)	2.2 (2.1-2.4)	2.4 (2.2-2.5)	2.5 (2.3-2.7)	2.4 (2.2-2.6)	2.6 (2.5-2.8)	2.3 (2.1-2.4)	<0.001	0.90 (0.67 to 1.14)
p for interaction											<0.001[c]	<0.001[d]
Dairy												
Total population	5.7 (5.4-6.0)	4.8 (4.6-5.0)	4.8 (4.5-5.0)	5.1 (5.0-5.3)	5.1 (4.8-5.3)	5.4 (5.3-5.5)	5.3 (5.1-5.4)	5.2 (5.0-5.4)	5.0 (4.8-5.3)	4.8 (4.6-4.9)	<0.001	-0.95 (-1.28 to -0.62)
Normal weight	5.8 (5.4-6.2)	5.1 (4.7-5.6)	5.0 (4.7-5.3)	5.1 (4.9-5.4)	5.2 (4.9-5.5)	5.4 (5.2-5.6)	5.4 (5.2-5.6)	5.4 (5.1-5.6)	5.3 (5.1-5.5)	4.6 (4.4-4.8)	<0.001	-1.26 (-1.72 to -0.79)
Overweight	5.4 (5.1-5.7)	4.8 (4.5-5.1)	4.6 (4.3-4.9)	5.3 (5.0-5.6)	5.0 (4.8-5.3)	5.4 (5.2-5.6)	5.3 (5.0-5.5)	5.2 (5.0-5.5)	5.1 (4.6-5.6)	4.8 (4.6-5.0)	<0.001	-0.61 (-0.96 to -0.27)
Obesity	5.9 (5.2-6.6)	4.4 (4.1-4.6)	4.6 (4.4-4.9)	5.0 (4.8-5.2)	4.9 (4.6-5.3)	5.3 (5.1-5.5)	5.2 (4.8-5.5)	5.0 (4.7-5.2)	4.8 (4.6-5.0)	4.9 (4.6-5.1)	<0.001	-1.02 (-1.75 to -0.30)

p for interaction											<0.001[c]	<0.001[d]
Total protein foods												
Total population	4.6 (4.5-4.6)	4.2 (4.2-4.2)	4.2 (4.1-4.2)	4.3 (4.2-4.3)	4.3 (4.2-4.3)	4.3 (4.2-4.3)	4.2 (4.1-4.3)	4.2 (4.2-4.3)	4.3 (4.2-4.3)	4.3 (4.2-4.3)	<0.001	-0.32 (-0.39 to -0.25)
Normal weight	4.4 (4.3-4.6)	4.2 (4.1-4.3)	4.1 (4.0-4.2)	4.2 (4.1-4.3)	4.2 (4.1-4.3)	4.2 (4.1-4.3)	4.1 (4.0-4.2)	4.1 (4.0-4.2)	4.2 (4.1-4.3)	4.3 (4.2-4.4)	<0.001	-0.18 (-0.35 to 0.00)
Overweight	4.7 (4.6-4.7)	4.2 (4.1-4.3)	4.2 (4.1-4.3)	4.3 (4.2-4.3)	4.3 (4.2-4.4)	4.3 (4.2-4.3)	4.3 (4.2-4.4)	4.3 (4.2-4.4)	4.3 (4.2-4.3)	4.3 (4.2-4.4)	<0.001	-0.40 (-0.53 to -0.27)
Obesity	4.6 (4.6-4.7)	4.2 (4.1-4.3)	4.3 (4.2-4.4)	4.3 (4.3-4.4)	4.4 (4.3-4.5)	4.4 (4.3-4.4)	4.3 (4.2-4.4)	4.3 (4.3-4.4)	4.3 (4.3-4.4)	4.3 (4.2-4.3)	<0.001	-0.39 (-0.50 to -0.27)
p for interaction											<0.001[c]	<0.001[d]
Seafood and plant proteins												
Total population	1.9 (1.7-2.0)	1.9 (1.8-2.0)	2.1 (2.0-2.2)	2.1 (2.0-2.3)	2.2 (2.0-2.3)	2.2 (2.1-2.3)	2.3 (2.2-2.4)	2.3 (2.2-2.4)	2.4 (2.2-2.5)	2.3 (2.2-2.4)	<0.001	0.46 (0.25 to 0.67)
Normal weight	2.1 (1.8-2.4)	1.9 (1.7-2.1)	2.1 (2.0-2.2)	2.3 (2.1-2.4)	2.4 (2.1-2.6)	2.4 (2.2-2.5)	2.5 (2.3-2.6)	2.6 (2.4-2.8)	2.6 (2.4-2.9)	2.5 (2.3-2.7)	<0.001	0.41 (0.01 to 0.81)
Overweight	1.6 (1.4-1.8)	2.0 (1.8-2.1)	2.1 (2.0-2.3)	2.2 (2.1-2.4)	2.2 (1.9-2.4)	2.3 (2.1-2.4)	2.4 (2.3-2.5)	2.3 (2.1-2.5)	2.4 (2.2-2.6)	2.5 (2.3-2.7)	<0.001	0.90 (0.62 to 1.18)
Obesity	1.9 (1.6-2.2)	1.8 (1.7-2.0)	2.0 (1.8-2.2)	2.0 (1.8-2.1)	2.0 (1.8-2.2)	2.1 (1.9-2.2)	2.0 (1.9-2.2)	2.1 (2.0-2.2)	2.2 (2.1-2.4)	2.1 (2.0-2.2)	<0.001	0.21 (-0.09 to 0.51)
p for interaction											<0.001[c]	<0.001[d]
Fatty acids												
Total population	4.3 (4.1-4.5)	4.8 (4.6-4.9)	5.0 (4.9-5.1)	4.6 (4.5-4.8)	4.8 (4.6-5.0)	4.9 (4.8-5.0)	5.1 (5.0-5.3)	4.9 (4.8-5.1)	4.8 (4.6-5.0)	4.9 (4.7-5.0)	<0.001	0.54 (0.27 to 0.81)
Normal weight	4.3 (3.7-4.8)	4.6 (4.3-5.0)	5.1 (4.8-5.4)	4.6 (4.5-4.8)	4.7 (4.3-5.0)	4.9 (4.7-5.1)	5.3 (5.0-5.5)	5.0 (4.7-5.2)	4.7 (4.4-5.0)	5.0 (4.7-5.4)	<0.001	0.76 (0.09 to 1.43)
Overweight	4.4 (3.9-4.9)	4.7 (4.5-4.9)	5.1 (4.9-5.4)	4.7 (4.4-5.0)	4.8 (4.6-5.1)	5.0 (4.8-5.2)	5.0 (4.8-5.2)	4.9 (4.6-5.2)	4.7 (4.3-5.1)	5.0 (4.7-5.2)	0.001	0.55 (0.00 to 1.10)
Obesity	4.2 (3.8-4.7)	5.0 (4.8-5.2)	4.9 (4.6-5.1)	4.6 (4.4-4.8)	4.9 (4.7-5.1)	4.8 (4.6-5.0)	5.1 (4.9-5.4)	4.9 (4.8-5.1)	4.9 (4.7-5.1)	4.7 (4.5-4.9)	0.008	0.41 (-0.11 to 0.93)
p for interaction											<0.001[c]	0.004[d]
Refined grains												
Total population	3.8 (3.5-4.2)	5.8 (5.6-6.0)	5.7 (5.6-5.9)	6.0 (5.9-6.2)	6.1 (5.9-6.3)	6.0 (5.9-6.2)	6.0 (5.8-6.2)	6.1 (5.9-6.2)	6.2 (6.0-6.4)	6.1 (6.0-6.3)	<0.001	2.28 (1.92 to 2.64)
Normal weight	3.9 (3.2-4.6)	6.0 (5.7-6.4)	5.6 (5.3-5.9)	6.2 (6.0-6.5)	6.1 (5.8-6.4)	6.2 (5.9-6.5)	6.2 (5.8-6.5)	6.2 (6.0-6.4)	6.5 (6.1-6.9)	6.2 (5.9-6.5)	<0.001	2.35 (1.60 to 3.10)
Overweight	4.0 (3.5-4.5)	5.8 (5.6-6.0)	5.7 (5.4-5.9)	6.2 (5.9-6.4)	6.0 (5.8-6.3)	6.2 (5.9-6.4)	6.2 (5.9-6.5)	6.3 (5.9-6.6)	6.1 (5.7-6.4)	6.1 (5.8-6.4)	<0.001	2.12 (1.53 to 2.72)
Obesity	3.6 (3.2-4.1)	5.4 (4.9-5.9)	5.9 (5.6-6.1)	5.7 (5.5-6.0)	6.2 (5.9-6.5)	5.8 (5.6-6.0)	5.7 (5.6-5.9)	5.8 (5.6-6.0)	6.1 (5.8-6.4)	6.1 (5.9-6.2)	<0.001	2.45 (1.99 to 2.90)

p for interaction											<0.001[c]	<0.001[d]
Sodium												
Total population	5.3 (4.8-5.7)	5.3 (5.1-5.5)	4.8 (4.6-5.1)	4.4 (4.2-4.6)	4.4 (4.4-4.5)	3.9 (3.7-4.1)	4.2 (4.1-4.2)	4.1 (3.9-4.3)	4.0 (3.8-4.2)	4.5 (4.3-4.7)	<0.001	-0.75 (-1.21 to -0.29)
Normal weight	5.6 (5.0-6.3)	5.4 (5.1-5.8)	4.8 (4.6-5.1)	4.7 (4.4-5.0)	4.6 (4.3-4.8)	4.2 (3.9-4.6)	4.6 (4.3-4.8)	4.5 (4.2-4.9)	4.1 (3.8-4.5)	4.8 (4.5-5.1)	<0.001	-0.82 (-1.55 to -0.10)
Overweight	5.2 (4.7-5.8)	5.4 (5.1-5.7)	4.9 (4.6-5.2)	4.7 (4.5-4.9)	4.5 (4.4-4.7)	4.0 (3.8-4.2)	4.1 (3.8-4.3)	4.0 (3.8-4.2)	4.1 (3.9-4.3)	4.6 (4.3-4.9)	<0.001	-0.63 (-1.25 to -0.01)
Obesity	4.8 (4.4-5.3)	5.0 (4.7-5.4)	4.8 (4.5-5.1)	3.9 (3.7-4.1)	4.2 (4.0-4.4)	3.6 (3.5-3.7)	3.9 (3.7-4.1)	3.9 (3.6-4.2)	3.8 (3.6-4.1)	4.3 (4.0-4.5)	<0.001	-0.57 (-1.09 to -0.05)
p for interaction											<0.001[c]	0.002[d]
Added sugars												
Total population	4.2 (3.9-4.5)	5.5 (5.2-5.8)	6.1 (5.8-6.4)	6.5 (6.3-6.7)	6.4 (6.1-6.7)	6.6 (6.5-6.8)	6.7 (6.5-6.9)	6.7 (6.5-6.9)	6.9 (6.7-7.1)	7.0 (6.8-7.2)	<0.001	2.74 (2.38 to 3.09)
Normal weight	4.1 (3.6-4.7)	5.5 (4.9-6.0)	5.9 (5.5-6.3)	6.5 (6.1-6.8)	6.4 (6.0-6.7)	6.4 (6.1-6.7)	6.5 (6.2-6.8)	6.4 (6.0-6.9)	6.9 (6.6-7.3)	7.0 (6.7-7.3)	<0.001	2.89 (2.22 to 3.56)
Overweight	4.5 (4.2-4.9)	5.6 (5.2-6.0)	6.2 (5.9-6.6)	6.5 (6.3-6.7)	6.3 (5.8-6.7)	6.8 (6.6-7.0)	7.0 (6.6-7.3)	7.1 (6.8-7.5)	7.0 (6.6-7.4)	7.1 (6.9-7.3)	<0.001	2.59 (2.18 to 3.00)
Obesity	4.1 (3.6-4.6)	5.4 (5.0-5.7)	6.1 (5.7-6.5)	6.6 (6.4-6.9)	6.7 (6.3-7.0)	6.7 (6.5-6.9)	6.6 (6.3-6.9)	6.4 (6.2-6.6)	6.8 (6.5-7.0)	6.9 (6.6-7.1)	<0.001	2.77 (2.24 to 3.31)
p for interaction											<0.001[c]	<0.001[d]
Saturated fats												
Total population	6.0 (5.7-6.3)	6.3 (6.1-6.4)	6.0 (5.8-6.2)	5.8 (5.6-6.0)	6.0 (5.8-6.2)	6.2 (6.1-6.4)	6.3 (6.1-6.5)	6.0 (5.8-6.2)	5.5 (5.3-5.7)	5.3 (5.1-5.5)	<0.001	-0.70 (-1.07 to -0.33)
Normal weight	6.3 (5.9-6.7)	6.4 (6.0-6.7)	6.3 (6.0-6.6)	5.9 (5.7-6.1)	6.2 (6.0-6.4)	6.5 (6.4-6.6)	6.6 (6.4-6.8)	6.5 (6.3-6.8)	5.6 (5.3-5.9)	5.6 (5.3-5.8)	<0.001	-0.71 (-1.21 to -0.21)
Overweight	6.2 (5.8-6.7)	6.3 (6.2-6.5)	6.1 (5.9-6.3)	5.9 (5.7-6.2)	6.1 (5.9-6.4)	6.2 (6.0-6.4)	6.3 (6.1-6.5)	5.8 (5.5-6.1)	5.5 (5.3-5.8)	5.4 (5.0-5.7)	<0.001	-0.86 (-1.41 to -0.31)
Obesity	5.5 (5.0-6.0)	6.1 (5.9-6.4)	5.6 (5.3-5.8)	5.6 (5.3-5.8)	5.7 (5.4-6.0)	6.0 (5.7-6.3)	6.0 (5.7-6.4)	5.8 (5.6-6.1)	5.3 (5.1-5.5)	5.1 (4.9-5.3)	<0.001	-0.39 (-0.93 to 0.16)
p for interaction											<0.001[c]	<0.001[d]

Abbreviations: HEI, Healthy Eating Index. [a] Normal weight is defined as a body mass index (BMI) ranging from 18.5 to 24.9, overweight is defined as a BMI ranging from 25 to 29.9, and obesity is defined as a BMI ≥ 30. [b] Survey-weighted mean scores were adjusted for age, sex, and race/ethnicity. [c] *p* for interaction assessing potential heterogeneous trends in components of the HEI-2020 diet score by body weight status. [d] *p* for interaction assessing potential heterogeneous changes in components of the HEI-2020 diet score from 1999-2000 to 2017-2020 by body weight status.

Table S5. Trends in Dietary Intake of Key Food Groups and Nutrients Among US Adults by Body Weight Status, 1999-2020[a].

**Dietary Intake**	**Survey-Weighted Mean Intake (95% CI)**										***p* for trend**	**Change From 1999-2020, Mean (95% CI)**
	**1999-2000 (*n* =1289)**	**2001-2002 (*n* =2774)**	**2003-2004 (*n* = 3053)**	**2005-2006 (*n* = 3204)**	**2007-2008 (*n* = 3731)**	**2009-2010 (*n* = 4012)**	**2011-2012 (*n* = 3382)**	**2013-2014 (*n* = 3876)**	**2015-2016 (*n* = 3786)**	**2017-2020 (*n* = 5522)**		
Total fruits, servings/d												
Total population	0.79 (0.63-0.94)	0.84 (0.80-0.89)	0.87 (0.77-0.97)	0.50 (0.47-0.54)	0.51 (0.48-0.55)	0.54 (0.53-0.56)	0.51 (0.48-0.55)	0.48 (0.46-0.50)	0.46 (0.41-0.50)	0.45 (0.42-0.48)	<0.001	-0.33 (-0.49 to -0.17)
Normal weight	0.86 (0.67-1.05)	0.94 (0.82-1.06)	0.87 (0.74-0.99)	0.53 (0.48-0.57)	0.53 (0.47-0.59)	0.59 (0.54-0.64)	0.57 (0.51-0.63)	0.51 (0.48-0.55)	0.50 (0.44-0.56)	0.46 (0.40-0.51)	<0.001	-0.41 (-0.60 to -0.21)
Overweight	0.76 (0.62-0.90)	0.83 (0.76-0.90)	0.92 (0.84-1.01)	0.52 (0.46-0.57)	0.53 (0.48-0.57)	0.56 (0.51-0.61)	0.56 (0.50-0.61)	0.49 (0.46-0.53)	0.46 (0.39-0.54)	0.47 (0.43-0.50)	<0.001	-0.29 (-0.44 to -0.15)
Obesity	0.72 (0.52-0.92)	0.74 (0.67-0.82)	0.82 (0.70-0.93)	0.47 (0.42-0.52)	0.48 (0.42-0.54)	0.50 (0.45-0.55)	0.42 (0.39-0.46)	0.44 (0.42-0.47)	0.42 (0.37-0.47)	0.44 (0.40-0.47)	<0.001	-0.29 (-0.49 to -0.08)
p for interaction											<0.001[b]	<0.001[c]
Whole fruits, servings/d												
Total population	0.43 (0.35-0.51)	0.48 (0.45-0.52)	0.52 (0.46-0.58)	0.33 (0.30-0.36)	0.36 (0.34-0.39)	0.39 (0.38-0.40)	0.37 (0.34-0.41)	0.36 (0.35-0.38)	0.34 (0.30-0.38)	0.35 (0.33-0.38)	<0.001	-0.08 (-0.16 to 0.01)
Normal weight	0.46 (0.35-0.58)	0.54 (0.47-0.60)	0.53 (0.45-0.61)	0.34 (0.29-0.39)	0.37 (0.33-0.42)	0.42 (0.38-0.45)	0.39 (0.34-0.44)	0.39 (0.36-0.43)	0.38 (0.33-0.44)	0.37 (0.32-0.41)	<0.001	-0.10 (-0.22 to 0.03)
Overweight	0.40 (0.35-0.45)	0.50 (0.45-0.54)	0.57 (0.51-0.63)	0.34 (0.30-0.38)	0.37 (0.33-0.42)	0.41 (0.37-0.45)	0.43 (0.37-0.49)	0.38 (0.34-0.41)	0.34 (0.29-0.39)	0.37 (0.34-0.40)	<0.001	-0.03 (-0.09 to 0.03)
Obesity	0.42 (0.29-0.55)	0.41 (0.36-0.47)	0.46 (0.38-0.54)	0.32 (0.28-0.36)	0.35 (0.30-0.39)	0.35 (0.32-0.39)	0.31 (0.27-0.34)	0.32 (0.30-0.35)	0.31 (0.26-0.36)	0.33 (0.30-0.36)	0.021	-0.09 (-0.22 to 0.04)
p for interaction											<0.001[b]	0.126[c]
Total vegetables, servings/d												
Total population	2.79 (2.56-3.01)	1.55 (1.48-1.62)	1.64 (1.56-1.72)	0.89 (0.85-0.93)	0.87 (0.84-0.89)	0.88 (0.85-0.90)	0.87 (0.83-0.91)	0.84 (0.80-0.87)	0.85 (0.82-0.89)	0.82 (0.79-0.86)	<0.001	-1.96 (-2.20 to -1.73)
Normal weight	2.78 (2.60-	1.62 (1.53-	1.62 (1.47-	0.90 (0.84-	0.87 (0.83-	0.88 (0.82-	0.87 (0.82-	0.84 (0.76-	0.89 (0.85-	0.86 (0.79-	<0.001	-1.92 (-2.11 to -1.73)

	2.96)	1.71)	1.77)	0.97)	0.92)	0.94)	0.93)	0.93)	0.92)	0.93)		
Overweight	2.74 (2.34-3.14)	1.56 (1.46-1.67)	1.70 (1.61-1.79)	0.84 (0.80-0.88)	0.85 (0.81-0.89)	0.88 (0.82-0.94)	0.91 (0.86-0.97)	0.85 (0.80-0.91)	0.82 (0.75-0.89)	0.85 (0.80-0.89)	<0.001	-1.89 (-2.30 to -1.49)
Obesity	2.84 (2.32-3.36)	1.45 (1.34-1.56)	1.60 (1.52-1.69)	0.93 (0.88-0.97)	0.88 (0.83-0.93)	0.87 (0.83-0.90)	0.83 (0.79-0.88)	0.81 (0.77-0.86)	0.86 (0.81-0.91)	0.78 (0.74-0.82)	<0.001	-2.06 (-2.58 to -1.54)
p for interaction											<0.001[b]	<0.001[c]
Greens and Beans, servings/d												
Total population	0.19 (0.16-0.22)	0.18 (0.16-0.20)	0.20 (0.18-0.22)	0.12 (0.11-0.14)	0.13 (0.11-0.14)	0.13 (0.12-0.14)	0.14 (0.12-0.15)	0.14 (0.13-0.15)	0.15 (0.13-0.16)	0.14 (0.12-0.15)	<0.001	-0.06 (-0.09 to -0.02)
Normal weight	0.20 (0.15-0.26)	0.20 (0.16-0.24)	0.20 (0.18-0.23)	0.14 (0.11-0.16)	0.14 (0.11-0.16)	0.14 (0.12-0.16)	0.15 (0.13-0.18)	0.16 (0.14-0.19)	0.17 (0.14-0.20)	0.16 (0.14-0.19)	0.310	-0.04 (-0.10 to 0.02)
Overweight	0.18 (0.13-0.24)	0.17 (0.15-0.20)	0.22 (0.18-0.25)	0.11 (0.10-0.13)	0.13 (0.10-0.15)	0.12 (0.10-0.14)	0.14 (0.12-0.17)	0.15 (0.13-0.18)	0.13 (0.11-0.16)	0.15 (0.13-0.17)	0.013	-0.04 (-0.10 to 0.02)
Obesity	0.19 (0.16-0.21)	0.16 (0.13-0.18)	0.18 (0.15-0.21)	0.12 (0.09-0.14)	0.12 (0.11-0.13)	0.12 (0.10-0.14)	0.11 (0.10-0.12)	0.12 (0.11-0.13)	0.14 (0.12-0.16)	0.11 (0.10-0.12)	<0.001	-0.08 (-0.11 to -0.05)
p for interaction											<0.001[b]	<0.001[c]
Whole grains, servings/d												
Total population	0.44 (0.35-0.52)	0.65 (0.60-0.70)	0.61 (0.55-0.68)	0.39 (0.35-0.42)	0.38 (0.35-0.41)	0.44 (0.42-0.47)	0.52 (0.47-0.57)	0.48 (0.45-0.51)	0.48 (0.45-0.52)	0.43 (0.40-0.47)	<0.001	0.00 (-0.09 to 0.09)
Normal weight	0.44 (0.32-0.56)	0.68 (0.56-0.79)	0.63 (0.53-0.74)	0.38 (0.33-0.44)	0.38 (0.34-0.41)	0.48 (0.44-0.53)	0.58 (0.50-0.66)	0.54 (0.49-0.60)	0.49 (0.44-0.54)	0.51 (0.43-0.58)	0.028	0.07 (-0.07 to 0.21)
Overweight	0.50 (0.35-0.66)	0.65 (0.56-0.75)	0.62 (0.54-0.70)	0.42 (0.37-0.47)	0.38 (0.34-0.43)	0.45 (0.41-0.48)	0.53 (0.45-0.61)	0.49 (0.43-0.55)	0.48 (0.41-0.54)	0.43 (0.38-0.48)	<0.001	-0.07 (-0.23 to 0.09)
Obesity	0.37 (0.29-0.44)	0.61 (0.49-0.73)	0.58 (0.52-0.65)	0.35 (0.32-0.39)	0.37 (0.34-0.41)	0.41 (0.38-0.45)	0.45 (0.40-0.50)	0.42 (0.39-0.46)	0.49 (0.45-0.53)	0.39 (0.36-0.43)	<0.001	0.03 (-0.05 to 0.11)
p for interaction											<0.001[b]	0.020[c]
Dairy, servings/d												
Total population	2.08 (1.93-2.23)	1.39 (1.32-1.46)	1.40 (1.32-1.47)	0.75 (0.72-0.78)	0.73 (0.70-0.77)	0.79 (0.77-0.81)	0.76 (0.73-0.79)	0.75 (0.71-0.78)	0.72 (0.68-0.75)	0.68 (0.65-0.71)	<0.001	-1.40 (-1.56 to -1.25)

Normal weight	2.14 (1.84-2.44)	1.50 (1.36-1.64)	1.45 (1.33-1.57)	0.75 (0.70-0.80)	0.75 (0.70-0.81)	0.79 (0.75-0.83)	0.78 (0.74-0.81)	0.78 (0.73-0.84)	0.74 (0.71-0.77)	0.64 (0.60-0.69)	<0.001	-1.49 (-1.80 to -1.19)
Overweight	1.90 (1.61-2.18)	1.42 (1.31-1.54)	1.35 (1.25-1.45)	0.77 (0.71-0.83)	0.73 (0.69-0.77)	0.80 (0.77-0.84)	0.75 (0.71-0.79)	0.74 (0.71-0.78)	0.74 (0.66-0.81)	0.68 (0.64-0.72)	<0.001	-1.22 (-1.51 to -0.93)
Obesity	2.20 (1.84-2.57)	1.24 (1.09-1.38)	1.40 (1.31-1.48)	0.74 (0.70-0.77)	0.72 (0.66-0.78)	0.78 (0.75-0.81)	0.76 (0.71-0.81)	0.72 (0.67-0.77)	0.69 (0.66-0.72)	0.70 (0.65-0.75)	<0.001	-1.51 (-1.88 to -1.14)
p for interaction											<0.001[b]	<0.001[c]
Total protein foods, servings/d												
Total population	9.96 (9.27-10.64)	5.62 (5.47-5.77)	5.96 (5.66-6.25)	3.17 (3.09-3.26)	3.20 (3.11-3.29)	3.26 (3.16-3.37)	3.15 (3.05-3.26)	3.25 (3.16-3.34)	3.27 (3.17-3.36)	3.28 (3.19-3.36)	<0.001	-6.68 (-7.37 to -5.99)
Normal weight	9.36 (7.84-10.88)	5.71 (5.17-6.25)	5.53 (5.22-5.85)	3.07 (2.95-3.20)	3.08 (2.92-3.24)	3.11 (2.97-3.25)	2.93 (2.79-3.07)	3.07 (2.90-3.24)	3.22 (3.09-3.36)	3.26 (3.07-3.44)	<0.001	-6.10 (-7.64 to -4.57)
Overweight	10.60 (9.42-11.79)	5.80 (5.55-6.05)	6.06 (5.79-6.33)	3.13 (3.03-3.24)	3.15 (3.01-3.28)	3.26 (3.10-3.41)	3.24 (3.13-3.35)	3.38 (3.27-3.50)	3.22 (3.10-3.33)	3.33 (3.17-3.48)	<0.001	-7.28 (-8.47 to -6.08)
Obesity	9.97 (8.94-11.00)	5.31 (5.04-5.57)	6.28 (5.84-6.72)	3.30 (3.17-3.44)	3.35 (3.22-3.48)	3.38 (3.26-3.51)	3.26 (3.09-3.43)	3.27 (3.16-3.38)	3.33 (3.21-3.46)	3.25 (3.14-3.36)	<0.001	-6.72 (-7.76 to -5.68)
p for interaction											<0.001[b]	<0.001[c]
Seafood and plant proteins, servings/d												
Total population	1.64 (1.32-1.96)	1.22 (1.12-1.32)	1.51 (1.37-1.64)	0.80 (0.73-0.86)	0.78 (0.71-0.86)	0.85 (0.79-0.92)	0.87 (0.80-0.94)	0.87 (0.80-0.94)	0.93 (0.86-1.01)	0.90 (0.84-0.96)	<0.001	-0.74 (-1.06 to -0.42)
Normal weight	1.78 (1.31-2.25)	1.26 (1.04-1.48)	1.50 (1.33-1.68)	0.87 (0.79-0.95)	0.83 (0.71-0.95)	0.86 (0.77-0.95)	0.91 (0.81-1.00)	0.94 (0.82-1.05)	1.06 (0.95-1.17)	1.00 (0.85-1.15)	<0.001	-0.78 (-1.27 to -0.29)
Overweight	1.20 (0.93-1.47)	1.28 (1.10-1.46)	1.62 (1.40-1.84)	0.81 (0.72-0.91)	0.80 (0.69-0.91)	0.89 (0.81-0.97)	0.91 (0.81-1.01)	0.92 (0.81-1.02)	0.91 (0.79-1.02)	0.98 (0.88-1.08)	<0.001	-0.21 (-0.50 to 0.07)
Obesity	1.94 (1.51-2.37)	1.11 (0.97-1.25)	1.39 (1.22-1.55)	0.71 (0.62-0.81)	0.73 (0.65-0.81)	0.82 (0.74-0.90)	0.80 (0.71-0.89)	0.79 (0.72-0.85)	0.88 (0.79-0.97)	0.79 (0.73-0.85)	<0.001	-1.15 (-1.58 to -0.72)
p for interaction											<0.001[b]	<0.001[c]
Fatty acids												
Total population	1.79 (1.75-	1.87 (1.84-	1.93 (1.91-	1.85 (1.83-	1.90 (1.87-	1.92 (1.90-	1.98 (1.95-	1.94 (1.91-	1.92 (1.87-	1.92 (1.89-	<0.001	0.13 (0.08 to 0.18)

	1.83)	1.89)	1.95)	1.88)	1.94)	1.95)	2.01)	1.97)	1.96)	1.95)		
Normal weight	1.79 (1.69-1.88)	1.84 (1.79-1.90)	1.95 (1.89-2.00)	1.86 (1.83-1.89)	1.89 (1.83-1.95)	1.92 (1.87-1.97)	2.01 (1.96-2.07)	1.95 (1.91-2.00)	1.91 (1.85-1.97)	1.95 (1.89-2.02)	0.052	0.17 (0.05 to 0.29)
Overweight	1.81 (1.72-1.90)	1.86 (1.82-1.90)	1.94 (1.90-1.99)	1.86 (1.81-1.91)	1.89 (1.85-1.94)	1.95 (1.91-1.98)	1.95 (1.91-2.00)	1.94 (1.88-2.00)	1.91 (1.83-1.99)	1.95 (1.89-2.00)	0.100	0.14 (0.03 to 0.24)
Obesity	1.77 (1.70-1.85)	1.90 (1.87-1.93)	1.90 (1.85-1.94)	1.85 (1.81-1.88)	1.92 (1.88-1.97)	1.90 (1.87-1.94)	1.98 (1.92-2.03)	1.92 (1.89-1.96)	1.92 (1.88-1.97)	1.88 (1.84-1.92)	0.055	0.11 (0.02 to 0.19)
p for interaction											<0.001[b]	0.094[c]
Refined grains, servings/d												
Total population	9.28 (8.75-9.82)	5.68 (5.47-5.89)	5.75 (5.61-5.88)	2.77 (2.70-2.83)	2.74 (2.67-2.81)	2.72 (2.65-2.79)	2.73 (2.65-2.80)	2.70 (2.64-2.76)	2.63 (2.54-2.71)	2.67 (2.61-2.73)	<0.001	-6.62 (-7.15 to -6.08)
Normal weight	9.52 (7.91-11.14)	5.48 (5.21-5.75)	5.70 (5.49-5.91)	2.71 (2.60-2.82)	2.74 (2.63-2.84)	2.65 (2.51-2.80)	2.68 (2.57-2.80)	2.66 (2.56-2.76)	2.53 (2.37-2.68)	2.64 (2.50-2.77)	<0.001	-6.89 (-8.51 to -5.27)
Overweight	9.09 (7.77-10.42)	5.83 (5.61-6.06)	5.76 (5.57-5.96)	2.71 (2.64-2.79)	2.76 (2.66-2.86)	2.69 (2.58-2.80)	2.67 (2.57-2.77)	2.63 (2.49-2.76)	2.68 (2.54-2.82)	2.66 (2.56-2.75)	<0.001	-6.44 (-7.76 to -5.11)
Obesity	9.21 (8.40-10.02)	5.71 (5.24-6.19)	5.78 (5.56-6.00)	2.87 (2.78-2.96)	2.72 (2.61-2.84)	2.80 (2.72-2.88)	2.82 (2.74-2.90)	2.80 (2.71-2.88)	2.65 (2.53-2.77)	2.69 (2.62-2.77)	<0.001	-6.51 (-7.33 to -5.70)
p for interaction											<0.001[b]	<0.001[c]
Sodium, g/d												
Total population	2.81 (2.69-2.94)	3.02 (2.95-3.09)	3.18 (3.10-3.26)	1.69 (1.66-1.72)	1.67 (1.66-1.69)	1.75 (1.72-1.78)	1.72 (1.70-1.74)	1.72 (1.69-1.75)	1.74 (1.71-1.77)	1.67 (1.64-1.69)	<0.001	-1.15 (-1.27 to -1.02)
Normal weight	2.82 (2.59-3.06)	2.99 (2.88-3.11)	3.13 (3.00-3.26)	1.66 (1.61-1.70)	1.67 (1.62-1.72)	1.71 (1.65-1.77)	1.68 (1.63-1.73)	1.68 (1.62-1.73)	1.73 (1.68-1.79)	1.63 (1.58-1.68)	<0.001	-1.19 (-1.43 to -0.95)
Overweight	2.76 (2.59-2.94)	3.09 (2.98-3.19)	3.16 (3.06-3.26)	1.64 (1.61-1.67)	1.65 (1.63-1.66)	1.74 (1.70-1.77)	1.73 (1.70-1.76)	1.73 (1.69-1.77)	1.71 (1.67-1.75)	1.64 (1.60-1.68)	<0.001	-1.12 (-1.30 to -0.94)
Obesity	2.85 (2.64-3.07)	2.96 (2.86-3.06)	3.25 (3.15-3.35)	1.76 (1.73-1.80)	1.70 (1.67-1.73)	1.80 (1.78-1.82)	1.74 (1.71-1.76)	1.74 (1.70-1.78)	1.76 (1.72-1.81)	1.70 (1.67-1.73)	<0.001	-1.15 (-1.37 to -0.93)
p for interaction											<0.001[b]	<0.001[c]
Added sugars, tsp equivalents/d, servings/d												

Total population	21.10 (19.97-22.24)	16.22 (15.35-17.10)	14.44 (13.50-15.37)	13.15 (12.52-13.77)	13.61 (12.78-14.44)	12.91 (12.55-13.28)	12.74 (12.24-13.23)	12.80 (12.19-13.40)	12.20 (11.68-12.72)	12.00 (11.48-12.51)	<0.001	-9.11 (-10.35 to -7.86)
Normal weight	21.48 (19.61-23.35)	15.90 (14.53-17.26)	14.88 (13.70-16.06)	13.32 (12.25-14.39)	13.75 (12.66-14.84)	13.57 (12.69-14.45)	13.10 (12.27-13.92)	13.37 (12.09-14.65)	11.85 (10.91-12.80)	11.79 (11.04-12.54)	<0.001	-9.69 (-11.70 to -7.67)
Overweight	20.55 (18.86-22.24)	16.09 (14.89-17.29)	13.94 (12.85-15.03)	13.31 (12.74-13.88)	13.97 (12.91-15.03)	12.53 (11.97-13.10)	12.16 (11.34-12.98)	11.45 (10.66-12.25)	11.98 (11.06-12.91)	11.71 (11.03-12.40)	<0.001	-8.84 (-10.66 to -7.02)
Obesity	21.24 (19.50-22.99)	16.74 (15.92-17.57)	14.51 (13.12-15.90)	12.83 (12.11-13.55)	13.14 (12.13-14.15)	12.76 (12.29-13.23)	12.96 (12.26-13.67)	13.56 (12.89-14.23)	12.59 (11.88-13.29)	12.33 (11.59-13.07)	<0.001	-8.92 (-10.81 to -7.02)
p for interaction											<0.001[b]	<0.001[c]
Saturated fats, % of energy												
Total population	11.00 (10.64-11.36)	10.76 (10.62-10.89)	11.04 (10.79-11.30)	11.27 (11.08-11.45)	11.02 (10.85-11.19)	10.78 (10.61-10.95)	10.69 (10.49-10.89)	10.98 (10.80-11.16)	11.58 (11.39-11.77)	11.74 (11.55-11.93)	<0.001	0.74 (0.34 to 1.14)
Normal weight	10.70 (10.23-11.17)	10.69 (10.31-11.08)	10.79 (10.47-11.12)	11.12 (10.90-11.34)	10.84 (10.60-11.07)	10.54 (10.40-10.68)	10.36 (10.13-10.59)	10.47 (10.24-10.69)	11.43 (11.14-11.72)	11.40 (11.14-11.66)	<0.001	0.70 (0.16 to 1.24)
Overweight	10.78 (10.34-11.22)	10.70 (10.53-10.88)	10.91 (10.60-11.21)	11.18 (10.90-11.45)	10.91 (10.66-11.16)	10.75 (10.50-11.00)	10.70 (10.48-10.92)	11.20 (10.91-11.49)	11.49 (11.18-11.79)	11.68 (11.31-12.04)	<0.001	0.90 (0.33 to 1.47)
Obesity	11.56 (10.98-12.14)	10.89 (10.59-11.19)	11.44 (11.17-11.72)	11.49 (11.24-11.74)	11.29 (10.96-11.62)	10.99 (10.69-11.30)	10.95 (10.58-11.32)	11.16 (10.89-11.44)	11.75 (11.53-11.97)	11.98 (11.76-12.20)	<0.001	0.42 (-0.20 to 1.04)
p for interaction											<0.001[b]	<0.001[c]
Energy, kcal												
Total population	1856.55	2012.44	2018.97	2059.72	2012.83	2031.94	2075.82	2035.46	2038.66	2017.43	0.007	160.88 (68.35 to 253.40)

	(1769.60-1943.51)	(1970.15-2054.73)	(1972.28-2065.67)	(2009.39-2110.06)	(1960.28-2065.37)	(2000.89-2062.99)	(2037.44-2114.19)	(2006.72-2064.21)	(2004.47-2072.84)	(1985.82-2049.04)		
Normal weight	1911.63 (1793.86-2029.40)	2022.42 (1924.10-2120.73)	1987.90 (1920.53-2055.26)	2063.90 (1990.98-2136.82)	2030.68 (1954.12-2107.23)	1978.14 (1928.21-2028.08)	2085.60 (2039.00-2132.20)	2030.50 (1944.40-2116.60)	2006.22 (1962.36-2050.08)	1982.36 (1926.20-2038.53)	0.053	70.73 (-59.74 to 201.21)
Overweight	1844.92 (1684.97-2004.88)	2077.62 (2012.25-2142.98)	2007.64 (1964.15-2051.13)	2107.05 (2042.49-2171.61)	1999.06 (1949.79-2048.32)	2111.34 (2039.47-2183.21)	2068.09 (1998.05-2138.14)	2064.86 (2007.98-2121.75)	2064.25 (2002.57-2125.93)	2018.42 (1960.60-2076.24)	0.406	173.49 (3.41 to 343.58)
Obesity	1807.19 (1674.08-1940.30)	1924.40 (1852.92-1995.89)	2062.61 (1981.09-2144.14)	2010.84 (1950.50-2071.18)	2011.26 (1933.44-2089.07)	2003.41 (1953.47-2053.35)	2074.60 (2004.27-2144.93)	2013.35 (1965.59-2061.11)	2039.02 (1978.24-2099.80)	2036.44 (1984.15-2088.73)	0.017	229.25 (86.24 to 372.26)
p for interaction											0.008[b]	0.015[c]
Total fat, % of energy												
Total population	32.08 (31.32-32.84)	33.47 (33.01-33.92)	33.88 (33.22-34.54)	33.83 (33.40-34.26)	33.75 (33.37-34.12)	33.19 (32.73-33.66)	33.16 (32.71-33.60)	34.07 (33.74-34.40)	35.51 (35.14-35.89)	36.35 (35.97-36.73)	<0.001	4.27 (3.41 to 5.12)
Normal weight	31.09 (30.19-31.99)	32.79 (32.03-33.55)	32.96 (32.19-33.73)	33.28 (32.71-33.85)	32.98 (32.44-33.52)	32.52 (31.99-33.05)	32.23 (31.75-32.70)	32.48 (31.94-33.02)	34.98 (34.20-35.76)	35.75 (35.05-36.44)	<0.001	4.66 (3.52 to 5.80)
Overweight	31.82 (31.08-32.56)	33.47 (32.95-34.00)	33.75 (32.79-34.70)	33.70 (33.11-34.29)	33.36 (32.93-33.80)	33.16 (32.54-33.77)	32.92 (32.38-33.46)	34.71 (34.22-35.20)	34.88 (34.42-35.34)	36.42 (35.72-37.12)	<0.001	4.60 (3.58 to 5.62)
Obesity	33.45 (32.15-34.75)	34.21 (33.31-35.12)	34.96 (34.07-35.85)	34.45 (33.90-34.99)	34.77 (33.97-35.56)	33.72 (32.93-34.52)	34.17 (33.43-34.92)	34.67 (34.06-35.28)	36.34 (35.76-36.92)	36.64 (36.20-37.07)	<0.001	3.19 (1.82 to 4.56)
p for interaction											<0.001[b]	<0.001[c]
Saturated fats, % of energy												
Total population	11.00 (10.64-	10.76 (10.62-	11.14 (10.90-	11.32 (11.14-	11.11 (10.93-	10.86 (10.68-	10.76 (10.56-	11.09 (10.91-	11.66 (11.47-	11.84 (11.66-	<0.001	0.84 (0.44 to 1.23)

	11.36)	10.89)	11.38)	11.50)	11.30)	11.04)	10.97)	11.27)	11.86)	12.01)		
Normal weight	10.70 (10.23-11.17)	10.69 (10.31-11.08)	10.84 (10.51-11.17)	11.19 (10.95-11.42)	10.90 (10.67-11.13)	10.65 (10.51-10.79)	10.44 (10.22-10.66)	10.54 (10.35-10.74)	11.49 (11.22-11.76)	11.54 (11.25-11.83)	<0.001	0.84 (0.29 to 1.39)
Overweight	10.78 (10.34-11.22)	10.70 (10.53-10.88)	11.04 (10.76-11.32)	11.24 (10.97-11.52)	11.01 (10.75-11.27)	10.82 (10.56-11.08)	10.76 (10.54-10.98)	11.34 (11.02-11.65)	11.55 (11.25-11.84)	11.76 (11.42-12.11)	<0.001	0.98 (0.42 to 1.54)
Obesity	11.56 (10.98-12.14)	10.89 (10.59-11.19)	11.54 (11.28-11.81)	11.52 (11.28-11.75)	11.40 (11.07-11.72)	11.06 (10.75-11.37)	11.05 (10.69-11.40)	11.27 (11.00-11.54)	11.86 (11.62-12.10)	12.06 (11.87-12.26)	<0.001	0.51 (-0.11 to 1.12)
p for interaction											<0.001[b]	<0.001[c]
Monounsaturated fat, % of energy												
	12.16 (11.83-12.49)	12.17 (12.00-12.34)	12.65 (12.40-12.90)	12.38 (12.23-12.54)	12.39 (12.24-12.55)	11.88 (11.69-12.06)	11.78 (11.58-11.99)	11.81 (11.71-11.92)	12.43 (12.27-12.59)	12.43 (12.27-12.58)	<0.001	0.27 (-0.09 to 0.63)
Normal weight	11.86 (11.44-12.27)	11.87 (11.59-12.16)	12.27 (12.00-12.55)	12.25 (12.04-12.46)	12.03 (11.84-12.23)	11.54 (11.31-11.78)	11.44 (11.19-11.68)	11.20 (10.95-11.44)	12.32 (12.00-12.64)	12.23 (11.90-12.56)	<0.001	0.37 (-0.15 to 0.90)
Overweight	11.97 (11.46-12.48)	12.15 (11.84-12.45)	12.58 (12.17-12.99)	12.31 (12.04-12.57)	12.27 (12.05-12.49)	11.87 (11.62-12.11)	11.69 (11.42-11.97)	12.07 (11.87-12.28)	12.22 (12.03-12.40)	12.57 (12.30-12.85)	<0.001	0.61 (0.03 to 1.19)
Obesity	12.68 (12.14-13.23)	12.54 (12.18-12.89)	13.10 (12.73-13.47)	12.58 (12.38-12.77)	12.81 (12.50-13.12)	12.13 (11.80-12.47)	12.16 (11.89-12.44)	12.03 (11.77-12.29)	12.67 (12.40-12.93)	12.43 (12.24-12.62)	0.015	-0.26 (-0.83 to 0.32)
p for interaction											<0.001[b]	0.089[c]
Polyunsaturated fat, % of energy												
Total population	6.43 (6.21-6.66)	6.66 (6.53-6.80)	7.22 (7.02-7.42)	7.16 (7.00-7.31)	7.28 (7.17-7.38)	7.38 (7.26-7.51)	7.86 (7.77-7.96)	7.89 (7.76-8.02)	8.15 (7.99-8.31)	8.43 (8.32-8.53)	<0.001	1.99 (1.75 to 2.24)
Normal weight	6.16 (5.83-	6.49 (6.26-	7.07 (6.76-	6.98 (6.80-	7.17 (6.90-	7.34 (7.08-	7.68 (7.51-	7.62 (7.39-	8.03 (7.69-	8.42 (8.17-	<0.001	2.26 (1.85 to 2.67)

	6.49)	6.71)	7.38)	7.15)	7.44)	7.61)	7.84)	7.86)	8.38)	8.67)		
Overweight	6.54 (6.18-6.91)	6.66 (6.50-6.81)	7.23 (6.92-7.55)	7.19 (6.89-7.48)	7.17 (6.96-7.38)	7.41 (7.22-7.60)	7.75 (7.60-7.91)	7.97 (7.75-8.19)	7.89 (7.62-8.16)	8.44 (8.29-8.59)	<0.001	1.89 (1.50 to 2.29)
Obesity	6.63 (6.21-7.04)	6.86 (6.61-7.11)	7.34 (6.96-7.73)	7.29 (7.11-7.47)	7.47 (7.26-7.67)	7.39 (7.23-7.54)	8.13 (7.94-8.31)	8.01 (7.84-8.17)	8.42 (8.20-8.64)	8.42 (8.25-8.60)	<0.001	1.80 (1.35 to 2.25)
p for interaction											<0.001[b]	<0.001[c]
Polyunsaturated: saturated fat ratio												
Total population	0.63 (0.61-0.66)	0.68 (0.66-0.69)	0.69 (0.68-0.71)	0.68 (0.66-0.69)	0.70 (0.68-0.71)	0.72 (0.71-0.74)	0.78 (0.76-0.79)	0.75 (0.74-0.77)	0.74 (0.72-0.76)	0.76 (0.74-0.77)	<0.001	0.12 (0.09 to 0.15)
Normal weight	0.63 (0.58-0.67)	0.67 (0.64-0.71)	0.71 (0.68-0.74)	0.67 (0.66-0.68)	0.70 (0.67-0.73)	0.73 (0.70-0.76)	0.79 (0.76-0.81)	0.77 (0.75-0.80)	0.74 (0.71-0.78)	0.77 (0.74-0.8)	<0.001	0.15 (0.09 to 0.21)
Overweight	0.66 (0.60-0.72)	0.67 (0.65-0.69)	0.70 (0.68-0.72)	0.68 (0.65-0.71)	0.69 (0.66-0.72)	0.74 (0.72-0.76)	0.77 (0.75-0.79)	0.74 (0.71-0.78)	0.73 (0.69-0.76)	0.77 (0.74-0.79)	<0.001	0.11 (0.05 to 0.18)
Obesity	0.62 (0.58-0.67)	0.69 (0.67-0.71)	0.67 (0.64-0.70)	0.67 (0.66-0.69)	0.70 (0.68-0.72)	0.71 (0.69-0.73)	0.78 (0.75-0.80)	0.75 (0.73-0.77)	0.75 (0.73-0.78)	0.74 (0.72-0.76)	<0.001	0.12 (0.07 to 0.17)
p for interaction											<0.001[b]	<0.001[c]
Protein, % of energy												
Total population	15.01 (14.77-15.25)	15.07 (14.87-15.27)	15.47 (15.16-15.78)	16.01 (15.78-16.23)	15.92 (15.70-16.13)	16.08 (15.75-16.40)	15.68 (15.45-15.91)	16.03 (15.79-16.27)	15.82 (15.55-16.09)	15.65 (15.44-15.86)	0.001	0.64 (0.32 to 0.96)
Normal weight	14.32 (13.88-14.76)	15.21 (14.92-15.50)	14.97 (14.65-15.30)	15.67 (15.39-15.96)	15.56 (15.07-16.05)	15.66 (15.26-16.06)	15.13 (14.79-15.47)	15.63 (15.14-16.11)	15.82 (15.45-16.19)	15.41 (15.00-15.83)	0.004	1.10 (0.49 to 1.70)
Overweight	15.54 (14.87-16.22)	15.07 (14.78-15.37)	15.68 (15.30-16.05)	15.89 (15.67-16.11)	15.81 (15.42-16.19)	16.06 (15.63-16.50)	15.93 (15.62-16.25)	16.36 (16.00-16.72)	15.79 (15.43-16.15)	15.69 (15.37-16.02)	0.054	0.15 (-0.60 to 0.90)
Obesity	15.25 (14.75-15.75)	14.92 (14.65-15.19)	15.76 (15.40-16.12)	16.42 (16.04-16.79)	16.33 (16.03-16.63)	16.39 (15.99-16.80)	15.92 (15.55-16.30)	16.04 (15.71-16.36)	15.83 (15.50-16.17)	15.76 (15.46-16.05)	0.001	0.51 (-0.08 to 1.09)

p for interaction											<0.001[b]	<0.001[c]
Carbohydrate, % of energy												
Total population	51.07 (50.23-51.91)	50.79 (50.07-51.51)	49.27 (48.48-50.07)	48.72 (48.12-49.31)	49.14 (48.57-49.70)	49.14 (48.65-49.63)	49.36 (48.77-49.95)	47.98 (47.35-48.60)	46.90 (46.40-47.40)	46.21 (45.67-46.74)	<0.001	-4.86 (-5.86 to -3.86)
Normal weight	52.40 (51.54-53.27)	51.02 (49.79-52.25)	50.48 (49.51-51.46)	48.99 (48.24-49.74)	49.84 (49.04-50.63)	50.33 (49.71-50.95)	50.74 (49.93-51.55)	49.77 (48.92-50.63)	47.07 (46.39-47.75)	46.78 (45.89-47.67)	<0.001	-5.62 (-6.86 to -4.38)
Overweight	50.35 (49.00-51.70)	50.31 (49.48-51.14)	49.31 (48.38-50.24)	48.77 (47.97-49.58)	49.55 (48.86-50.24)	48.67 (48.01-49.32)	48.71 (48.13-49.28)	46.13 (45.22-47.04)	47.17 (46.04-48.29)	46.28 (45.39-47.18)	<0.001	-4.07 (-5.69 to -2.45)
Obesity	50.31 (49.13-51.49)	51.11 (50.08-52.14)	48.00 (46.83-49.18)	48.42 (47.47-49.37)	48.14 (47.16-49.13)	48.67 (47.69-49.64)	48.78 (47.82-49.75)	48.29 (47.55-49.04)	46.58 (45.85-47.31)	45.82 (45.23-46.42)	<0.001	-4.48 (-5.80 to -3.16)
p for interaction											<0.001[b]	<0.001[c]
Cholesterol, mg/d												
Total population	208.67 (198.30-219.03)	254.40 (246.18-262.62)	274.54 (264.36-284.73)	279.00 (270.67-287.32)	275.90 (267.18-284.63)	269.31 (262.61-276.01)	275.02 (266.74-283.29)	281.81 (271.58-292.03)	287.34 (277.35-297.33)	295.58 (287.07-304.08)	<0.001	86.91 (73.50 to 100.32)
Normal weight	195.41 (182.41-208.41)	248.62 (226.14-271.09)	261.23 (246.42-276.05)	270.95 (255.55-286.35)	251.02 (239.52-262.51)	244.49 (234.03-254.95)	256.56 (239.39-273.73)	258.14 (247.44-268.83)	273.89 (260.77-287.02)	279.86 (267.45-292.26)	<0.001	84.44 (66.48 to 102.41)
Overweight	214.02 (197.77-230.27)	259.24 (248.36-270.12)	279.08 (265.20-292.96)	282.22 (270.57-293.86)	271.86 (263.14-280.57)	276.71 (264.63-288.79)	280.97 (263.51-298.43)	295.37 (278.79-311.95)	291.64 (277.78-305.49)	297.74 (279.89-315.59)	<0.001	83.72 (59.58 to 107.86)
Obesity	217.98 (196.75-239.20)	255.04 (237.67-272.41)	283.29 (264.61-301.96)	283.08 (270.68-295.47)	300.82 (283.88-317.76)	281.29 (269.02-293.57)	285.30 (270.14-300.45)	287.11 (273.48-300.75)	292.43 (276.98-307.88)	302.82 (292.89-312.76)	<0.001	84.85 (61.42 to 108.28)
p or interaction											<0.001[b]	<0.001[c]

Dietary fiber, g/d												
Total population	12.02 (11.28-12.76)	14.19 (13.58-14.80)	14.44 (13.68-15.20)	15.35 (14.79-15.92)	15.34 (14.53-16.16)	16.26 (15.94-16.58)	17.26 (16.79-17.73)	16.36 (16.04-16.69)	16.62 (15.87-17.37)	15.64 (15.14-16.14)	<0.001	3.62 (2.73 to 4.51)
Normal weight	12.68 (11.70-13.66)	14.60 (13.76-15.44)	14.56 (13.52-15.61)	15.36 (14.48-16.24)	15.89 (14.74-17.04)	16.50 (15.85-17.15)	18.05 (17.35-18.74)	16.91 (16.18-17.64)	17.07 (16.17-17.97)	16.21 (15.32-17.10)	<0.001	3.53 (2.21 to 4.85)
Overweight	11.68 (10.80-12.55)	14.41 (13.32-15.51)	14.61 (13.77-15.44)	15.76 (14.98-16.53)	15.24 (14.17-16.31)	16.93 (16.49-17.37)	17.51 (16.95-18.07)	16.67 (15.90-17.44)	16.69 (15.48-17.90)	16.26 (15.56-16.97)	<0.001	4.59 (3.46 to 5.71)
Obesity	11.62 (10.47-12.77)	13.48 (12.87-14.10)	14.13 (13.26-15.00)	14.96 (14.58-15.35)	14.99 (14.13-15.84)	15.51 (15.00-16.02)	16.37 (15.69-17.05)	15.69 (15.09-16.29)	16.29 (15.47-17.11)	14.84 (14.30-15.39)	<0.001	3.22 (1.95 to 4.50)
p for interaction											<0.001[b]	<0.001[c]
Potassium, mg/d												
Total population	2265.11 (2130.61-2399.61)	2467.33 (2410.03-2524.63)	2501.58 (2420.38-2582.79)	2629.83 (2551.66-2708.00)	2529.87 (2446.34-2613.40)	2645.31 (2612.51-2678.11)	2672.88 (2618.92-2726.84)	2553.47 (2501.03-2605.92)	2530.05 (2458.09-2602.01)	2467.73 (2407.46-2528.00)	<0.001	202.61 (55.23 to 350.00)
Normal weight	2344.36 (2215.57-2473.16)	2534.88 (2421.95-2647.80)	2473.80 (2356.78-2590.82)	2645.88 (2521.28-2770.49)	2583.17 (2450.29-2716.06)	2601.32 (2522.15-2680.48)	2709.52 (2646.88-2772.15)	2564.66 (2490.25-2639.06)	2566.94 (2473.13-2660.75)	2478.38 (2380.12-2576.65)	0.001	134.02 (-27.97 to 296.02)
Overweight	2290.13 (2118.33-2461.93)	2567.41 (2439.16-2695.66)	2543.38 (2452.41-2634.36)	2703.60 (2612.05-2795.16)	2504.94 (2400.99-2608.88)	2769.31 (2704.99-2833.63)	2710.25 (2610.02-2810.47)	2637.15 (2524.94-2749.36)	2565.43 (2446.61-2684.24)	2529.00 (2446.71-2611.28)	<0.001	238.87 (48.38 to 429.35)
Obesity	2152.13 (1923.17-2381.08)	2274.72 (2197.15-2352.30)	2485.59 (2393.04-2578.13)	2545.13 (2471.48-2618.78)	2509.37 (2401.05-2617.69)	2571.17 (2498.75-2643.58)	2606.96 (2499.67-2714.25)	2472.19 (2394.66-2549.71)	2479.18 (2387.78-2570.57)	2416.19 (2332.41-2499.98)	0.057	264.07 (20.26 to 507.87)
p for interaction											<0.001[b]	0.155[c]
Magnesium, mg/d												

Total population	234.41 (221.19-247.62)	248.55 (240.69-256.42)	255.91 (245.32-266.51)	284.79 (276.06-293.53)	275.85 (263.90-287.80)	289.06 (284.78-293.34)	295.83 (288.73-302.93)	286.61 (280.29-292.92)	289.74 (279.34-300.13)	284.33 (277.43-291.23)	<0.001	49.92 (35.01 to 64.83)
Normal weight	242.69 (227.07-258.32)	258.62 (245.96-271.27)	256.65 (242.62-270.67)	288.36 (275.73-300.99)	284.20 (268.29-300.11)	287.13 (278.46-295.79)	304.11 (295.23-312.99)	295.98 (284.12-307.84)	299.03 (287.65-310.40)	287.62 (276.39-298.85)	<0.001	44.93 (25.68 to 64.17)
Overweight	237.99 (220.20-255.78)	254.12 (239.57-268.68)	259.93 (247.31-272.54)	294.26 (282.87-305.66)	274.74 (257.93-291.55)	304.03 (294.50-313.55)	299.61 (289.07-310.15)	295.24 (282.78-307.70)	293.01 (277.89-308.14)	291.56 (281.42-301.71)	<0.001	53.57 (33.09 to 74.05)
Obesity	221.61 (201.77-241.46)	230.89 (222.30-239.49)	250.91 (240.28-261.53)	272.58 (264.95-280.21)	269.90 (256.71-283.08)	277.62 (269.96-285.27)	285.24 (274.30-296.19)	272.25 (265.42-279.09)	281.33 (269.94-292.72)	277.09 (268.81-285.37)	<0.001	55.48 (33.97 to 76.98)
p for interaction											<0.001[b]	<0.001[c]
Calcium, mg/d												
Total population	690.53 (645.81-735.25)	762.62 (736.48-788.76)	793.03 (762.90-823.16)	891.03 (861.66-920.41)	870.68 (834.80-906.55)	949.83 (934.68-964.98)	950.35 (925.03-975.68)	917.81 (899.37-936.26)	920.91 (886.29-955.52)	900.17 (881.37-918.98)	<0.001	209.65 (161.13 to 258.16)
Normal weight	724.80 (682.59-767.00)	803.62 (755.36-851.89)	811.97 (772.95-850.99)	886.51 (836.50-936.52)	891.14 (839.96-942.32)	925.79 (886.24-965.34)	974.05 (941.77-1006.33)	928.28 (891.61-964.94)	929.75 (895.40-964.09)	876.19 (848.16-904.22)	<0.001	151.39 (100.73 to 202.06)
Overweight	657.44 (597.99-716.89)	779.49 (741.06-817.93)	781.59 (735.09-828.10)	915.11 (876.59-953.63)	861.25 (829.32-893.17)	995.60 (958.22-1032.98)	938.45 (906.24-970.66)	927.06 (891.05-963.07)	941.80 (880.14-1003.46)	896.31 (873.96-918.66)	<0.001	238.87 (175.36 to 302.38)
Obesity	685.81 (601.66-769.96)	697.59 (647.56-747.62)	785.85 (752.10-819.60)	872.09 (844.00-900.17)	862.67 (808.38-916.95)	928.21 (906.18-950.25)	941.09 (891.50-990.68)	902.14 (864.71-939.57)	899.00 (862.64-935.37)	916.56 (873.36-959.76)	<0.001	230.75 (136.16 to 325.34)
p for interaction											<0.001[b]	<0.001[c]

[a] Normal weight is defined as a body mass index (BMI) ranging from 18.5 to 24.9, overweight is defined as a BMI ranging from 25 to 29.9, and obesity is defined as a BMI ≥ 30. [b] *p* for interaction assessing potential heterogeneous trends in dietary intake of key food groups and nutrients by body weight status. [c] *p* for interaction assessing potential heterogeneous changes in dietary intake of

key food groups and nutrients from 1999-2000 to 2017-2020 by body weight status.

Table S6. Adjusted Trends in Dietary Intake of Key Food Groups and Nutrients Among US Adults by Body Weight Status, 1999-2020[a].

Dietary Intake	Survey-Weighted Mean Score (95% CI)[b]										*P* for Trend	Change From 1999-2020, Mean (95% CI)
	1999-2000 (*n* =1289)	2001-2002 (*n* =2774)	2003-2004 (*n* = 3053)	2005-2006 (*n* = 3204)	2007-2008 (*n* = 3731)	2009-2010 (*n* = 4012)	2011-2012 (*n* = 3382)	2013-2014 (*n* = 3876)	2015-2016 (*n* = 3786)	2017-2020 (*n* = 5522)		
Total fruits, servings/d												
Total population	0.80 (0.65-0.96)	0.85 (0.80-0.90)	0.87 (0.77-0.97)	0.51 (0.47-0.54)	0.52 (0.48-0.56)	0.54 (0.52-0.56)	0.51 (0.48-0.55)	0.48 (0.46-0.50)	0.45 (0.41-0.49)	0.45 (0.42-0.47)	<0.001	-0.36 (-0.51 to -0.20)
Normal weight	0.88 (0.69-1.06)	0.95 (0.83-1.07)	0.86 (0.73-0.99)	0.53 (0.49-0.57)	0.53 (0.47-0.59)	0.59 (0.54-0.64)	0.57 (0.51-0.63)	0.51 (0.47-0.55)	0.50 (0.44-0.55)	0.45 (0.41-0.50)	<0.001	-0.42 (-0.61 to -0.23)
Overweight	0.77 (0.63-0.92)	0.84 (0.77-0.90)	0.92 (0.83-1.01)	0.52 (0.46-0.58)	0.53 (0.48-0.58)	0.56 (0.51-0.61)	0.56 (0.49-0.62)	0.50 (0.46-0.53)	0.46 (0.39-0.54)	0.45 (0.42-0.49)	<0.001	-0.32 (-0.47 to -0.17)
Obesity	0.74 (0.56-0.93)	0.74 (0.67-0.82)	0.83 (0.71-0.94)	0.47 (0.43-0.51)	0.49 (0.43-0.55)	0.50 (0.45-0.54)	0.42 (0.39-0.46)	0.44 (0.41-0.47)	0.42 (0.37-0.47)	0.43 (0.40-0.47)	<0.001	-0.31 (-0.50 to -0.12)
p for interaction											<0.001[c]	<0.001[d]
Whole fruits, servings/d												
Total population	0.44 (0.36-0.53)	0.49 (0.45-0.53)	0.52 (0.46-0.58)	0.33 (0.31-0.36)	0.37 (0.33-0.40)	0.39 (0.38-0.40)	0.37 (0.34-0.41)	0.36 (0.34-0.38)	0.34 (0.30-0.37)	0.35 (0.32-0.37)	<0.001	-0.10 (-0.18 to -0.01)
Normal weight	0.48 (0.36-0.60)	0.55 (0.49-0.61)	0.53 (0.45-0.61)	0.34 (0.30-0.38)	0.37 (0.33-0.42)	0.42 (0.38-0.45)	0.39 (0.34-0.45)	0.39 (0.35-0.43)	0.38 (0.33-0.43)	0.36 (0.33-0.40)	<0.001	-0.11 (-0.24 to 0.01)
Overweight	0.41 (0.36-0.46)	0.50 (0.46-0.55)	0.57 (0.51-0.63)	0.35 (0.31-0.39)	0.37 (0.32-0.42)	0.41 (0.37-0.45)	0.43 (0.37-0.49)	0.38 (0.34-0.41)	0.34 (0.29-0.39)	0.36 (0.32-0.39)	<0.001	-0.06 (-0.12 to 0.00)
Obesity	0.44 (0.32-0.56)	0.42 (0.36-0.47)	0.47 (0.40-0.54)	0.32 (0.28-0.35)	0.35 (0.31-0.39)	0.35 (0.31-0.39)	0.31 (0.28-0.33)	0.32 (0.30-0.34)	0.31 (0.26-0.35)	0.33 (0.30-0.36)	<0.001	-0.11 (-0.24 to 0.02)
p for interaction											<0.001[c]	<0.001[d]
Total vegetables, servings/d												
Total population	2.80 (2.57-3.02)	1.55 (1.48-1.62)	1.64 (1.56-1.72)	0.89 (0.86-0.93)	0.87 (0.84-0.89)	0.88 (0.85-0.90)	0.87 (0.83-0.91)	0.83 (0.80-0.87)	0.85 (0.82-0.88)	0.82 (0.78-0.85)	<0.001	-1.98 (-2.21 to -1.75)
Normal weight	2.79 (2.61-2.96)	1.63 (1.54-1.71)	1.62 (1.47-1.76)	0.90 (0.85-0.96)	0.87 (0.82-0.92)	0.88 (0.82-0.93)	0.87 (0.82-0.93)	0.84 (0.75-0.93)	0.89 (0.85-0.92)	0.86 (0.79-0.93)	<0.001	-1.93 (-2.11 to -1.74)

Overweight	2.75 (2.34-3.15)	1.57 (1.46-1.67)	1.69 (1.61-1.78)	0.84 (0.80-0.89)	0.85 (0.81-0.89)	0.88 (0.82-0.94)	0.92 (0.86-0.97)	0.85 (0.80-0.91)	0.82 (0.75-0.89)	0.84 (0.79-0.89)	<0.001	-1.91 (-2.31 to -1.50)
Obesity	2.86 (2.34-3.37)	1.45 (1.34-1.57)	1.61 (1.53-1.69)	0.93 (0.88-0.97)	0.88 (0.83-0.93)	0.87 (0.83-0.90)	0.83 (0.79-0.88)	0.81 (0.77-0.85)	0.86 (0.81-0.91)	0.78 (0.74-0.82)	<0.001	-2.08 (-2.59 to -1.56)
p for interaction											<0.001[c]	<0.001[d]
Greens and Beans, servings/d												
Total population	0.19 (0.16-0.22)	0.18 (0.15-0.2)	0.20 (0.18-0.22)	0.12 (0.11-0.14)	0.13 (0.11-0.14)	0.13 (0.12-0.14)	0.14 (0.12-0.15)	0.14 (0.13-0.16)	0.15 (0.13-0.16)	0.14 (0.12-0.15)	<0.001	-0.06 (-0.09 to -0.02)
Normal weight	0.20 (0.15-0.26)	0.20 (0.16-0.24)	0.20 (0.18-0.23)	0.14 (0.11-0.16)	0.13 (0.11-0.16)	0.14 (0.12-0.16)	0.16 (0.13-0.18)	0.16 (0.14-0.19)	0.17 (0.14-0.20)	0.16 (0.14-0.19)	<0.001	-0.04 (-0.10 to 0.02)
Overweight	0.18 (0.13-0.24)	0.17 (0.15-0.20)	0.22 (0.18-0.25)	0.12 (0.10-0.13)	0.12 (0.10-0.15)	0.12 (0.10-0.14)	0.15 (0.12-0.17)	0.15 (0.13-0.18)	0.13 (0.11-0.16)	0.15 (0.13-0.17)	0.001	-0.04 (-0.10 to 0.03)
Obesity	0.19 (0.16-0.22)	0.16 (0.13-0.18)	0.18 (0.15-0.21)	0.12 (0.10-0.14)	0.12 (0.11-0.13)	0.12 (0.10-0.14)	0.11 (0.10-0.12)	0.12 (0.11-0.13)	0.14 (0.12-0.16)	0.11 (0.10-0.12)	<0.001	-0.08 (-0.11 to -0.05)
p for interaction											<0.001[c]	<0.001[d]
Whole grains, servings/d												
Total population	0.45 (0.37-0.53)	0.66 (0.60-0.71)	0.61 (0.55-0.68)	0.39 (0.36-0.42)	0.38 (0.35-0.41)	0.44 (0.42-0.47)	0.52 (0.47-0.56)	0.48 (0.45-0.50)	0.48 (0.45-0.51)	0.43 (0.39-0.46)	<0.001	-0.03 (-0.12 to 0.06)
Normal weight	0.45 (0.34-0.57)	0.69 (0.57-0.80)	0.63 (0.53-0.73)	0.39 (0.34-0.44)	0.38 (0.34-0.41)	0.48 (0.44-0.53)	0.58 (0.51-0.65)	0.54 (0.49-0.59)	0.48 (0.44-0.53)	0.50 (0.43-0.58)	<0.001	0.05 (-0.09 to 0.19)
Overweight	0.52 (0.37-0.68)	0.66 (0.57-0.76)	0.62 (0.54-0.70)	0.42 (0.37-0.47)	0.39 (0.34-0.44)	0.45 (0.41-0.49)	0.53 (0.45-0.61)	0.49 (0.43-0.55)	0.47 (0.41-0.53)	0.42 (0.37-0.46)	<0.001	-0.11 (-0.27 to 0.06)
Obesity	0.38 (0.31-0.45)	0.62 (0.50-0.73)	0.59 (0.52-0.65)	0.35 (0.32-0.38)	0.38 (0.35-0.41)	0.41 (0.37-0.45)	0.45 (0.41-0.50)	0.42 (0.39-0.45)	0.48 (0.45-0.52)	0.39 (0.36-0.42)	<0.001	0.01 (-0.07 to 0.09)
p for interaction											<0.001[c]	<0.001[d]
Dairy, servings/d												
Total population	2.08 (1.93-2.23)	1.39 (1.32-1.45)	1.40 (1.32-1.47)	0.75 (0.72-0.78)	0.73 (0.69-0.77)	0.79 (0.77-0.81)	0.76 (0.73-0.8)	0.75 (0.71-0.78)	0.72 (0.68-0.76)	0.68 (0.65-0.71)	<0.001	-1.39 (-1.55 to -1.24)

Normal weight	2.13 (1.83-2.43)	1.49 (1.35-1.63)	1.45 (1.33-1.57)	0.75 (0.70-0.79)	0.75 (0.69-0.81)	0.79 (0.75-0.83)	0.78 (0.74-0.82)	0.79 (0.73-0.84)	0.75 (0.72-0.78)	0.65 (0.61-0.70)	<0.001	-1.48 (-1.78 to -1.18)
Overweight	1.89 (1.61-2.17)	1.42 (1.30-1.54)	1.35 (1.25-1.45)	0.77 (0.71-0.83)	0.73 (0.69-0.77)	0.80 (0.76-0.84)	0.75 (0.72-0.79)	0.74 (0.71-0.78)	0.74 (0.66-0.81)	0.69 (0.65-0.73)	<0.001	-1.21 (-1.49 to -0.92)
Obesity	2.20 (1.84-2.56)	1.23 (1.09-1.37)	1.39 (1.30-1.48)	0.74 (0.70-0.78)	0.72 (0.66-0.78)	0.78 (0.75-0.81)	0.76 (0.71-0.81)	0.72 (0.67-0.77)	0.69 (0.66-0.72)	0.70 (0.65-0.75)	<0.001	-1.50 (-1.87 to -1.13)
p for interaction											<0.001[c]	<0.001[d]
Total protein foods, servings/d												
Total population	9.96 (9.27-10.65)	5.62 (5.47-5.77)	5.96 (5.68-6.24)	3.17 (3.08-3.26)	3.21 (3.13-3.29)	3.26 (3.16-3.37)	3.15 (3.05-3.25)	3.25 (3.16-3.34)	3.26 (3.17-3.36)	3.28 (3.19-3.36)	<0.001	-6.69 (-7.38 to -5.99)
Normal weight	9.36 (7.87-10.84)	5.71 (5.19-6.23)	5.55 (5.26-5.85)	3.09 (2.95-3.23)	3.09 (2.93-3.26)	3.13 (2.98-3.27)	2.91 (2.78-3.04)	3.06 (2.90-3.22)	3.20 (3.06-3.33)	3.24 (3.04-3.43)	<0.001	-6.12 (-7.62 to -4.62)
Overweight	10.59 (9.39-11.78)	5.78 (5.54-6.03)	6.08 (5.83-6.33)	3.11 (3.00-3.22)	3.15 (3.04-3.26)	3.25 (3.10-3.41)	3.25 (3.14-3.35)	3.37 (3.25-3.49)	3.22 (3.10-3.34)	3.36 (3.19-3.52)	<0.001	-7.23 (-8.43 to -6.03)
Obesity	10.00 (8.95-11.04)	5.34 (5.07-5.60)	6.27 (5.84-6.69)	3.31 (3.18-3.44)	3.36 (3.23-3.49)	3.37 (3.25-3.50)	3.25 (3.09-3.41)	3.28 (3.18-3.38)	3.33 (3.19-3.48)	3.24 (3.13-3.35)	<0.001	-6.76 (-7.80 to -5.71)
p for interaction											<0.001[c]	<0.001[d]
Seafood and plant proteins, servings/d												
Total population	1.66 (1.34-1.98)	1.22 (1.12-1.33)	1.51 (1.37-1.64)	0.80 (0.73-0.86)	0.79 (0.71-0.86)	0.85 (0.79-0.92)	0.87 (0.80-0.94)	0.87 (0.80-0.94)	0.93 (0.86-1.01)	0.90 (0.84-0.96)	<0.001	-0.76 (-1.08 to -0.43)
Normal weight	1.79 (1.32-2.27)	1.27 (1.05-1.49)	1.51 (1.34-1.67)	0.88 (0.79-0.96)	0.83 (0.72-0.95)	0.86 (0.77-0.95)	0.90 (0.81-1.00)	0.93 (0.82-1.04)	1.05 (0.94-1.16)	0.99 (0.85-1.14)	<0.001	-0.80 (-1.30 to -0.30)
Overweight	1.21 (0.94-1.48)	1.28 (1.10-1.46)	1.62 (1.40-1.84)	0.81 (0.72-0.91)	0.80 (0.69-0.91)	0.89 (0.81-0.97)	0.91 (0.81-1.01)	0.91 (0.81-1.02)	0.90 (0.79-1.02)	0.98 (0.88-1.08)	<0.001	-0.23 (-0.52 to 0.06)
Obesity	1.96 (1.54-2.38)	1.12 (0.97-1.26)	1.39 (1.23-1.55)	0.71 (0.62-0.81)	0.74 (0.66-0.81)	0.81 (0.73-0.89)	0.80 (0.71-0.89)	0.78 (0.72-0.85)	0.87 (0.79-0.96)	0.79 (0.73-0.85)	<0.001	-1.17 (-1.60 to -0.75)
p for interaction											<0.001[c]	<0.001[d]
Fatty acids												
Total population	1.79 (1.75-	1.87 (1.85-	1.93 (1.91-	1.85 (1.83-	1.91 (1.87-	1.92 (1.90-	1.98 (1.94-	1.94 (1.91-	1.91 (1.87-	1.92 (1.89-	<0.001	0.13 (0.08 to 0.17)

	1.83)	1.89)	1.95)	1.88)	1.94)	1.95)	2.01)	1.97)	1.96)	1.95)		
Normal weight	1.79 (1.69-1.89)	1.85 (1.80-1.90)	1.95 (1.89-2.01)	1.86 (1.84-1.89)	1.89 (1.84-1.95)	1.92 (1.87-1.97)	2.01 (1.95-2.07)	1.95 (1.91-2.00)	1.90 (1.84-1.96)	1.95 (1.88-2.01)	<0.001	0.16 (0.04 to 0.28)
Overweight	1.82 (1.73-1.90)	1.86 (1.82-1.90)	1.94 (1.89-1.99)	1.86 (1.81-1.91)	1.90 (1.85-1.94)	1.95 (1.91-1.98)	1.95 (1.91-2.00)	1.94 (1.88-2.00)	1.91 (1.83-1.99)	1.94 (1.88-2.00)	<0.001	0.12 (0.02 to 0.23)
Obesity	1.77 (1.70-1.85)	1.90 (1.87-1.93)	1.90 (1.85-1.94)	1.84 (1.81-1.88)	1.93 (1.88-1.97)	1.90 (1.86-1.94)	1.98 (1.92-2.03)	1.92 (1.89-1.96)	1.92 (1.88-1.96)	1.88 (1.84-1.92)	0.014	0.11 (0.02 to 0.19)
p for interaction											<0.001[c]	0.007[d]
Refined grains, servings/d												
Total population	9.24 (8.72-9.76)	5.65 (5.45-5.85)	5.74 (5.62-5.87)	2.76 (2.69-2.83)	2.73 (2.66-2.79)	2.72 (2.66-2.79)	2.73 (2.66-2.80)	2.71 (2.65-2.77)	2.64 (2.57-2.71)	2.69 (2.64-2.75)	<0.001	-6.55 (-7.07 to -6.02)
Normal weight	9.48 (7.88-11.08)	5.44 (5.18-5.71)	5.71 (5.50-5.93)	2.71 (2.59-2.83)	2.74 (2.64-2.83)	2.66 (2.54-2.77)	2.68 (2.57-2.80)	2.67 (2.57-2.78)	2.54 (2.40-2.68)	2.64 (2.50-2.77)	<0.001	-6.84 (-8.44 to -5.24)
Overweight	9.03 (7.72-10.35)	5.81 (5.61-6.02)	5.77 (5.57-5.97)	2.70 (2.62-2.79)	2.74 (2.66-2.82)	2.68 (2.57-2.78)	2.68 (2.58-2.77)	2.62 (2.49-2.76)	2.69 (2.56-2.82)	2.71 (2.60-2.81)	<0.001	-6.33 (-7.65 to -5.01)
Obesity	9.18 (8.39-9.97)	5.70 (5.26-6.15)	5.76 (5.56-5.97)	2.88 (2.80-2.97)	2.70 (2.60-2.81)	2.81 (2.73-2.89)	2.80 (2.74-2.87)	2.81 (2.72-2.89)	2.66 (2.57-2.75)	2.70 (2.65-2.76)	<0.001	-6.48 (-7.27 to -5.68)
p for interaction											<0.001[c]	<0.001[d]
Sodium, g/d												
Total population	2.81 (2.69-2.93)	3.02 (2.95-3.09)	3.18 (3.10-3.26)	1.69 (1.66-1.72)	1.67 (1.66-1.69)	1.75 (1.73-1.78)	1.72 (1.70-1.73)	1.72 (1.69-1.75)	1.74 (1.71-1.77)	1.67 (1.64-1.69)	<0.001	-1.14 (-1.26 to -1.02)
Normal weight	2.81 (2.59-3.04)	3.00 (2.89-3.11)	3.14 (3.00-3.27)	1.66 (1.61-1.72)	1.67 (1.63-1.72)	1.72 (1.67-1.77)	1.67 (1.63-1.71)	1.67 (1.62-1.73)	1.73 (1.67-1.78)	1.62 (1.58-1.67)	<0.001	-1.19 (-1.42 to -0.96)
Overweight	2.76 (2.58-2.93)	3.08 (2.98-3.18)	3.17 (3.07-3.27)	1.63 (1.60-1.67)	1.65 (1.63-1.66)	1.73 (1.70-1.77)	1.73 (1.70-1.76)	1.73 (1.69-1.76)	1.71 (1.67-1.75)	1.65 (1.61-1.69)	<0.001	-1.11 (-1.29 to -0.93)
Obesity	2.85 (2.63-3.07)	2.97 (2.87-3.07)	3.25 (3.15-3.35)	1.76 (1.73-1.80)	1.70 (1.68-1.73)	1.80 (1.78-1.82)	1.74 (1.71-1.76)	1.75 (1.71-1.79)	1.76 (1.72-1.81)	1.70 (1.67-1.73)	<0.001	-1.15 (-1.38 to -0.93)
p for interaction											<0.001[c]	<0.001[d]
Added sugars, tsp equivalents/d, servings/d												

Total population	20.86 (19.76-21.95)	16.12 (15.27-16.98)	14.43 (13.55-15.3)	13.14 (12.57-13.71)	13.56 (12.71-14.41)	12.92 (12.51-13.34)	12.74 (12.23-13.26)	12.84 (12.22-13.47)	12.26 (11.74-12.78)	12.08 (11.55-12.61)	<0.001	-8.78 (-9.99 to -7.56)
Normal weight	21.29 (19.40-23.19)	15.74 (14.36-17.13)	14.89 (13.72-16.06)	13.27 (12.28-14.26)	13.73 (12.67-14.78)	13.55 (12.64-14.46)	13.16 (12.26-14.06)	13.43 (12.18-14.68)	11.97 (11.05-12.89)	11.85 (11.08-12.61)	<0.001	-9.45 (-11.49 to -7.40)
Overweight	20.39 (18.77-22.01)	16.03 (14.84-17.23)	13.97 (12.88-15.07)	13.30 (12.75-13.85)	13.92 (12.82-15.02)	12.50 (11.90-13.10)	12.15 (11.32-12.99)	11.46 (10.64-12.27)	12.03 (11.05-13.01)	11.83 (11.14-12.51)	<0.001	-8.56 (-10.32 to -6.80)
Obesity	20.81 (19.26-22.36)	16.58 (15.71-17.46)	14.39 (13.15-15.63)	12.84 (12.22-13.45)	13.07 (12.05-14.09)	12.86 (12.46-13.26)	12.98 (12.30-13.67)	13.64 (13.00-14.28)	12.66 (11.96-13.37)	12.39 (11.65-13.13)	<0.001	-8.42 (-10.14 to -6.70)
p for interaction											<0.001[c]	<0.001[d]
Saturated fats, % of energy												
Total population	11.02 (10.66-11.37)	10.76 (10.63-10.89)	11.04 (10.79-11.30)	11.27 (11.08-11.45)	11.02 (10.85-11.19)	10.78 (10.61-10.95)	10.69 (10.49-10.89)	10.98 (10.80-11.16)	11.58 (11.40-11.76)	11.74 (11.56-11.92)	<0.001	0.72 (0.32 to 1.12)
Normal weight	10.69 (10.23-11.15)	10.66 (10.29-11.03)	10.77 (10.44-11.10)	11.10 (10.87-11.33)	10.83 (10.59-11.06)	10.54 (10.39-10.68)	10.38 (10.14-10.62)	10.48 (10.25-10.71)	11.47 (11.19-11.75)	11.43 (11.15-11.70)	<0.001	0.73 (0.20 to 1.27)
Overweight	10.79 (10.35-11.23)	10.71 (10.53-10.88)	10.90 (10.60-11.21)	11.18 (10.90-11.46)	10.91 (10.66-11.16)	10.75 (10.50-11.00)	10.70 (10.48-10.92)	11.20 (10.91-11.49)	11.48 (11.19-11.78)	11.67 (11.31-12.04)	<0.001	0.89 (0.32 to 1.46)
Obesity	11.59 (11.01-12.16)	10.90 (10.60-11.20)	11.45 (11.19-11.72)	11.48 (11.23-11.73)	11.30 (10.97-11.63)	10.98 (10.68-11.28)	10.96 (10.59-11.32)	11.16 (10.88-11.43)	11.74 (11.52-11.97)	11.97 (11.76-12.19)	<0.001	0.39 (-0.23 to 1.00)
p for interaction											<0.001[c]	<0.001[d]
Energy, kcal												
Total population	1848.60 (1766.65-1930.54)	2009.08 (1971.44-2046.72)	2022.80 (1989.29-2056.32)	2056.31 (2015.04-2097.58)	2016.33 (1963.61-2069.04)	2032.33 (1996.21-2068.44)	2071.94 (2029.85-2114.02)	2034.75 (2004.69-2064.81)	2040.20 (2007.35-2073.04)	2021.60 (1989.09-2054.12)	<0.001	173.00 (84.90 to 261.11)
Normal weight	1901.26 (1799.96-2002.57)	2010.12 (1916.82-2103.43)	1999.95 (1943.07-2056.84)	2073.22 (2015.85-2130.59)	2036.77 (1960.53-2113.00)	1991.00 (1956.93-2025.08)	2074.62 (2029.69-2119.55)	2030.29 (1945.20-2115.38)	1999.27 (1947.83-2050.71)	1974.03 (1920.98-2027.08)	<0.001	72.77 (-41.77 to 187.31)
Overweight	1825.72 (1679.69-1971.75)	2060.98 (2005.61-2116.35)	2026.91 (1996.18-2057.63)	2080.10 (2019.75-2140.44)	2002.52 (1956.34-2048.70)	2108.58 (2027.29-2189.87)	2073.82 (2009.27-2138.36)	2049.80 (1997.47-2102.13)	2068.27 (2002.58-2133.96)	2051.23 (1989.65-2112.81)	<0.001	225.51 (67.13 to 383.89)

Obesity	1809.62 (1668.98-1950.26)	1946.29 (1888.47-2004.10)	2042.54 (1975.53-2109.55)	2015.98 (1966.21-2065.74)	2013.80 (1939.37-2088.23)	1997.66 (1953.72-2041.59)	2068.48 (1993.23-2143.72)	2024.20 (1981.09-2067.30)	2044.09 (2003.28-2084.91)	2027.61 (1975.13-2080.10)	<0.001	217.99 (67.91 to 368.07)
p for interaction											<0.001[c]	<0.001[d]
Total fat, % of energy												
Total population	32.16 (31.42-32.91)	33.51 (33.07-33.94)	33.88 (33.24-34.53)	33.84 (33.40-34.27)	33.77 (33.41-34.13)	33.19 (32.74-33.64)	33.15 (32.70-33.61)	34.06 (33.73-34.38)	35.49 (35.13-35.85)	36.32 (35.93-36.70)	<0.001	4.15 (3.32 to 4.99)
Normal weight	31.10 (30.21-31.98)	32.77 (32.02-33.51)	32.92 (32.15-33.70)	33.25 (32.69-33.82)	32.97 (32.42-33.51)	32.51 (31.98-33.04)	32.25 (31.74-32.76)	32.49 (31.94-33.04)	35.02 (34.25-35.80)	35.78 (35.07-36.49)	<0.001	4.68 (3.55 to 5.82)
Overweight	31.90 (31.13-32.67)	33.50 (32.97-34.03)	33.74 (32.80-34.67)	33.71 (33.10-34.32)	33.39 (32.95-33.83)	33.18 (32.58-33.77)	32.91 (32.36-33.45)	34.71 (34.22-35.20)	34.86 (34.44-35.29)	36.35 (35.66-37.04)	<0.001	4.45 (3.42 to 5.48)
Obesity	33.55 (32.28-34.82)	34.27 (33.41-35.13)	35.00 (34.12-35.87)	34.42 (33.86-34.98)	34.81 (34.06-35.57)	33.68 (32.91-34.46)	34.20 (33.47-34.92)	34.65 (34.05-35.24)	36.31 (35.76-36.87)	36.61 (36.16-37.05)	<0.001	3.05 (1.71 to 4.40)
p for interaction											<0.001[c]	<0.001[d]
Saturated fats, % of energy												
Total population	11.02 (10.66-11.37)	10.76 (10.63-10.89)	11.14 (10.89-11.38)	11.32 (11.14-11.50)	11.11 (10.93-11.30)	10.86 (10.68-11.04)	10.77 (10.56-10.97)	11.09 (10.91-11.27)	11.66 (11.47-11.85)	11.84 (11.66-12.01)	<0.001	0.82 (0.43 to 1.21)
Normal weight	10.69 (10.23-11.15)	10.66 (10.29-11.03)	10.82 (10.48-11.16)	11.16 (10.92-11.40)	10.89 (10.66-11.12)	10.64 (10.50-10.79)	10.46 (10.22-10.70)	10.56 (10.36-10.76)	11.53 (11.27-11.79)	11.56 (11.27-11.86)	<0.001	0.87 (0.33 to 1.42)
Overweight	10.79 (10.35-11.23)	10.71 (10.53-10.88)	11.04 (10.77-11.31)	11.24 (10.97-11.52)	11.01 (10.75-11.27)	10.82 (10.56-11.08)	10.76 (10.54-10.98)	11.33 (11.02-11.65)	11.55 (11.26-11.84)	11.76 (11.41-12.10)	<0.001	0.97 (0.41 to 1.53)
Obesity	11.58 (11.00-12.16)	10.90 (10.60-11.19)	11.55 (11.30-11.81)	11.51 (11.28-11.75)	11.40 (11.08-11.73)	11.05 (10.74-11.36)	11.05 (10.71-11.39)	11.26 (10.99-11.54)	11.85 (11.61-12.10)	12.06 (11.86-12.26)	<0.001	0.48 (-0.13 to 1.09)
p for interaction											<0.001[c]	<0.001[d]
Monounsaturated fat, % of energy												
Total population	12.19 (11.87-12.51)	12.19 (12.02-12.36)	12.65 (12.40-12.90)	12.38 (12.23-12.54)	12.4 (12.26-12.55)	11.88 (11.70-12.06)	11.78 (11.57-11.98)	11.81 (11.71-11.91)	12.42 (12.26-12.57)	12.41 (12.26-12.57)	<0.001	0.22 (-0.13 to 0.58)
Normal weight	11.85 (11.44-12.27)	11.87 (11.58-12.15)	12.27 (11.99-12.55)	12.25 (12.04-12.46)	12.03 (11.84-12.23)	11.54 (11.31-11.78)	11.44 (11.19-11.68)	11.2 (10.95-11.45)	12.33 (12.01-12.65)	12.24 (11.91-12.56)	<0.001	0.38 (-0.15 to 0.91)

Overweight	12.00 (11.48-12.51)	12.15 (11.85-12.45)	12.58 (12.17-12.99)	12.30 (12.03-12.57)	12.29 (12.07-12.50)	11.87 (11.63-12.12)	11.69 (11.41-11.97)	12.07 (11.87-12.27)	12.21 (12.03-12.38)	12.56 (12.29-12.83)	<0.001	0.56 (-0.02 to 1.14)
Obesity	12.74 (12.21-13.27)	12.58 (12.22-12.93)	13.11 (12.73-13.50)	12.57 (12.36-12.77)	12.83 (12.54-13.13)	12.11 (11.79-12.44)	12.17 (11.90-12.44)	12.03 (11.78-12.27)	12.65 (12.40-12.91)	12.41 (12.22-12.60)	<0.001	-0.33 (-0.90 to 0.24)
p for interaction											<0.001[c]	0.113[d]
Polyunsaturated fat, % of energy												
Total population	6.46 (6.24-6.68)	6.68 (6.55-6.80)	7.22 (7.03-7.40)	7.16 (7.00-7.32)	7.28 (7.18-7.39)	7.38 (7.27-7.50)	7.86 (7.77-7.96)	7.88 (7.76-8.00)	8.14 (7.99-8.29)	8.41 (8.30-8.53)	<0.001	1.96 (1.71 to 2.20)
Normal weight	6.18 (5.85-6.50)	6.50 (6.28-6.73)	7.07 (6.76-7.38)	6.98 (6.80-7.15)	7.17 (6.90-7.44)	7.34 (7.08-7.59)	7.68 (7.52-7.84)	7.62 (7.39-7.85)	8.03 (7.69-8.37)	8.42 (8.16-8.67)	<0.001	2.24 (1.83 to 2.65)
Overweight	6.59 (6.24-6.93)	6.68 (6.52-6.83)	7.22 (6.91-7.53)	7.20 (6.90-7.50)	7.18 (6.97-7.39)	7.42 (7.24-7.60)	7.74 (7.59-7.90)	7.98 (7.77-8.19)	7.88 (7.61-8.15)	8.40 (8.24-8.55)	<0.001	1.81 (1.43 to 2.19)
Obesity	6.64 (6.24-7.04)	6.86 (6.63-7.09)	7.36 (6.99-7.73)	7.28 (7.09-7.47)	7.48 (7.28-7.67)	7.38 (7.23-7.53)	8.14 (7.95-8.32)	8.00 (7.84-8.15)	8.41 (8.21-8.62)	8.42 (8.24-8.60)	<0.001	1.78 (1.35 to 2.22)
p for interaction											<0.001[c]	<0.001[d]
Polyunsaturated: saturated fat ratio												
Total population	0.64 (0.61-0.66)	0.68 (0.66-0.70)	0.69 (0.68-0.71)	0.68 (0.66-0.69)	0.70 (0.68-0.71)	0.72 (0.71-0.74)	0.78 (0.76-0.79)	0.75 (0.74-0.77)	0.74 (0.72-0.76)	0.76 (0.74-0.77)	<0.001	0.12 (0.09 to 0.15)
Normal weight	0.63 (0.58-0.68)	0.68 (0.64-0.71)	0.71 (0.68-0.74)	0.67 (0.66-0.69)	0.70 (0.67-0.73)	0.73 (0.70-0.76)	0.79 (0.76-0.81)	0.77 (0.75-0.80)	0.74 (0.71-0.77)	0.77 (0.74-0.80)	<0.001	0.14 (0.09 to 0.20)
Overweight	0.66 (0.60-0.72)	0.67 (0.65-0.70)	0.70 (0.68-0.72)	0.68 (0.65-0.72)	0.69 (0.66-0.72)	0.74 (0.72-0.76)	0.77 (0.75-0.79)	0.75 (0.71-0.78)	0.72 (0.69-0.76)	0.76 (0.74-0.79)	<0.001	0.10 (0.04 to 0.17)
Obesity	0.62 (0.58-0.67)	0.69 (0.67-0.71)	0.67 (0.64-0.70)	0.67 (0.65-0.69)	0.70 (0.68-0.72)	0.71 (0.69-0.73)	0.78 (0.75-0.80)	0.75 (0.73-0.77)	0.75 (0.73-0.78)	0.74 (0.72-0.76)	<0.001	0.12 (0.07 to 0.17)
p for interaction											<0.001[c]	<0.001[d]
Protein, % of energy												
Total population	15.04 (14.80-15.28)	15.08 (14.89-15.27)	15.47 (15.17-15.77)	16.01 (15.80-16.22)	15.92 (15.70-16.14)	16.07 (15.75-16.4)	15.68 (15.44-15.93)	16.03 (15.78-16.27)	15.81 (15.54-16.09)	15.65 (15.43-15.86)	<0.001	0.60 (0.28 to 0.93)
Normal weight	14.34 (13.90-	15.24 (14.94-	14.98 (14.65-	15.69 (15.41-	15.57 (15.09-	15.66 (15.28-	15.11 (14.77-	15.62 (15.14-	15.79 (15.43-	15.40 (14.98-	0.010	1.06 (0.45 to 1.67)

	14.78)	15.54)	15.32)	15.97)	16.04)	16.05)	15.45)	16.10)	16.15)	15.81)		
Overweight	15.53 (14.86-16.20)	15.07 (14.77-15.37)	15.68 (15.31-16.04)	15.89 (15.67-16.10)	15.80 (15.42-16.19)	16.06 (15.63-16.49)	15.94 (15.62-16.26)	16.36 (16.00-16.72)	15.79 (15.43-16.16)	15.70 (15.37-16.03)	0.057	0.17 (-0.58 to 0.92)
Obesity	15.33 (14.84-15.82)	14.95 (14.69-15.21)	15.78 (15.43-16.12)	16.42 (16.06-16.78)	16.33 (16.02-16.65)	16.38 (15.99-16.77)	15.91 (15.52-16.30)	16.02 (15.7-16.34)	15.82 (15.48-16.16)	15.75 (15.45-16.04)	<0.001	0.42 (-0.15 to 0.99)
p for interaction											<0.001[c]	<0.001[d]
Carbohydrate, % of energy												
Total population	50.98 (50.16-51.81)	50.75 (50.02-51.48)	49.25 (48.47-50.03)	48.73 (48.14-49.31)	49.1 (48.56-49.64)	49.14 (48.66-49.62)	49.38 (48.78-49.97)	48.00 (47.39-48.61)	46.93 (46.44-47.41)	46.24 (45.70-46.78)	<0.001	-4.75 (-5.73 to -3.76)
Normal weight	52.39 (51.52-53.26)	51.04 (49.80-52.27)	50.48 (49.51-51.46)	48.98 (48.22-49.74)	49.83 (49.02-50.64)	50.31 (49.70-50.92)	50.76 (49.94-51.57)	49.78 (48.91-50.64)	47.07 (46.39-47.75)	46.79 (45.90-47.67)	<0.001	-5.61 (-6.85 to -4.36)
Overweight	50.34 (49.03-51.65)	50.34 (49.48-51.19)	49.25 (48.31-50.18)	48.86 (48.03-49.69)	49.51 (48.87-50.16)	48.66 (47.98-49.33)	48.70 (48.15-49.25)	46.18 (45.27-47.08)	47.17 (46.07-48.27)	46.24 (45.33-47.15)	<0.001	-4.10 (-5.70 to -2.50)
Obesity	50.11 (48.95-51.27)	50.93 (49.92-51.93)	48.03 (46.82-49.24)	48.43 (47.51-49.34)	48.07 (47.11-49.04)	48.75 (47.80-49.69)	48.79 (47.84-49.74)	48.29 (47.61-48.96)	46.60 (45.88-47.31)	45.91 (45.32-46.49)	<0.001	-4.20 (-5.51 to -2.90)
p for interaction											<0.001[c]	<0.001[d]
Cholesterol, mg/d												
Total population	209.53 (198.95-220.10)	254.70 (246.75-262.66)	275.12 (266.28-283.95)	278.58 (270.41-286.75)	276.74 (268.45-285.04)	269.29 (262.78-275.80)	274.43 (266.23-282.64)	281.34 (271.52-291.16)	287.06 (277.29-296.84)	295.54 (286.48-304.60)	<0.001	86.01 (72.05 to 99.97)
Normal weight	195.10 (183.05-207.15)	247.89 (226.53-269.26)	262.81 (248.70-276.92)	272.54 (255.95-289.14)	251.98 (240.77-263.20)	246.32 (237.91-254.73)	254.72 (239.15-270.29)	257.73 (247.21-268.24)	272.24 (259.92-284.56)	278.38 (264.62-292.15)	<0.001	83.28 (64.93 to 101.64)
Overweight	212.30 (195.36-229.23)	257.36 (246.97-267.75)	281.33 (267.47-295.19)	278.74 (266.62-290.85)	272.52 (265.30-279.74)	276.47 (264.38-288.55)	281.87 (264.44-299.30)	293.37 (277.21-309.53)	291.92 (276.36-307.49)	301.54 (282.35-320.73)	<0.001	89.24 (63.54 to 114.95)
Obesity	221.62 (199.00-244.24)	259.55 (242.37-276.74)	281.31 (263.20-299.41)	283.68 (271.21-296.16)	301.87 (285.93-317.81)	279.63 (268.19-291.06)	284.37 (269.23-299.51)	288.05 (275.52-300.57)	292.55 (277.63-307.47)	300.98 (291.54-310.42)	<0.001	79.36 (54.82 to 103.89)

p for interaction											<0.001[c]	<0.001[d]
Dietary fiber, g/d												
Total population	12.15 (11.42-12.87)	14.22 (13.64-14.80)	14.44 (13.80-15.09)	15.35 (14.81-15.88)	15.37 (14.57-16.16)	16.25 (15.95-16.55)	17.25 (16.80-17.70)	16.33 (16.01-16.65)	16.60 (15.89-17.32)	15.63 (15.14-16.11)	<0.001	3.48 (2.60 to 4.35)
Normal weight	12.77 (11.81-13.73)	14.58 (13.74-15.42)	14.54 (13.58-15.50)	15.38 (14.56-16.20)	15.91 (14.79-17.03)	16.56 (15.92-17.20)	17.99 (17.29-18.69)	16.89 (16.18-17.60)	17.05 (16.15-17.95)	16.20 (15.27-17.13)	<0.001	3.43 (2.09 to 4.77)
Overweight	11.66 (10.68-12.64)	14.37 (13.38-15.35)	14.65 (13.90-15.40)	15.64 (14.84-16.44)	15.27 (14.28-16.26)	16.92 (16.50-17.35)	17.57 (16.99-18.14)	16.60 (15.86-17.33)	16.68 (15.53-17.84)	16.38 (15.67-17.08)	<0.001	4.72 (3.51 to 5.93)
Obesity	11.94 (10.86-13.01)	13.67 (13.05-14.29)	14.12 (13.44-14.80)	15.01 (14.69-15.33)	15.01 (14.14-15.89)	15.43 (14.94-15.92)	16.30 (15.72-16.87)	15.68 (15.05-16.31)	16.26 (15.58-16.95)	14.78 (14.30-15.26)	<0.001	2.84 (1.66 to 4.03)
p for interaction											<0.001[c]	<0.001[d]
Potassium, mg/d												
Total population	2291.27 (2157.41-2425.13)	2477.20 (2419.99-2534.41)	2505.74 (2439.55-2571.93)	2627.65 (2556.42-2698.88)	2539.04 (2458.89-2619.20)	2644.32 (2612.28-2676.37)	2668.58 (2618.28-2718.89)	2546.56 (2500.85-2592.28)	2523.18 (2456.18-2590.19)	2461.20 (2402.12-2520.28)	<0.001	169.94 (23.51 to 316.36)
Normal weight	2362.69 (2240.82-2484.55)	2538.47 (2423.53-2653.41)	2476.51 (2374.04-2578.98)	2657.27 (2544.02-2770.52)	2589.99 (2463.76-2716.22)	2615.55 (2544.14-2686.96)	2694.03 (2626.01-2762.05)	2557.17 (2492.75-2621.59)	2552.87 (2456.08-2649.65)	2468.66 (2367.27-2570.05)	<0.001	105.97 (-52.71 to 264.65)
Overweight	2296.07 (2116.81-2475.33)	2559.75 (2439.45-2680.04)	2556.96 (2475.66-2638.26)	2677.92 (2583.16-2772.67)	2515.82 (2428.35-2603.29)	2771.12 (2702.74-2839.49)	2717.39 (2620.74-2814.03)	2621.44 (2513.95-2728.94)	2562.65 (2444.00-2681.30)	2544.66 (2464.56-2624.76)	<0.001	248.59 (52.02 to 445.16)
Obesity	2201.85 (1977.96-2425.75)	2313.09 (2243.95-2382.23)	2480.30 (2398.91-2561.69)	2549.08 (2490.72-2607.44)	2520.14 (2414.60-2625.68)	2554.64 (2488.66-2620.63)	2599.54 (2500.88-2698.20)	2472.96 (2401.86-2544.05)	2475.34 (2404.23-2546.45)	2400.74 (2326.80-2474.67)	<0.001	198.88 (-36.99 to 434.76)
p for interaction											<0.001[c]	<0.001[d]
Magnesium, mg/d												
Total population	235.64 (222.93-248.35)	248.88 (241.31-256.46)	256.25 (247.35-265.14)	284.53 (276.27-292.78)	276.43 (264.85-288.01)	288.99 (284.66-293.31)	295.45 (288.52-302.38)	286.17 (280.52-291.83)	289.48 (279.26-299.70)	284.23 (277.42-291.05)	<0.001	48.59 (34.14 to 63.03)

Normal weight	243.58 (229.38-257.77)	258.32 (245.76-270.89)	257.06 (243.90-270.22)	289.28 (277.49-301.06)	284.78 (268.97-300.59)	288.44 (280.70-296.18)	302.84 (293.41-312.27)	295.53 (284.66-306.41)	298.09 (286.44-309.75)	286.86 (274.87-298.85)	<0.001	43.29 (24.66 to 61.91)
Overweight	237.06 (219.27-254.86)	252.84 (239.30-266.38)	261.49 (250.53-272.45)	291.64 (280.28-302.99)	275.34 (259.88-290.80)	303.89 (293.89-313.89)	300.39 (290.76-310.03)	293.70 (281.64-305.76)	293.11 (278.85-307.36)	294.23 (284.20-304.26)	<0.001	57.17 (36.68 to 77.65)
Obesity	225.35 (205.84-244.86)	234.24 (226.35-242.12)	249.83 (241.33-258.33)	273.24 (266.69-279.78)	270.54 (257.52-283.55)	276.35 (268.97-283.73)	284.30 (274.33-294.27)	272.66 (265.73-279.60)	281.27 (271.17-291.36)	275.80 (268.08-283.53)	<0.001	50.46 (29.47 to 71.44)
p for interaction											<0.001[c]	<0.001[d]
Calcium, mg/d												
Total population	687.35 (642.98-731.71)	759.90 (734.97-784.83)	793.22 (765.91-820.53)	890.19 (863.44-916.93)	870.13 (835.09-905.17)	949.63 (934.83-964.43)	949.77 (922.71-976.84)	917.94 (900.83-935.06)	922.64 (888.32-956.96)	903.05 (883.58-922.52)	<0.001	215.70 (167.21 to 264.19)
Normal weight	720.16 (681.65-758.66)	794.75 (746.03-843.48)	811.91 (774.88-848.95)	885.64 (840.09-931.19)	891.27 (839.70-942.84)	928.80 (891.74-965.86)	973.69 (939.66-1007.71)	930.05 (897.71-962.39)	934.11 (896.95-971.27)	877.81 (850.09-905.53)	<0.001	157.65 (110.07 to 205.23)
Overweight	648.77 (594.61-702.93)	774.17 (736.97-811.36)	785.98 (742.02-829.95)	908.33 (867.98-948.67)	860.6 (832.34-888.86)	993.82 (954.48-1033.16)	941.17 (913.14-969.19)	923.29 (890.01-956.58)	943.47 (880.53-1006.42)	907.73 (887.44-928.02)	<0.001	258.95 (201.01 to 316.9)
Obesity	688.10 (601.62-774.58)	703.02 (658.91-747.12)	780.52 (749.88-811.16)	874.85 (848.52-901.18)	862.07 (808.70-915.45)	927.13 (907.01-947.25)	937.98 (884.84-991.12)	904.84 (866.47-943.20)	900.49 (872.04-928.94)	914.75 (871.50-958.00)	<0.001	226.65 (129.76 to 323.54)
p for interaction											<0.001[c]	<0.001[d]

[a] Normal weight is defined as a body mass index (BMI) ranging from 18.5 to 24.9, overweight is defined as a BMI ranging from 25 to 29.9, and obesity is defined as a BMI ≥ 30.

[b] Survey-weighted mean dietary intake of key food groups and nutrients were adjusted for age, sex, and race/ethnicity. [c] *p* for interaction assessing potential heterogeneous trends in dietary intake of key food groups and nutrients by body weight status. [d] *p* for interaction assessing potential heterogeneous changes in dietary intake of key food groups and nutrients from 1999-2000 to 2017-2020 by body weight status.

Table S7. Mean Change in Key Food Groups Intake among Obese Participants from 1999-2000 to 2017-2020, by Age[a]

Dietary Intake	Survey-Weighted Mean Intake or Change (95% CI)[b]												p for Interaction
	Age 20-34		Mean Change (95% CI)	Age 35-49		Mean Change (95% CI)	Age 50-64		Mean Change (95% CI)	Age ≥65		Mean Change (95% CI)	
	1999-2000 (*n* = 108)	2017-2020 (*n* = 513)		1999-2000 (*n* = 119)	2017-2020 (*n* = 625)		1999-2000 (*n* = 105)	2017-2020 (*n* = 731)		1999-2000 (*n* = 108)	2017-2020 (*n* = 585)		
Total fruits, servings/d	0.31 (0.21-0.42)	0.31 (0.26-0.37)	0.00 (-0.12 to 0.11)	0.73 (0.38-1.09)	0.45 (0.38-0.52)	-0.28 (-0.64 to 0.08)	1.07 (0.71-1.43)	0.42 (0.35-0.50)	-0.65*** (-1.01 to -0.28)	1.08 (0.81-1.34)	0.58 (0.51-0.64)	-0.50*** (-0.77 to -0.22)	<0.001
Whole fruits, servings/d	0.15 (0.06-0.25)	0.22 (0.17-0.28)	0.07 (-0.04 to 0.18)	0.45 (0.18-0.72)	0.34 (0.27-0.41)	-0.11 (-0.39 to 0.17)	0.55 (0.34-0.77)	0.32 (0.26-0.38)	-0.23* (-0.45 to -0.01)	0.72 (0.57-0.87)	0.46 (0.39-0.52)	-0.26** (-0.42 to -0.10)	<0.001
Total vegetables, servings/d	2.55 (1.74-3.37)	0.67 (0.61-0.73)	-1.88*** (-2.70 to -1.06)	2.94 (1.76-4.13)	0.81 (0.73-0.88)	-2.13*** (-3.32 to -0.95)	2.57 (2.23-2.91)	0.78 (0.68-0.88)	-1.79*** (-2.15 to -1.43)	3.58 (2.89-4.28)	0.88 (0.80-0.96)	-2.71*** (-3.40 to -2.01)	<0.001
Greens and Beans, servings/d	0.11 (0.05-0.17)	0.10 (0.08-0.11)	-0.01 (-0.07 to 0.05)	0.28 (0.16-0.40)	0.12 (0.10-0.14)	-0.16* (-0.29 to -0.04)	0.17 (0.07-0.26)	0.11 (0.09-0.14)	-0.05 (-0.15 to 0.05)	0.17 (0.00-0.34)	0.11 (0.09-0.14)	-0.06 (-0.23 to 0.12)	<0.001
Whole grains, servings/d	0.26 (0.16-0.35)	0.28 (0.22-0.33)	0.02 (-0.09 to 0.13)	0.52 (0.27-0.77)	0.34 (0.29-0.40)	-0.18 (-0.44 to 0.07)	0.24 (0.07-0.40)	0.39 (0.31-0.48)	0.15 (-0.03 to 0.34)	0.43 (0.26-0.59)	0.58 (0.48-0.68)	0.16 (-0.03 to 0.35)	<0.001
Dairy, servings/d	2.81 (1.91-3.72)	0.68 (0.60-0.76)	-2.13*** (-3.04 to -1.22)	2.00 (1.60-2.40)	0.73 (0.60-0.85)	-1.27*** (-1.69 to -0.85)	1.69 (1.27-2.10)	0.67 (0.61-0.74)	-1.01*** (-1.43 to -0.60)	2.09 (1.59-2.60)	0.71 (0.65-0.78)	-1.38*** (-1.89 to -0.87)	<0.001
Total protein foods, servings/d	10.93 (7.98-	3.10 (2.91-3.29)	-7.83*** (-10.79 to -	9.42 (7.62-11.21)	3.46 (3.21-3.71)	-5.96*** (-7.77 to -	9.67 (7.99-11.34)	3.24 (3.06-3.43)	-6.42*** (-8.11 to -	9.57 (7.84-11.29)	3.18 (3.01-3.34)	-6.39*** (-8.12 to -	<0.001

	13.88)		4.87)			4.15)			4.74)			4.66)	
Seafood and plant proteins, servings/d	1.76 (0.88-2.65)	0.60 (0.50-0.70)	-1.16* (-2.05 to -0.27)	2.05 (1.26-2.85)	0.85 (0.70-1.01)	-1.20** (-2.01 to -0.39)	2.22 (1.25-3.18)	0.78 (0.67-0.89)	-1.43** (-2.40 to -0.47)	1.68 (0.77-2.60)	0.93 (0.79-1.07)	-0.75 (-1.68 to 0.17)	<0.001
Fatty acids	1.75 (1.62-1.89)	1.91 (1.83-1.98)	0.16* (0.00 to 0.31)	1.74 (1.58-1.90)	1.83 (1.75-1.92)	0.09 (-0.09 to 0.27)	1.77 (1.65-1.89)	1.90 (1.83-1.96)	0.13 (-0.01 to 0.26)	1.89 (1.76-2.03)	1.89 (1.82-1.96)	0.00 (-0.15 to 0.15)	0.104
Refined grains, servings/d	11.46 (9.40-13.51)	3.10 (2.92-3.27)	-8.36*** (-10.42 to -6.29)	8.39 (6.48-10.29)	2.57 (2.42-2.72)	-5.82*** (-7.72 to -3.91)	8.29 (6.88-9.70)	2.69 (2.49-2.89)	-5.60*** (-7.03 to -4.17)	7.55 (6.00-9.10)	2.39 (2.24-2.54)	-5.16*** (-6.72 to -3.61)	<0.001
Sodium, g/d	3.06 (2.78-3.34)	1.71 (1.66-1.76)	-1.35*** (-1.63 to -1.07)	2.85 (2.44-3.25)	1.71 (1.65-1.77)	-1.14*** (-1.55 to -0.73)	2.77 (2.49-3.06)	1.69 (1.62-1.76)	-1.08*** (-1.38 to -0.79)	2.53 (2.18-2.88)	1.69 (1.64-1.75)	-0.84*** (-1.19 to -0.49)	<0.001
Added sugars, tsp equivalents/d, servings/d	27.25 (24.09-30.41)	13.04 (11.64-14.44)	-14.21*** (-17.67 to -10.75)	21.03 (18.60-23.47)	12.62 (11.13-14.10)	-8.42*** (-11.27 to -5.57)	17.96 (16.32-19.61)	12.49 (11.00-13.98)	-5.47*** (-7.69 to -3.25)	13.73 (11.78-15.67)	10.98 (10.07-11.89)	-2.75* (-4.90 to -0.60)	<0.001
Saturated fats, % of energy	11.20 (10.43-11.97)	11.55 (11.02-12.08)	0.35 (-0.58 to 1.29)	11.60 (10.44-12.76)	12.20 (11.63-12.77)	0.60 (-0.69 to 1.89)	12.22 (11.38-13.05)	11.90 (11.45-12.35)	-0.32 (-1.26 to 0.63)	11.32 (10.79-11.85)	12.30 (11.78-12.82)	0.99* (0.24 to 1.73)	0.213

Abbreviations: HEI, Healthy Eating Index. [a] Obesity is defined as a BMI ≥ 30. [b] The mean change from 1999-2000 to 2017-2020 was statistically significant (* $p < 0.05$; ** $p < 0.01$; *** $p < 0.001$).

Table S8. Mean Change in Key Food Groups Intake among Obese Participants from 1999-2000 to 2017-2020, by Gender[a]

Dietary Intake	Survey-Weighted Mean Intake or Change (95% CI)[b]						*p* for Interaction
	Male		Mean Change (95% CI)	Female		Mean Change (95% CI)	
	1999-2000 (*n* = 159)	2017-2020 (*n* = 1075)		1999-2000 (*n* = 281)	2017-2020 (*n* = 1379)		
Total fruits, servings/d	0.73 (0.47-1.00)	0.40 (0.35-0.45)	-0.34* (-0.61 to -0.06)	0.72 (0.50-0.94)	0.47 (0.43-0.51)	-0.24* (-0.47 to -0.02)	0.018
Whole fruits, servings/d	0.38 (0.15-0.60)	0.30 (0.25-0.35)	-0.08 (-0.31 to 0.15)	0.45 (0.34-0.55)	0.36 (0.32-0.40)	-0.09 (-0.20 to 0.03)	0.098
Total vegetables, servings/d	2.96 (2.23-3.69)	0.73 (0.68-0.78)	-2.23*** (-2.96 to -1.50)	2.76 (2.04-3.48)	0.83 (0.78-0.88)	-1.93*** (-2.65 to -1.21)	<0.001
Greens and Beans, servings/d	0.20 (0.03-0.37)	0.10 (0.09-0.11)	-0.10 (-0.27 to 0.07)	0.18 (0.08-0.27)	0.12 (0.10-0.14)	-0.06 (-0.15 to 0.04)	<0.001
Whole grains, servings/d	0.34 (0.23-0.46)	0.38 (0.32-0.43)	0.03 (-0.09 to 0.16)	0.38 (0.27-0.50)	0.41 (0.36-0.45)	0.03 (-0.10 to 0.15)	0.666
Dairy, servings/d	2.81 (2.00-3.61)	0.69 (0.61-0.76)	-2.12*** (-2.93 to -1.31)	1.81 (1.45-2.16)	0.71 (0.67-0.75)	-1.10*** (-1.46 to -0.74)	<0.001
Total protein foods, servings/d	11.46 (9.30-13.62)	3.39 (3.20-3.58)	-8.07*** (-10.25 to -5.90)	8.98 (7.63-10.32)	3.13 (3.03-3.22)	-5.85*** (-7.20 to -4.50)	<0.001
Seafood and plant proteins, servings/d	2.18 (1.04-3.32)	0.78 (0.72-0.84)	-1.41* (-2.55 to -0.26)	1.78 (1.18-2.38)	0.80 (0.70-0.90)	-0.98** (-1.59 to -0.37)	<0.001
Fatty acids	1.73 (1.63-1.83)	1.88 (1.82-1.94)	0.15* (0.03 to 0.27)	1.80 (1.68-1.93)	1.88 (1.83-1.94)	0.08 (-0.05 to 0.22)	0.032
Refined grains, servings/d	11.28 (9.14-	2.67 (2.55-2.79)	-8.61***	7.83 (6.56-9.10)	2.71 (2.62-2.81)	-5.12***	<0.001

	13.42)		(-10.75 to -6.46)			(-6.39 to -3.84)	
Sodium, g/d	3.38 (3.04-3.73)	1.70 (1.65-1.76)	-1.68*** (-2.03 to -1.33)	2.50 (2.30-2.70)	1.70 (1.66-1.74)	-0.80*** (-1.00 to -0.60)	<0.001
Added sugars, tsp equivalents/d, servings/d	21.72 (18.56-24.88)	11.84 (10.85-12.83)	-9.88*** (-13.19 to -6.67)	20.92 (19.75-22.09)	12.77 (11.88-13.66)	-8.16*** (-9.63 to -6.69)	<0.001
Saturated fats, % of energy	11.65 (11.08-12.23)	11.90 (11.53-12.27)	0.25 (-0.44 to 0.93)	11.49 (10.73-12.26)	12.05 (11.76-12.35)	0.56 (-0.26 to 1.38)	0.355

Abbreviations: HEI, Healthy Eating Index. [a] Obesity is defined as a BMI ≥ 30. [b] The mean change from 1999-2000 to 2017-2020 was statistically significant (* $p < 0.05$; ** $p < 0.01$; *** $p < 0.001$).

Table S9. Mean Change in Key Food Groups Intake among Obese Participants from 1999-2000 to 2017-2020, by Race/Ethnicity[a]

Dietary Intake	Survey-Weighted Mean Intake or Change (95% CI)[b]									p for Interaction
	Non-Hispanic White		Mean Change (95% CI)	Non-Hispanic Black		Mean Change (95% CI)	Hispanic		Mean Change (95% CI)	
	1999-2000 (*n* = 176)	2017-2020 (*n* = 922)		1999-2000 (*n* = 114)	2017-2020 (*n* = 767)		1999-2000 (*n* = 16)	2017-2020 (*n* = 243)		
Total fruits, servings/d	0.76 (0.51-1.02)	0.42 (0.38-0.47)	-0.34* (-0.60 to -0.08)	0.73 (0.53-0.92)	0.44 (0.37-0.51)	-0.28** (-0.49 to -0.08)	0.62 (0.21-1.03)	0.55 (0.44-0.65)	-0.07 (-0.49 to 0.35)	0.032
Whole fruits, servings/d	0.47 (0.31-0.63)	0.33 (0.29-0.37)	-0.13 (-0.30 to 0.03)	0.38 (0.23-0.54)	0.28 (0.22-0.34)	-0.10 (-0.27 to 0.07)	0.21 (0.00-0.42)	0.37 (0.27-0.46)	0.16 (-0.07 to 0.39)	0.076
Total vegetables, servings/d	3.05 (2.33-3.76)	0.78 (0.72-0.84)	-2.27*** (-2.99 to -1.55)	1.96 (1.65-2.27)	0.75 (0.70-0.81)	-1.20*** (-1.52 to -0.89)	2.71 (0.76-4.66)	0.84 (0.74-0.94)	-1.87 (-3.82 to 0.09)	<0.001
Greens and Beans, servings/d	0.19 (0.15-0.22)	0.09 (0.08-0.11)	-0.10*** (-0.13 to -0.06)	0.12 (0.05-0.20)	0.14 (0.12-0.17)	0.02 (-0.06 to 0.10)	0.08 (-0.07-0.23)	0.15 (0.12-0.19)	0.08 (-0.08 to 0.23)	<0.001
Whole grains, servings/d	0.43 (0.32-0.54)	0.42 (0.38-0.47)	-0.01 (-0.13 to 0.11)	0.29 (0.08-0.50)	0.33 (0.27-0.38)	0.04 (-0.18 to 0.26)	0.13 (0.00-0.26)	0.38 (0.28-0.48)	0.25** (0.09 to 0.41)	0.006
Dairy, servings/d	2.27 (1.85-2.68)	0.76 (0.69-0.83)	-1.51*** (-1.92 to -1.09)	1.76 (1.09-2.44)	0.52 (0.48-0.56)	-1.24*** (-1.92 to -0.57)	2.16 (1.12-3.20)	0.65 (0.59-0.71)	-1.51** (-2.55 to -0.47)	<0.001
Total protein foods, servings/d	9.74 (8.47-11.00)	3.13 (2.98-3.28)	-6.61*** (-7.88 to -5.34)	11.02 (9.17-12.87)	3.49 (3.35-3.62)	-7.54*** (-9.39 to -5.68)	11.07 (4.32-17.81)	3.46 (3.25-3.68)	-7.60* (-14.35 to -0.85)	<0.001
Seafood and plant proteins, servings/d	1.97 (1.47-2.47)	0.77 (0.68-0.85)	-1.20*** (-1.71 to -0.70)	1.67 (0.76-2.59)	0.84 (0.74-0.93)	-0.84 (-1.76 to 0.08)	2.11 (0.25-3.96)	0.89 (0.75-1.03)	-1.22 (-3.08 to 0.64)	<0.001
Fatty acids	1.78 (1.68-1.87)	1.84 (1.79-1.89)	0.06 (-0.04 to 0.17)	1.86 (1.70-2.02)	2.08 (2.02-2.14)	0.22* (0.05 to 0.39)	1.40 (1.19-1.60)	1.88 (1.78-1.97)	0.48*** (0.26 to 0.70)	0.029
Refined grains,	8.63 (7.60-9.67)	2.61 (2.50-2.71)	-6.03***	10.08 (8.13-	2.49 (2.36-2.62)	-7.59***	12.16 (5.79-	2.95 (2.76-3.14)	-9.21**	<0.001

servings/d			(-7.06 to -4.99)	12.02)		(-9.54 to -5.64)	18.53)		(-15.58 to -2.84)	
Sodium, g/d	2.97 (2.68-3.26)	1.71 (1.66-1.75)	-1.26*** (-1.55 to -0.97)	2.49 (2.23-2.75)	1.68 (1.62-1.74)	-0.81*** (-1.07 to -0.55)	3.17 (2.31-4.02)	1.61 (1.54-1.67)	-1.56*** (-2.42 to -0.70)	<0.001
Added sugars, tsp equivalents/d, servings/d	18.29 (15.89-20.70)	12.17 (11.17-13.16)	-6.12*** (-8.73 to -3.52)	28.05 (24.85-31.25)	13.59 (12.82-14.36)	-14.46*** (-17.76 to -11.17)	28.70 (20.84-36.55)	11.33 (10.11-12.56)	-17.36*** (-25.31 to -9.41)	<0.001
Saturated fats, % of energy	11.93 (11.34-12.53)	12.31 (12.04-12.59)	0.38 (-0.27 to 1.04)	10.61 (9.77-11.44)	11.35 (11.11-11.59)	0.74 (-0.13 to 1.61)	11.49 (9.43-13.54)	11.22 (10.71-11.72)	-0.27 (-2.39 to 1.84)	0.057

Abbreviations: HEI, Healthy Eating Index. [a] Obesity is defined as a BMI ≥ 30. [b] The mean change from 1999-2000 to 2017-2020 was statistically significant (* $p < 0.05$; ** $p < 0.01$; *** $p < 0.001$).

Table S10. Mean Change in Key Food Groups Intake among Obese Participants from 1999-2000 to 2017-2020, by Education[a]

Dietary Intake	Survey-Weighted Mean Intake or Change (95% CI)[b]						*p* for Interaction
	High School or Less than High School Education		Mean Change (95% CI)	Some college, College or Above Education		Mean Change (95% CI)	
	1999-2000 (*n* = 285)	2017-2020 (*n* = 1015)		1999-2000 (*n* = 155)	2017-2020 (*n* = 1439)		
Total fruits, servings/d	0.67 (0.47-0.87)	0.63 (0.50-0.75)	-0.04 (-0.28 to 0.20)	0.76 (0.33-1.19)	0.53 (0.37-0.70)	-0.23 (-0.69 to 0.23)	0.015
Whole fruits, servings/d	0.42 (0.03-0.54)	0.50 (0.37-0.63)	0.08 (-0.09 to 0.26)	0.49 (0.12-0.85)	0.42 (0.26-0.58)	-0.07 (-0.47 to 0.34)	0.397
Total vegetables, servings/d	4.36 (2.90-5.83)	0.96 (0.77-1.15)	-3.40*** (-4.88 to -1.92)	3.33 (1.69-4.97)	0.74 (0.66-0.81)	-2.59** (-4.23 to -0.95)	<0.001
Greens and Beans, servings/d	0.44 (0.30-0.58)	0.20 (0.13-0.27)	-0.24** (-0.40 to -0.08)	0.33 (0.09-0.56)	0.11 (0.09-0.13)	-0.22 (-0.45 to 0.02)	<0.001
Whole grains, servings/d	0.05 (0.01-0.09)	0.49 (0.34-0.65)	0.44*** (0.28 to 0.60)	0.38 (0.14-0.62)	0.35 (0.20-0.49)	-0.03 (-0.31 to 0.25)	0.171
Dairy, servings/d	1.68 (0.78-2.59)	0.59 (0.50-0.67)	-1.10* (-2.00 to -0.19)	1.81 (1.42-2.20)	0.65 (0.52-0.78)	-1.16*** (-1.57 to -0.74)	<0.001
Total protein foods, servings/d	9.10 (7.45-10.75)	3.14 (2.83-3.45)	-5.96*** (-7.64 to -4.28)	10.07 (7.16-12.98)	3.30 (2.89-3.72)	-6.77*** (-9.71 to -3.83)	<0.001
Seafood and plant proteins, servings/d	2.59 (1.24-3.94)	0.92 (0.72-1.13)	-1.67* (-3.03 to -0.30)	2.79 (1.03-4.55)	0.77 (0.50-1.03)	-2.02* (-3.80 to -0.24)	<0.001
Fatty acids	1.82 (1.63-2.01)	1.91 (1.70-2.11)	0.09 (-0.19 to 0.37)	1.81 (1.65-1.96)	1.90 (1.78-2.03)	0.10 (-0.10 to 0.29)	0.067

Refined grains, servings/d	7.99 (5.44-10.54)	3.01 (2.48-3.55)	-4.97*** (-7.58 to -2.37)	8.56 (6.64-10.49)	2.67 (2.41-2.92)	-5.90*** (-7.84 to -3.96)	<0.001
Sodium, g/d	2.38 (1.86-2.90)	1.63 (1.51-1.75)	-0.76** (-1.29 to -0.22)	2.81 (2.24-3.38)	1.64 (1.57-1.71)	-1.17*** (-1.74 to -0.59)	<0.001
Added sugars, tsp equivalents/d, servings/d	23.51 (20.25-26.76)	13.47 (10.15-16.79)	-10.04*** (-14.69 to -5.39)	22.57 (18.44-26.70)	12.97 (11.49-14.45)	-9.60*** (-13.99 to -5.21)	<0.001
Saturated fats, % of energy	10.74 (9.65-11.83)	11.25 (9.80-12.71)	0.51 (-1.31 to 2.33)	10.96 (9.90-12.02)	11.75 (11.17-12.33)	0.79 (-0.42 to 1.99)	0.187

Abbreviations: HEI, Healthy Eating Index. [a] Obesity is defined as a BMI ≥ 30. [b] The mean change from 1999-2000 to 2017-2020 was statistically significant (* $p < 0.05$; ** $p < 0.01$; *** $p < 0.001$).
